# Toripalimab, bevacizumab, and irinotecan in dMMR/MSI locally advanced colorectal cancer: First-stage results from a phase 1b/2 trial

**DOI:** 10.1016/j.xcrm.2025.102296

**Published:** 2025-08-15

**Authors:** Zhenghang Wang, Xicheng Wang, Xiaoyan Zhang, Jiahua Leng, Ming Cui, Ji Zhang, Quan Wang, Yu Sun, Ting Xu, Mifen Chen, Jian Li, Lin Shen

**Affiliations:** 1 State Key Laboratory of Holistic Integrative Management of Gastrointestinal Cancers, Beijing Key Laboratory of Carcinogenesis and Translational Research, Department of Gastrointestinal Oncology, Peking University Cancer Hospital & Institute, Beijing 100142, China; 2 Key Laboratory of Carcinogenesis and Translational Research (Ministry of Education/Beijing), Department of Gastrointestinal Oncology, Peking University Cancer Hospital & Institute, Beijing 100142, China; 3 Key Laboratory of Carcinogenesis and Translational Research (Ministry of Education/Beijing), Department of Radiology, Peking University Cancer Hospital & Institute, Beijing 100142, China; 4 Key Laboratory of Carcinogenesis and Translational Research (Ministry of Education/Beijing), Gastrointestinal Cancer Center, Unit III, Peking University Cancer Hospital & Institute, Beijing 100142, China; 5 Key Laboratory of Carcinogenesis and Translational Research (Ministry of Education/Beijing), Gastrointestinal Cancer Center, Unit IV, Peking University Cancer Hospital & Institute, Beijing 100142, China; 6 Key Laboratory of Carcinogenesis and Translational Research (Ministry of Education/Beijing), Gastrointestinal Cancer Center, Unit II, Peking University Cancer Hospital & Institute, Beijing 100142, China; 7 The First Hospital of Jilin University, Department of Gastrointestinal Surgery, Chang Chun 130031, China; 8 State Key Laboratory of Holistic Integrative Management of Gastrointestinal Cancers, Beijing Key Laboratory of Carcinogenesis and Translational Research, Department of Pathology, Peking University Cancer Hospital & Institute, Beijing 100142, China; 9 Key Laboratory of Carcinogenesis and Translational Research (Ministry of Education/Beijing), Department of Pathology, Peking University Cancer Hospital & Institute, Beijing 100142, China

**Keywords:** colon cancer, rectal cancer, microsatellite instability, neoadjuvant therapy, immune checkpoint inhibitor, chemotherapy, anti-VEGF, complete pathological response, survival

## Abstract

This is the first stage of the phase 1b/2 trial evaluating the effectiveness and safety of toripalimab, irinotecan, and bevacizumab in patients with rectal cancer refusing up-front surgery or radiation therapy (rectum cohort) and patients with T4NanyM0 colon cancer (colon cohort) with deficiency of mismatch repair (dMMR) or microsatellite instability (MSI). This trial allows a doctor-patient shared decision-making process to determine whether to omit irinotecan or bevacizumab and the optimal surgery timing. The primary endpoint pathological complete response (pCR) rates in the full analysis set (FAS) and per-protocol set (PPS) are 57.1% (95% confidence interval [CI] 28.9–82.3) and 66.7% (34.9–90.1), respectively, in the colon cohort (*n* = 14) and 75.0% (35.6–95.5) and 100% (51.7–100.0), respectively, in the rectum cohort (*n* = 8). No disease recurrence occurs in PPS. No grade 4–5 drug-related adverse events are observed. Toripalimab with or without irinotecan and bevacizumab shows promising efficacy and manageable toxicity in dMMR/MSI T4NanyM0 colon cancer and locally advanced rectal cancer (ClinicalTrials.gov: NCT04988191).

## Highlights

• Toripalimab plus irinotecan and bevacizumab has a promising pCR rate
• Toxicity is manageable, and long-term survival benefit is observed
• More doses of toripalimab are associated with a higher pCR rate
• Irinotecan and bevacizumab appear to increase objective response rate but not pCR rate

## Graphical abstract

**Figure:**
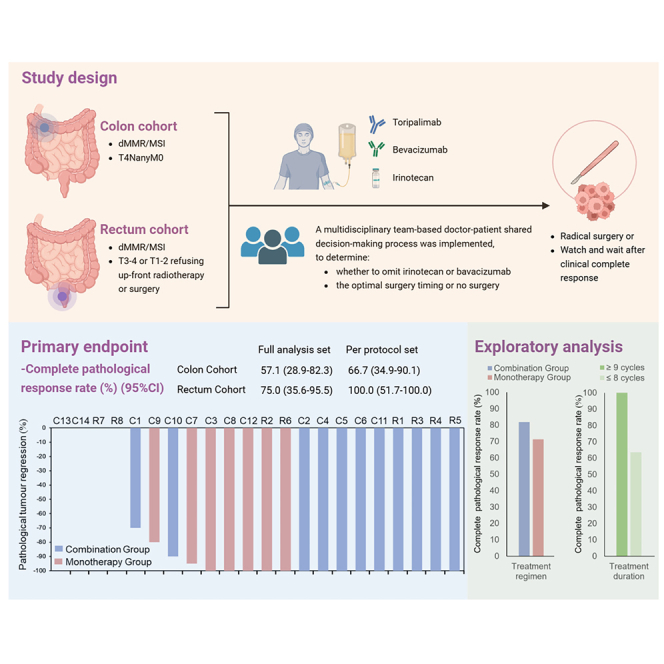

## Teaser

Wang et al. show that toripalimab plus irinotecan and bevacizumab is a safe and active regimen in patients with T4NanyM0 colon cancer or locally advanced rectal cancer with deficiency of mismatch repair or microsatellite instability. The potential contribution of chemotherapy and anti-VEGF treatment to PD-1 antibody should be further evaluated.

## Introduction

Colorectal cancer (CRC) ranks as the third most commonly diagnosed cancer and the second leading cause of cancer-related mortality worldwide.^1^ In the context of metastatic CRCs characterized by the deficiency of mismatch repair (dMMR) or microsatellite instability (MSI), immune checkpoint inhibitors (ICIs) such as programmed death 1 (PD-1) or programmed death-ligand 1 (PD-L1) antibodies, either alone or in combination with cytotoxic T lymphocyte associated protein 4 (CTLA-4) antibodies, have demonstrated remarkable efficacy in late-line treatment across a growing number of clinical trials.^2^^,^^3^^,^^4^^,^^5^ More recently, pembrolizumab and atezolizumab have exhibited superior outcomes in terms of progression-free survival and overall survival compared to standard treatments in first-line and second-line settings, respectively.^6^^,^^7^

The compelling results obtained thus far have led to the exploration of ICI use in the preoperative setting for locally advanced CRC with dMMR/MSI. In the case of colon cancer, dMMR/MSI is present in 18.9%–21.3% of stage II tumors and 14.3%–14.4% of stage III tumors.^8^^,^^9^ In NICHE and NICHE-2 study, pathological complete response (pCR) was observed in 60% (12/20) and 67% (72/107), respectively, of patients with stage I–III dMMR colon cancers who were treated with two doses of nivolumab and one dose of ipilimumab.^2^^,^^10^ In the PICC study, which included 30 dMMR colon tumors, 23 patients (77%) achieved pCR following 3 months of treatment with toripalimab, with or without celecoxib.^11^ Notably, no tumor progression was observed during the neoadjuvant treatment in these trials. Although long-term follow-up data are limited, it appears that preoperative ICIs may also extend disease-free survival.^2^^,^^12^ These encouraging findings demonstrate the potential of ICIs in dMMR colon tumors, particularly those at the T4 stage, which is associated with a poor prognosis^13^ and exhibits limited response to neoadjuvant chemotherapy.^14^

In the case of rectal cancer, only a small proportion of patients (4%–10%) are identified as having dMMR/MSI.^15^^,^^16^ Current standard practice involves neoadjuvant chemotherapy and radiotherapy for resectable T3–4 rectal cancers, as well as for T1–2 rectal cancers unfit for surgery. However, dMMR/MSI locally advanced rectal cancer (LARC) may exhibit progression during neoadjuvant treatment or relapse following surgery subsequent to neoadjuvant chemotherapy or chemoradiotherapy.^17^ Furthermore, patients may be subjected to ostomy and experience bowel, urinary, or sexual dysfunction as a result of perioperative treatment. Recent studies have demonstrated the high efficacy of PD-1 blockade in stage II–III dMMR/MSI rectal cancers, with notable rates of clinical complete response (cCR) or pCR.^18^^,^^19^ These findings have opened up the possibility of sparing chemotherapy, radiotherapy, and even surgery for patients with dMMR/MSI LARC.

Despite the promising outcomes, the available data on neoadjuvant ICIs in dMMR/MSI colon and rectal tumors remain limited. Prior to the initiation of this trial, only the results with nivolumab plus ipilimumab had been published,^10^ but ipilimumab was not accessible in China. Given that 29.4% of patients with dMMR/MSI metastatic CRC experienced early progression beyond pembrolizumab in the Keynote-177 trial,^7^ concerns arose regarding the potential lack of benefit for all patients from PD-1 antibody monotherapy in the neoadjuvant setting, and there are no clinical trials combining chemotherapy with PD-1 antibody for neoadjuvant therapy.^20^ Consequently, the decision was made to combine irinotecan and bevacizumab with toripalimab, based on their immunomodulatory effects.^21^^,^^22^^,^^23^^,^^24^

This study aimed to investigate the clinical activity and safety of neoadjuvant treatment with toripalimab in combination with irinotecan and bevacizumab in patients diagnosed with dMMR/MSI T4NanyM0 colon cancer (T4CC) and LARC. During the design of this trial, we acknowledged the potential publication of data from ongoing studies, which could provide additional evidence for neoadjuvant ICIs. Consequently, we implemented a multidisciplinary team-based doctor-patient shared decision-making process to determine whether to include irinotecan or bevacizumab in the treatment regimen, as well as when and whether to proceed with surgery. This process enabled the evaluation of the efficacy of toripalimab with or without irinotecan or bevacizumab and facilitated an exploratory analysis of whether the pCR rate was associated with therapeutic cycles.

## Results

### Patient characteristics

**Figure 1:**
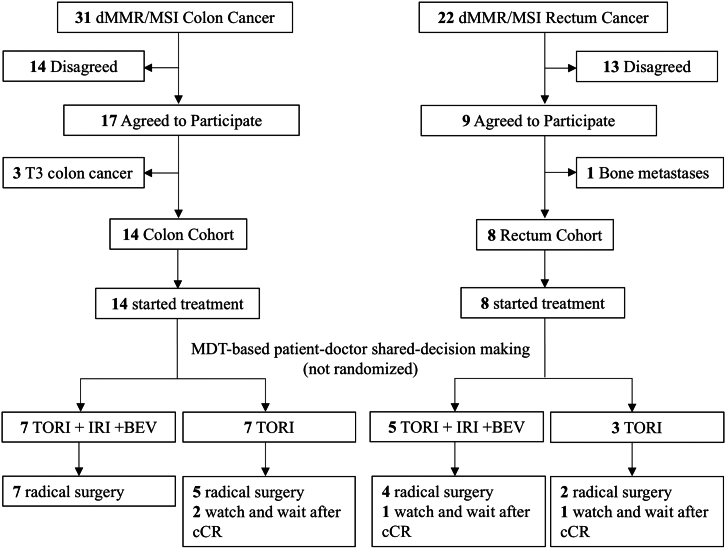
Study flow chart Patients were enrolled from Beijing Cancer Hospital and The First Hospital of Jilin University. MDT, multidisciplinary team; TORI, toripalimab; IRI, irinotecan; BEV, bevacizumab; cCR, clinical complete response.

From December 2020 to February 2023, a total of 26 patients were screened at two centers, with 22 of them deemed eligible for enrollment (Figure 1). The median age of the eligible patients was 47 years, ranging from 33 to 66. Among them, 16 were female and 6 were male. Three patients had a baseline Eastern Cooperative Oncology Group performance status of 1. Prior to confirmation of dMMR or MSI, three patients with colon cancer had received one cycle of neoadjuvant chemotherapy (capecitabine plus oxaliplatin).

In patients with T4NanyM0 colon cancer (colon cohort) (*n* = 14), 12 had clinical stage T4a, while 2 had clinical stage T4b. In patients with rectal cancer refusing up-front surgery or radiation therapy (rectum cohort) (*n* = 8), all had clinical stage T3. One patient in the rectum cohort had concomitant colon cancer located at the hepatic flexure; however, the characteristics of the colon tumor were not included in the colon cohort analysis as it required T4 stage criteria, as this patient (patient R3) was classified as T2N0 (Table 1).

**Table 1:** Clinical characteristics of patients at baseline

	Overall (*n* = 22)	Colon cohort (*n* = 14)	Rectum cohort (*n* = 8)^a^
**Age**			

Median (IQR)	47 (33–66)	47 (34–66)	42 (26–61)
Range	19–70	26–69	19–70

**Gender**			

Male	15 (68.2%)	12 (85.7%)	3 (37.5%)
Female	7 (31.8%)	2 (14.3%)	5 (62.5%)

**ECOG**			

0	14 (63.6%)	8 (57.1%)	6 (75.0%)
1	8 (36.4%)	6 (42.9%)	2 (25.0%)

**Primary tumor location**			

Ascending colon	2 (9.1%)	2 (14.3%)	–
Hepatic flexure	6 (27.3%)	6 (42.9%)	–
Transverse colon	2 (9.1%)	2 (14.3%)	–
Descending colon	1 (4.5%)	1 (7.1%)	–
Sigmoid colon	3 (13.6%)	3 (21.4%)	–
Upper rectum	0 (0.0%)	–	0 (0.0%)
Middle rectum	3 (13.6%)	–	3 (37.5%)
Lower rectum	5 (22.7%)	–	5 (62.5%)

**Clinical T stage**			

T3	8 (36.4%)	0 (0.0%)	8 (100.0%)
T4a	12 (54.5%)	12 (85.7%)	0 (0.0%)
T4b	2 (9.1%)	2 (14.3%)	0 (0.0%)

**Clinical N stage**			

N negative	3 (13.6%)	2 (14.3%)	1 (12.5%)
N positive	19 (86.4%)	12 (85.7%)	7 (87.5%)

**Histological differentiation**			

Well to moderate differentiated	15 (68.2%)	8 (57.1%)	7 (87.5%)
Poor to un-differentiated	5 (22.7%)	4 (28.6%)	1 (12.5%)
Unable to determine	2 (9.1%)	2 (14.3%)	0 (0.0%)

**Mucinous carcinoma/signet ring cell carcinoma compound**			

Yes	0 (0.0%)	0 (0.0%)	0 (0.0%)
No	22 (100.0%)	14 (100.0%)	8 (100.0%)

**Prior chemotherapy**			

Yes (regimen)	3 (13.6%) (CAPOX)	3 (21.4%) (CAPOX)	0 (0.0%)
No	19 (86.4%)	11 (78.6%)	8 (100.0%)

**Lynch syndrome**			

Yes	8 (36.4%)	5 (35.7%)	3 (37.5%)
No	8 (36.4%)	4 (28.6%)	4 (50.0%)
Unknown	6 (27.3%)	5 (35.7%)	1 (12.5%)

Data are *n* (%) unless otherwise indicated. Percentages might not total 100 because of rounding. IQR, interquartile range; ECOG, Eastern Cooperative Oncology Group; CAPOX, capecitabine plus oxaliplatin.

a One patient with both a rectum and a colon tumor located at hepatic flexure was included in the rectum cohort, and characteristics of the colon tumor were not included in the colon cohort (T4 stage required) due to its T2 stage.

### Primary endpoint

**Figure 2:**
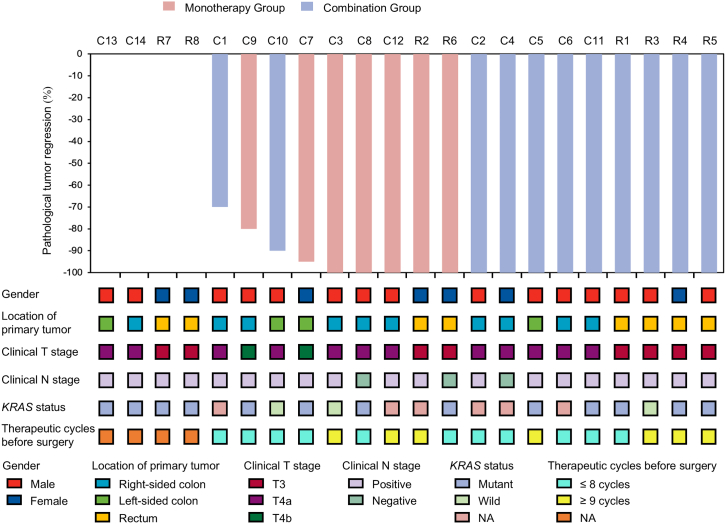
Waterfall plot of pathological tumor regression Patients C13, C14, R7, and R8 had not undergone radical surgery as of the data cutoff date; therefore, pathological tumor regression information was not available for these cases. See also Tables S3 and S4.

As of December 1, 2024 (data cutoff), the median duration of follow-up was 35.6 months (interquartile range [IQR]: 29.9–41.3 months). In the colon cohort, per-protocol set (PPS) comprised 12 patients who underwent radical surgery and completed perioperative treatment. Among them, 8 patients (57.1% [95% confidence interval [CI] 28.9–82.3] in full analysis set [FAS] and 66.7% [34.9–90.1] in PPS) achieved a complete pathological response based on blinded, independent, central review (BICR). Two patients achieved cCR based on endoscopy, computed tomography, MRI, and arcinoembryonic antigen (CEA) level (Table 2; Figure 2). In the rectum cohort, PPS comprised 6 patients who underwent radical surgery, and all of them (75.0% [35.6–95.5] in FAS and 100.0% [51.7–100.0] in PPS) achieved a complete pathological response based on BICR. Two patients achieved a cCR and chose a “watch and wait” approach (Table 2; Figure 2). In both the colon and rectum cohorts, the primary endpoints of the first stage were met, indicating successful outcomes. Based on the Bayesian hierarchical model (Methods S1), the probability of success in this trial was estimated to be more than 99% after continuing enrollment in the second stage.

**Table 2:** Primary endpoint and secondary endpoints of efficacy in both FAS and PPS

Endpoints	FAS (*n* = 22), % (95% CI)		PPS (*n* = 17), % (95% CI)	
	Colon cohort (*n* = 14)	Rectum cohort (*n* = 8)	Colon cohort (*n* = 12)	Rectum cohort (*n* = 6)
**Primary endpoint**				

pCR rate by BICR	57.1 (28.9–82.3)	75.0 (35.6–95.5)	66.7 (34.9–90.1)	100.0 (51.7–100.0)

**Secondary endpoints**				

ORR	78.6 (48.8–94.3)	87.5 (47.3–99.7)	75.0 (42.8–94.5)	83.3 (36.5–99.1)
R0 resection rate	85.7 (57.2–98.2)	75.0 (35.6–95.5)	100.0 (73.5–100.0)	100.0 (51.7–100.0)
pCR rate by local assessment	57.1 (28.9–82.3)	75.0 (35.6–95.5)	66.7 (34.9–90.1)	100.0 (51.7–100.0)
pCR rate by both local assessment and BICR	57.1 (28.9–82.3)	75.0 (35.6–95.5)	66.7 (34.9–90.1)	100.0 (51.7–100.0)

**TRG**				

Grade 0	57.1 (28.9–82.3)	75.0 (35.6–95.5)	66.7 (34.9–90.1)	100.0 (51.7–100.0)
Grade 1	14.3 (1.8–42.8)	0.0 (0.0–36.9)	16.7 (2.1–48.4)	0.0 (0.0–45.9)
Grade 2	7.1 (0.2–33.9)	0.0 (0.0–36.9)	8.3 (0.2–38.5)	0.0 (0.0–45.9)
Grade 3	7.1 (0.2–33.9)	0.0 (0.0–36.9)	8.3 (0.2–38.5)	0.0 (0.0–45.9)
1-year event-free survival rate	100%	88% (64%–100%)	100%	100%
2-year event-free survival rate	100%	88% (64%–100%)	100%	100%
1-year disease-free survival rate	not applicable	not applicable	100%	100%
2-year disease-free survival rate	not applicable	not applicable	100%	100%
1-year overall survival rate	100%	100%	100%	100%
2-year overall survival rate	100%	100%	100%	100%

FAS, full analysis set; PPS, per-protocol set; pCR, pathological complete response; BICR, blinded, independent, central review; ORR, objective response rate; TRG, tumor regression grade.

See also Figure S1; Tables S3 and S4.

### Secondary endpoints

**Figure 3:**
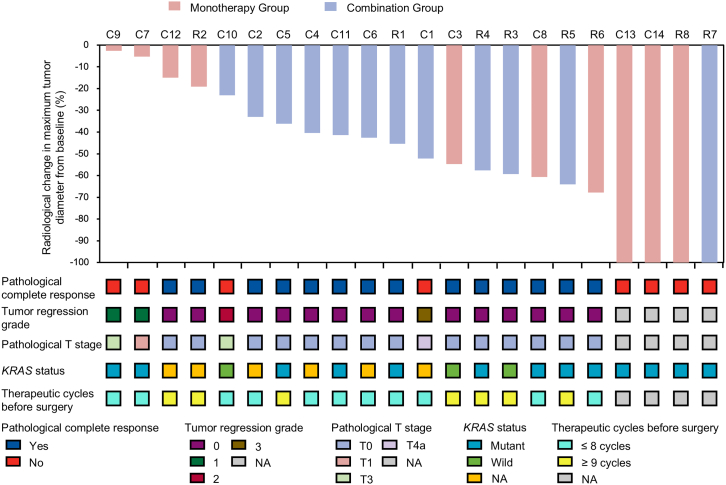
Waterfall plot of radiological change in maximum tumor diameter from baseline according to Response Evaluation Criteria In Solid Tumors v.1.1 Patients C13, C14, R7, and R8 had not received radical surgery by the data cutoff. See also Tables S3 and S4.

In the FAS, the objective response rate (ORR), R0 resection rates, pCR rate assessed by local investigator, and pCR rate assessed by both BICR and local investigator were 78.6% (95% CI 48.8–94.3) and 87.5% (47.3–99.7), 85.7% (57.2–98.2) and 75.0% (35.6–95.5), 57.1% (28.9–82.3) and 75.0% (35.6–95.5), and 57.1% (28.9–82.3) and 75.0% (35.6–95.5), respectively, in the colon cohort and rectum cohort (Figure 3; Table 2). The summary of these endpoints in the PPS was presented in Table 2. In the PPS, the median time to surgery was 3.5 months (IQR 2.5–5.6 months) for the colon cohort and 6.0 months (IQR 4.4–7.3 months) for the rectum cohort. Survival endpoints were summarized in Table 2. In patients receiving radical surgery, no one experienced relapse or progression. In the 4 patients with cCR, only one had reginal lymph node progression (Table 3). We were unable to collect the data of life quality at every assessment time point due to COVID-19 epidemic. Individual patient data are summarized in Table 3.

**Table 3:** Individual patient data

	Gender	Age (year)	ECOG	Lynch syndrome	Clinical T and N stage	Primary site	Treatment regimen^b^	*KRAS*	*NRAS*	*BRAF*	Surgery	Cycles of TORI		TTS (month)	Pathological stage	TRG	Follow-up (month)
												Pre-O	Post-O				
C1	male	66	1	unknown	T4aN+	hepatic flexure	TORI+IRI+BEV	unknown	unknown	unknown	yes	3	9	2.6	ypT4aN0	3	33.5^c^
C2	male	50	1	unknown	T4aN+	ascending colon	TORI+IRI+BEV	unknown	unknown	unknown	yes	4	2	2.5	ypT0N0	0	32.3^c^
C3	male	31	0	yes	T4aN+	transverse colon	TORI	wild	wild	wild	yes	9	0	5.6	ypT0N0	0	26.3^c^
C4	female	57	0	unknown	T4aN−	ascending colon	TORI+IRI+BEV	unknown	unknown	unknown	yes	3	6	3.1	ypT0N0	0	27.7^c^
C5	male	67	1	unknown	T4aN+	sigmoid colon	TORI+IRI+BEV	mutant	wild	wild	yes	9	0	6.0	ypT0N0	0	27.0^c^
C6	male	41	0	unknown	T4aN+	hepatic flexure	TORI+IRI+BEV	unknown	unknown	unknown	yes	3	9	2.0	ypT0N0	0	25.7^c^
C7	female	33	0	no^a^	T4bN+	sigmoid colon	TORI	mutant	wild	wild	yes	6	6	3.5	ypT1N1	1	24.0^c^
C8	male	34	0	no	T4aN−	hepatic flexure	TORI	mutant	wild	wild	yes	4	0	2.1	ypT0N0	0	20.5^c^
C9	male	69	0	no	T4bN+	hepatic flexure	TORI	mutant	wild	wild	yes	3	9	1.8	ypT3N1a	1	22.6^c^
C10	male	43	0	no	T4aN+	descending colon	TORI+IRI	wild	wild	wild	yes	3	7	1.8	ypT3N0	2	21.6^c^
C11	male	50	1	yes	T4aN+	hepatic flexure	TORI+IRI+BEV	mutant	wild	wild	yes	5	7	2.8	ypT0N0	0	16.0^c^
C12	male	26	1	yes	T4aN+	hepatic flexure	TORI	unknown	unknown	unknown	yes	9	0	3.9	ypT0N0	0	11.1^c^
C13	male	69	0	yes	T4aN+	sigmoid colon	TORI	mutant	wild	wild	no	12	NA	NA	NA	NA	8.0^d^
C14	male	34	1	yes	T4aN+	transverse colon	TORI	mutant	wild	wild	no	12	NA	NA	NA	NA	7.6^d^
R1	male	19	0	yes	T3N+	lower rectum	TORI+IRI+BEV	mutant	wild	wild	yes	7	5	4.0	ypT0N0	0	26.5^c^
R2	female	26	0	unknown	T3N+	middle rectum	TORI	unknown	unknown	unknown	yes	9	3	4.4	ypT0N0	0	24.2^c^
R3	male	70	0	no	T3N+	lower rectum	TORI+IRI+BEV	wild	wild	mutant	yes	9	0	7.3	ypT0N0	0	18.2^c^
R4	female	26	0	no	T3N+	lower rectum	TORI+IRI+BEV	mutant	wild	wild	no	12	0	9.7	ypT0N0	0	17.6^c^
R5	male	51	1	yes	T3N+	middle rectum	TORI+IRI	mutant	wild	wild	yes	10	0	6.4	ypT0N0	0	17.1^c^
R6	female	52	0	no	T3N−	middle rectum	TORI	mutant	wild	wild	yes	8	0	5.5	ypT0N0	0	14.3^c^
R7	female	33	0	no	T3N+	lower rectum	TORI+IRI+BEV	mutant	wild	wild	no	12	NA	NA	NA	NA	12.4^e^
R8	female	70	1	yes	T3N+	lower rectum	TORI	mutant	wild	wild	no	12	NA	NA	NA	NA	8.8^d^

TORI, toripalimab; IRI, irinotecan; BEV, bevacizumab; NA, not applicable; Pre-O, pre-operative; Post-O, post-operative; TRG, tumor regression grade.

See also Figure S1; Tables S3 and S4.

a Patient C7 was classified as having a Lynch-like syndrome, as he fulfilled the Amsterdam criteria but had no detectable germline mutations in *MLH1*, *MSH2*, *MSH6*, *PMS2*, or *EPCAM*.

b If surgery was planned, patients discontinued bevacizumab while continuing other medications. Surgery was scheduled no earlier than 6 weeks after the last dose of bevacizumab and 2–4 weeks after the last dose of toripalimab or irinotecan.

c These patients were disease-free at the last follow-up.

d Patients C13, C14, and R8 achieved clinical complete response (cCR) after 6-month treatment and chose watch and wait strategy. They had no evidence of disease recurrence at the last follow-up.

e Patient R7 achieved cCR after 6-month treatment and chose watch and wait strategy. However, she had regional lymph node progression and increasing CEA level 3 months later. She refused surgery and responded to rechallenge with anti-PD-1 blockade.

### Safety endpoint

Treatment-related adverse events (TRAEs) were summarized in Table S2. Sixteen patients (72.7%) experienced at least one TRAE of drugs during the study. Based on a shared decision-making strategy between doctors and patients, 12 patients received toripalimab in combination with irinotecan and bevacizumab (combination group), while 10 patients received toripalimab monotherapy (monotherapy group) in the neoadjuvant setting. Grade 3 and 4 TRAEs of drugs were only observed in the combination group, including neutropenia (25%), leukopenia (8.3%), and anemia (8.3%), and were not observed in the monotherapy group. Surgery-related adverse events were observed in two patients (2/18, 11.1%). Patient C10 experienced grade 4 intraoperative bleeding and grade 2 pancreatic fistula, and patient R1 developed grade 3 anastomotic fistula.

### Exploratory analysis

The mutation status of *KRAS*/*NRAS*/*BRAF* was available for 16 patients. Among the 13 patients with *KRAS* mutation and *NRAS*/*BRAF* wild type, 9 underwent surgery, and 7 achieved pCR. One patient with the *BRAF* p.R239Q mutation and *KRAS*/*NRAS* wild type also achieved pCR. Among the 2 patients with *KRAS*/*NRAS*/*BRAF* wild type, one achieved pCR, while the other did not.

Although the rates of pCR (81.8%) and tumor regression grade (TRG) 0–1 (81.8%) in the combination group (*n* = 11) were comparable to that in the monotherapy group (71.4% and 100%, respectively, *n* = 7), ORR seemed higher in the combination group (91.7% vs. 60.0% in the monotherapy group) (Table S3). The median number of therapeutic cycles was 8.5. Patients who received ≥9 therapeutic cycles exhibited a higher pCR rate (100%) compared to those who received ≤8 cycles (63.6%). However, the rates of TRG 0–1 and ORR did not show a marked increase with additional cycles (Table S4).

## Discussion

Given that other neoadjuvant immunotherapy studies have not incorporated chemotherapy, this study investigated the efficacy and safety of combining PD-1 antibody with chemotherapy and vascular endothelial growth factor (VEGF) antibody in locally advanced dMMR/MSI CRC.^20^ The results demonstrated that the combination of toripalimab, irinotecan, and bevacizumab showed satisfactory activity and tolerability. A total of 22 patients were enrolled in the first stage of the study. In the Colon Cohort (*n* = 14), 12 patients underwent radical surgery, and 8 achieved a pCR. In the Rectum Cohort (*n* = 8), 6 patients underwent radical surgery, and all of them achieved pCR. Therefore, the primary endpoint was met in both the Colon and Rectum Cohorts.

T4 stage was considered an advanced stage and was associated with a poorer prognosis in CRC.^13^ In dMMR/MSI T2–4 gastric cancers treated with durvalumab plus tremelimumab, the pCR rate was 17% (1/6) in T4 tumors, significantly lower than the rate of 89% (8/9) in T2–3 tumors (*p* = 0.011).^25^ It was unknown whether T4 stage is associated with a poor response to ICIs in CRC. Therefore, we focused on T4CC in this trial. All tumors showed shrinkage with an ORR of 78.6%. Among the 12 patients who underwent radical surgery, 8 achieved pCR. The pCR rate was comparable to the rates of 57.1% (4/7) from nivolumab with ipilimumab^10^ and 76.9% (20/26) from toripalimab with or without celecoxib in resected T4 tumors.^11^ Another retrospective study reported a pCR rate of 59.5% (22/38) in dMMR/MSI T4 CRC treated with PD-1 blockade-based therapy.^12^ Taken together, these results demonstrate high anti-tumor efficacy and a promising pCR rate, even in T4CC. Importantly, even considering the high likelihood of recurrence after standard adjuvant chemotherapy,^26^ dMMR T4CC appeared unlikely to relapse after neoadjuvant ICIs based on our results and those of other studies.^2^^,^^12^ This emphasizes the necessity of ICIs before radical surgery in such patients. Based on the inspiring cCR rate in LARC treated with anti-PD-1 antibody,^19^ the last two enrolled patients, C13 and C14, refused surgery and received 6-month treatment. They achieved cCR, chose to watch and wait, and had no disease recurrence after 22.3 and 21.9 months, respectively, which was consistent with the previous study.^27^

For LARC, the standard therapy is total neoadjuvant treatment, which includes chemoradiotherapy and induction or consolidation chemotherapy. However, considering the adverse events and unclear benefit of this approach in dMMR/MSI patients, two clinical trials were conducted and provided high pCR or cCR rates after anti-PD-1 antibody.^18^^,^^19^ In our trial, among the 8 enrolled patients, 6 who underwent surgery achieved pCR, and 2 achieved cCR after 6 months of treatment. These results further support the use of neoadjuvant ICIs as a promising therapy for dMMR/MSI LARC. However, one patient with cCR experienced progression in reginal lymph node and no regrowth in primary tumor after stopping treatment. Though she responded to rechallenge with anti-PD-1 antibody and finally achieved cCR again, the optimal strategy for these patients remained to be explored.

As mentioned earlier, neoadjuvant ICI-based treatment has shown promising results in dMMR/MSI locally advanced CRC. To further advance this approach, it is important to investigate the preferred treatment regimen and treatment duration, as previous reports have provided limited insights into these aspects.

When selecting treatment for dMMR/MSI locally advanced CRC, it is important to consider both avoiding early progression and increasing efficacy. Fortunately, early progression is rare, and distal metastases had not been observed when treated with anti-PD-1 alone.^11^^,^^12^^,^^18^^,^^19^^,^^28^^,^^29^^,^^30^ Additionally, these patients seemed unlikely to relapse after timely salvage surgery.^12^^,^^18^ It should be noted that primary resistance mechanisms discovered in metastatic disease may not apply to locally advanced stages. A relevant example is the presence of *KRAS* mutations. In the first-line setting, pembrolizumab did not demonstrate longer progression-free survival compared to chemotherapy in *KRAS*-mutant patients.^7^ In the late-line setting, the ORR was lower in patients with *KRAS* mutations (33%) than in the *RAS*/*BRAF* wild-type population (45%) receiving nivolumab.^3^ However, among the 13 patients with *KRAS* mutations in our study, none experienced tumor growth, and the pCR rate in patients undergoing surgery was 75% (6/8), which was comparable to the overall population and consistent with findings in the NICHE-2 study.^31^ Additionally, *KRAS* mutation status showed no association with tumor response and TRG.^12^ An intriguing observation in this study was the *KRAS* mutation rate of 81.3%, which was numerically higher than those reported in other studies. In the Asian population, *KRAS* mutation rates have been documented to range between 48.9% and 56.6%.^32^^,^^33^^,^^34^ By contrast, some large sample studies predominantly enrolling European and American patients, have reported *RAS* mutation rates of approximately 32.7%–35.6%.^7^^,^^35^^,^^36^ In addition, previous studies have demonstrated that patients with Lynch syndrome or Lynch-like syndrome exhibit a higher prevalence of *KRAS* mutations compared to those with sporadic tumors.^37^^,^^38^ In this present study, germline testing was performed on 16 patients, revealing 8 cases of Lynch syndrome (7 of which harbored *KRAS* mutations, 87.5%), 1 case of Lynch-like syndrome (carrying a *KRAS* mutation), and 7 cases of sporadic CRC (5 with *KRAS* mutations, 71.4%). Therefore, the higher proportion of *KRAS* mutations observed in this study may be attributed to ethnic variations, a relatively higher prevalence of Lynch syndrome or Lynch-like syndrome, and sampling errors. Furthermore, the disparities in *KRAS* mutation rates between Asian populations and those in Europe or America could reflect underlying genetic differences among these populations, underscoring the need for cautious interpretation when extrapolating our findings to other populations.

Combining chemotherapy with PD-1 blockade has been shown to potentially increase efficacy or overcome resistance in dMMR/MSI tumors in the palliative setting.^39^^,^^40^ However, there is currently no data on the combination of ICIs with chemotherapy and anti-VEGF treatment in the neoadjuvant setting. In our exploratory analysis, although the pCR rates of the two groups were similar, the ORR of the combination group was much higher. This suggests that PD-1 antibody in combination with chemotherapy and bevacizumab may be more suitable for patients who need to minimize the tumor volume and reduce the surgical scope to the greatest extent, which is consistent with our findings in the palliative setting.^40^ Furthermore, the addition of celecoxib, a COX-2 inhibitor intended to modulate the tumor microenvironment, did not significantly improve the efficacy of the PD-1 antibody.^11^ When used for a relatively short duration, the addition of anti-CTLA-4 antibodies can enhance efficacy in the neoadjuvant setting.^41^ However, the differences in pCR rates between PD-1 antibody monotherapy and PD-1 and CTLA-4 dual blockade might be lesser if more ICI doses were delivered. It should be noticed that CTLA-4 antibody may not be necessary for patients with Lynch syndrome.^36^^,^^41^ However, it is worth noting that the proportion of Lynch syndrome in the real world may be lower than that reported in published clinical trials, reflecting a biased referral pattern of clinical trial centers.^42^

Duration of treatment appeared to be an important factor associated with pCR in this study, as 100% of patients with therapeutic cycles ≥9 achieved pCR compared to 63.6% in those with therapeutic cycles ≤8. Additionally, four patients with 12 cycles of therapy achieved cCR. In another trial, only one out of three patients achieved pCR after 3 months of sintilimab treatment, while 11 out of 12 patients who received 6 or more months of treatment achieved cCR or pCR.^18^ In the trial conducted by Cercek et al., all tumors were assessed as cCR at 6 months but not at 3 months^19^ Based on the current evidence, it is reasonable to assume that 6 months of PD-1 antibody treatment may provide maximum benefit, but this hypothesis should be further validated in prospective clinical trials.

Although the short-term efficacy of ICIs in dMMR/MSI LACRC has been established several years ago, the long-term outcomes were much more important.^20^ In this study with a median follow-up of 35.6 months, none of the 18 patients with resected tumors relapsed. Four patients chose watch and wait after cCR, and only one had lymph node progression but responded to rechallenge of PD-1 antibody and finally achieved cCR again. These results were in accordance with our previous findings and those of others.^43^^,^^44^^,^^45^ The outstanding long-term efficacy of ICIs might change the clinical practice of dMMR/MSI LACRC.

In conclusion, this trial reported the efficacy and safety of toripalimab, with or without irinotecan and bevacizumab, in both dMMR/MSI T4CC and LARC. The results demonstrated a satisfactory pCR rate, and no patients experienced disease progression during the neoadjuvant treatment. After a long follow-up, no patients with resected tumors suffered from disease recurrence. Furthermore, no unexpected TRAEs or grade 4 or 5 TRAEs were observed. The higher pCR rate observed in patients receiving ≥9 cycles is encouraging, and we are currently designing a randomized trial to validate these findings.

### Limitations of the study

This study had some limitations. First, the sample size was small. Second, the exploratory analysis concerning efficacy differences across various treatment regimens (monotherapy vs. combination) and therapeutic cycles (≥9 vs. ≤8) was not included in the original study design. Therefore, we could not conclude whether or not irinotecan and bevacizumab or longer treatment duration contributed to a higher pCR rate. This topic of interest warrants further investigation in future studies. Third, the *KRAS* mutation rate observed in our study was 81.3%, which is higher than that reported in other American or European studies. This might suggest a potential difference in genetic background, and thus, the conclusions should be cautiously extrapolated to other populations.

## Resource availability

### Lead contact

Further information and requests for resources and reagents should be directed to and will be fulfilled by the lead contact, Lin Shen (shenlin@bjmu.edu.cn).

### Materials availability

This study did not generate new unique reagents.

### Data and code availability

• All data reported in this paper and any additional information required to reanalyze the data will be shared by the lead contact upon reasonable request. Specifically, de-identified individual patient-level data such as baseline clinical variables and grade of adverse events for each participant will be available upon request. Any additional information regarding individual participants that may result in breach of patient confidentiality will not be provided.
• This publication does not generate new code.
• Any additional information required to reanalyze the data reported in this work paper is available from the lead contact upon request.

## Acknowledgments

We thank Junshi Biosciences for providing JS001 for free and assisting in conducting this trial. We thank Yuxiao Tong for helping translating the protocol from Chinese to English. This study was funded by National Natural Science Foundation of China (nos. 82403443 and 91959130), Beijing Xisike Clinical Oncology Research Foundation (nos. Y-HH202101-0068 and Y-tongshu2021/ms-0040), and Beijing Hospitals Authority Clinical Medicine Development of Special Funding Support (no. ZLRK202327). This study was supported by Junshi Biosciences. The funders had no role in the design and conduct of the study; collection, management, analysis, and interpretation of the data; preparation, review, or approval of the manuscript; and decision to submit the manuscript for publication.

## Author contributions

L.S. and J. Li. contributed to the conception and design of the study. All authors contributed to the acquisition, analysis, or interpretation of data. Z.W., X.W., X.Z., J. Leng., T.X., and M. Chen. wrote the drafting of the manuscript. Z.W., X.W., and J. Li. contributed to the critical revision of the manuscript for important intellectual content. Z.W., T.X., and M. Chen. performed the statistical analysis. All authors have read and agreed to the published version of the manuscript.

## Declaration of interests

The authors declare no competing interests.

## STAR★Methods

### Key resources table

**Table:** 

REAGENT or RESOURCE	SOURCE	IDENTIFIER
**Biological samples**		

Formalin-fixed paraffin-embedded archival tumor specimens	This manuscript	N/A

**Chemicals, peptides, and recombinant proteins**		

Toripalimab/JS001	Junshi Biosciences	www.junshipharma.com
Irinotecan	Pfizer	www.pfizer.com
Bevacizumab	Roche	www.roche.com

**Deposited data**		

Patient data	This manuscript	N/A

**Software and algorithms**		

SPSS software (version 23)	IBM	www.ibm.com
R version 4.3.0	The R Foundation	www.r-project.org

### Experimental model and study participant details

Chinese adults, both male and female, with histologically confirmed resectable rectal cancer who refused up-front surgery or radiation therapy (rectum cohort) or T4NanyM0 colon cancer (colon cohort) with deficiency of mismatch repair (dMMR) or microsatellite instability (MSI) were enrolled in the study. Demographic information was provided in Table 1, and no significant association of gender with the results of the study was found. All patients provided written informed consent prior to enrollment.

The study was conducted in accordance with the principles of the Declaration of Helsinki and the International Conference on Harmonization and Good Clinical Practice Guidelines. Each center had independent ethics committee that granted approval for the research protocol (Ethics Approval Number: 2019YJZ66 in Beijing Cancer Hospital, and 21K085-002 in The First Hospital of Jilin University).

### Method details

#### Patient eligibility

Patients were eligible for enrollment if they had histologically confirmed colorectal cancer (CRC) with local confirmation of dMMR or MSI status, and met one of the following criteria: a) resectable rectal cancer classified as T3-4 or T1-2 with the refusal of up-front surgery or radiation therapy (rectum cohort), or b) resectable colon cancer classified as T4a-b (colon cohort). Key inclusion criteria included an age range of 18–75 years, adequate hematological, hepatic, and renal function, measurable disease as per the Response Evaluation Criteria In Solid Tumors (RECIST) v1.1 criteria, and no prior local treatment. Key exclusion criteria included prior blockade of PD-1/PD-L1/PD-L2 or CTLA-4 and any contraindication to toripalimab, irinotecan and bevacizumab. The full list of inclusion and exclusion criteria is provided in the protocol (Methods S1).

#### Subject allocation

This current phase Ib/II clinical trial is a single arm study with no control group. All patients are planned to receive toripalimab plus irinotecan and bevacizumab followed by radical surgery. However, this trial allows a doctor-patient shared-decision making process to determine whether to omit irinotecan or bevacizumab and the optimal surgery timing.

#### Study design

This dual-institution, open-label, phase Ib/II trial was conducted at two hospitals in China, namely Beijing Cancer Hospital and The First Hospital of Jilin University. Enrolled patients received toripalimab (3mg/kg d1), irinotecan (180mg/m2 d1) and bevacizumab (5mg/kg d1) every two weeks for three cycles, followed by radical surgery and adjuvant toripalimab at a dose of 240mg every 3 weeks. The entire perioperative treatment duration did not exceed six months. Based on the most recent findings from ongoing trials such as PICC and the study by Cercek et al. (11, 19), a multidisciplinary team (MDT)-based doctor-patient shared decision-making process was implemented in this trial. MDT members would inform patients about the latest advancements in the neoadjuvant immunotherapy field, and then discuss with them whether to omit irinotecan or bevacizumab at baseline and determine whether to repeat another three cycles of therapy or proceed to radical surgery at each tumor assessment timepoint until surgery was performed. If surgery was planned, patients discontinued bevacizumab while continuing other medications. Surgery was scheduled no earlier than 6 weeks after the last dose of bevacizumab and 2–4 weeks after the last dose of toripalimab or irinotecan. This process also guided the decision of whether to proceed with radical surgery after the initial three cycles or to continue neoadjuvant treatment.

Radiological assessments of the tumor were conducted using thoracoabdominopelvic CT scans at baseline and after every three cycles of treatment. Patients in the Rectum Cohort underwent additional pelvic MRI. Clinical staging and changes in the maximum tumor diameter were evaluated by a senior radiologist (X.Z.). The extent of viable tumor remaining in the resected primary tumors (pathological regression) was assessed by a senior gastrointestinal pathologist (Y.S.).

Adverse events were monitored from the initiation of treatment until 90 days after the last dose of toripalimab or 30 days after surgery. The severity of adverse events was graded according to the Common Terminology Criteria for Adverse Events (CTCAE; version 5.0).

Following surgery, patients underwent physical examinations and measurement of carcinoembryonic antigen every three months for the first three years and every six months from years 4–5. Thoracoabdominopelvic CT scans for imaging surveillance were performed every six months during years 1–3 and every 12 months during years 4–5. A total colonoscopy was required within the first year, and a mandatory colonoscopy was conducted three years postoperatively.

#### Outcomes

The primary endpoint of this study was the pCR rate in all patients who received neoadjuvant therapy, as determined by a blinded, independent, central review (BICR). Secondary endpoints included the R0 resection rate, time to surgery (TTS), pCR rate assessed by the local investigator, pCR rate based on both BICR and the local investigator, tumor regression grade (TRG), objective response rate (ORR) based on RECIST 1.1 criteria, event-free survival (time from study treatment to the first documented inoperable disease progression, local or distant recurrence, or death), disease-free survival (time from the date of surgery to disease relapse or death), one-year and two-year disease-free survival rates (percentage of patients achieving disease-free survival for more than one and two years, respectively, from the date of surgery), one-year and two-year overall survival rates (percentage of patients surviving for more than one and two years, respectively, from the date of the first dose), and assessment of life quality (using the EORTC QLQ-C30 and EORTC QLQ-CR29 scales). Safety endpoints included incidence of treatment-related adverse events (TRAEs) associated with drugs and surgery.

#### Exploratory analysis

We retrospectively collected the mutation status of *KRAS*, *NRAS*, and *BRAF* from patients who underwent local testing, regardless of the testing platform and panel used. We assessed the pathological response and radiological response based on different gene statuses (wild-type vs. mutation), treatment regimens (toripalimab alone vs. toripalimab ± irinotecan ± bevacizumab) and therapeutic cycles (≤8 vs. ≥ 9).

### Quantification and statistical analysis

Sample size calculations were performed using a Bayesian hierarchical model^46^ and were determined through a large-scale simulation study. The assumed pCR rate was 35% in the Rectum Cohort and 10% in the historical control group. In the Colon Cohort, the pCR rate was assumed to be 15%, while the historical control group had a rate of 3%. The Type I error rate was set at 5%, and the Type II error rate (statistical power) was set at 20% (i.e., achieving 80% power).

Enrollment was conducted in two stages to determine the required number of patients. In the first stage, 11 patients with colon cancer and 8 patients with rectal cancer were enrolled. If more than 2 out of the 11 patients in the Colon Cohort and more than 3 out of the 8 patients in the Rectum Cohort achieved pCR in the first stage, an additional 17 colon cancer patients and 8 rectal cancer patients would be enrolled in the second stage. Efficacy Endpoints in the Colon Cohort and Rectum Cohort were analyzed separately and summarized in full analysis set (FAS) (all patients who signed the informed consent form, met the inclusion criteria, and did not meet any exclusion criteria) and per-protocol set (PPS) (patients who signed the informed consent form, had no major protocol deviations, and had valid baseline data and primary endpoint measurements). Safety endpoints were analyzed in safety set (SS) (patients who received at least one dose of toripalimab) (Methods S1).

The pCR rate, ORR, R0 resection rate, and rates of different tumor regression grade (TRG) were summarized as frequencies and proportions. Two-sided 95% confidence intervals (CI) were calculated using the Clopper-Pearson method. Safety data were presented as the frequency and proportion of patients experiencing each event. Kaplan-Meier analysis was used to estimate event-free survival, disease-free survival, and overall survival, along with corresponding 95% CIs. The reverse Kaplan-Meier method was employed to calculate the median follow-up time and corresponding interquartile range (IQR). All statistical analyses were performed using SPSS software (version 23) and R software (version 4.3.0).

### Additional resources

This trial was the retrospectively registered at ClinicalTrials.gov as NCT04988191 on July 31, 2021. Prior to registration, a total of six patients were enrolled in the study, including five with colon cancer and one with rectal cancer, and other 16 patients were enrolled after registration (Table S1).

## Supplemental information

Document S1. Figures S1, Tables S1–S6, and Method S1

Document S2. Article plus supplemental information
