# Supplementary material for: Toripalimab, bevacizumab, and irinotecan in dMMR/MSI locally advanced colorectal cancer: First-stage results from a phase 1b/2 trial
Source: Cell Rep Med. 2025 Aug 15;6(9):102296. doi: 10.1016/j.xcrm.2025.102296 (PMC12490223; doi:10.1016/j.xcrm.2025.102296)
Supplement: Document S2. Article plus supplemental information [file mmc2.pdf]

Cell Reports
Medicine

Article

Toripalimab, bevacizumab, and irinotecan in dMMR/MSI locally advanced colorectal cancer: First-stage results from a phase 1b/2 trial

Graphical abstract

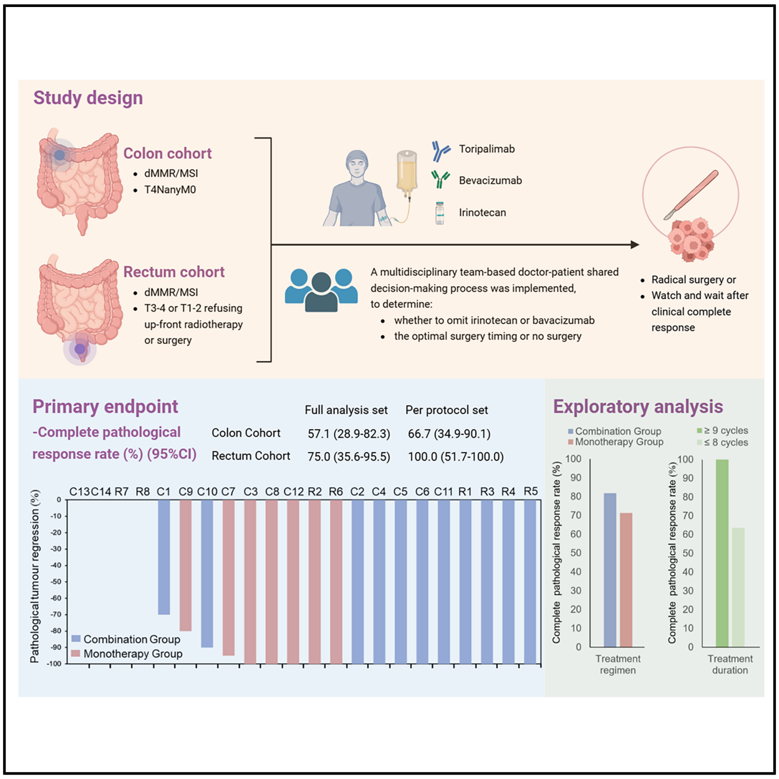

Highlights

- Toripalimab plus irinotecan and bevacizumab has a promising pCR rate
- Toxicity is manageable, and long-term survival benefit is observed
- More doses of toripalimab are associated with a higher pCR rate
- Irinotecan and bevacizumab appear to increase objective response rate but not pCR rate

Authors

Zhenghang Wang, Xicheng Wang, Xiaoyan Zhang, ..., Mifen Chen, Jian Li, Lin Shen

Correspondence

oncogene@163.com (J.L.), shenlin@bjmu.edu.cn (L.S.)

In brief

Wang et al. show that toripalimab plus irinotecan and bevacizumab is a safe and active regimen in patients with T4NanyM0 colon cancer or locally advanced rectal cancer with deficiency of mismatch repair or microsatellite instability. The potential contribution of chemotherapy and anti-VEGF treatment to PD-1 antibody should be further evaluated.

Wang et al., 2025, Cell Reports Medicine *6*, 102296
September 16, 2025 © 2025 The Author(s). Published by Elsevier Inc.
https://doi.org/10.1016/j.xcrm.2025.102296

Cell Reports Medicine

CellPress
OPEN ACCESS

Article

Toripalimab, bevacizumab, and irinotecan in dMMR/MSI locally advanced colorectal cancer: First-stage results from a phase 1b/2 trial

Zhenghang Wang,[2,10] Xicheng Wang,[2,10] Xiaoyan Zhang,[3,10] Jiahua Leng,[4,10] Ming Cui,[5] Ji Zhang,[6] Quan Wang,[7] Yu Sun,[8,9] Ting Xu,[1] Mifen Chen,[1] Jian Li,[1,2,*] and Lin Shen[1,2,11,*]

[1]State Key Laboratory of Holistic Integrative Management of Gastrointestinal Cancers, Beijing Key Laboratory of Carcinogenesis and Translational Research, Department of Gastrointestinal Oncology, Peking University Cancer Hospital & Institute, Beijing 100142, China

[2]Key Laboratory of Carcinogenesis and Translational Research (Ministry of Education/Beijing), Department of Gastrointestinal Oncology, Peking University Cancer Hospital & Institute, Beijing 100142, China

[3]Key Laboratory of Carcinogenesis and Translational Research (Ministry of Education/Beijing), Department of Radiology, Peking University Cancer Hospital & Institute, Beijing 100142, China

[4]Key Laboratory of Carcinogenesis and Translational Research (Ministry of Education/Beijing), Gastrointestinal Cancer Center, Unit III, Peking University Cancer Hospital & Institute, Beijing 100142, China

[5]Key Laboratory of Carcinogenesis and Translational Research (Ministry of Education/Beijing), Gastrointestinal Cancer Center, Unit IV, Peking University Cancer Hospital & Institute, Beijing 100142, China

[6]Key Laboratory of Carcinogenesis and Translational Research (Ministry of Education/Beijing), Gastrointestinal Cancer Center, Unit II, Peking University Cancer Hospital & Institute, Beijing 100142, China

[7]The First Hospital of Jilin University, Department of Gastrointestinal Surgery, Chang Chun 130031, China

[8]State Key Laboratory of Holistic Integrative Management of Gastrointestinal Cancers, Beijing Key Laboratory of Carcinogenesis and Translational Research, Department of Pathology, Peking University Cancer Hospital & Institute, Beijing 100142, China

[9]Key Laboratory of Carcinogenesis and Translational Research (Ministry of Education/Beijing), Department of Pathology, Peking University Cancer Hospital & Institute, Beijing 100142, China

[10]These authors contributed equally

[11]Lead contact

*Correspondence: oncogene@163.com (J.L.), shenlin@bjmu.edu.cn (L.S.)

https://doi.org/10.1016/j.xcrm.2025.102296

SUMMARY

This is the first stage of the phase 1b/2 trial evaluating the effectiveness and safety of toripalimab, irinotecan, and bevacizumab in patients with rectal cancer refusing up-front surgery or radiation therapy (rectum cohort) and patients with T4NanyM0 colon cancer (colon cohort) with deficiency of mismatch repair (dMMR) or microsatellite instability (MSI). This trial allows a doctor-patient shared decision-making process to determine whether to omit irinotecan or bevacizumab and the optimal surgery timing. The primary endpoint pathological complete response (pCR) rates in the full analysis set (FAS) and per-protocol set (PPS) are 57.1% (95% confidence interval [CI] 28.9–82.3) and 66.7% (34.9–90.1), respectively, in the colon cohort (n = 14) and 75.0% (35.6–95.5) and 100% (51.7–100.0), respectively, in the rectum cohort (n = 8). No disease recurrence occurs in PPS. No grade 4–5 drug-related adverse events are observed. Toripalimab with or without irinotecan and bevacizumab shows promising efficacy and manageable toxicity in dMMR/MSI T4NanyM0 colon cancer and locally advanced rectal cancer (ClinicalTrials.gov: NCT04988191).

INTRODUCTION

Colorectal cancer (CRC) ranks as the third most commonly diagnosed cancer and the second leading cause of cancer-related mortality worldwide.[1] In the context of metastatic CRCs characterized by the deficiency of mismatch repair (dMMR) or microsatellite instability (MSI), immune checkpoint inhibitors (ICIs) such as programmed death 1 (PD-1) or programmed death-ligand 1 (PD-L1) antibodies, either alone or in combination with cytotoxic T lymphocyte associated protein 4 (CTLA-4) antibodies, have demonstrated remarkable efficacy in late-line treatment across a growing number of clinical trials.[2–5] More recently, pembrolizumab and atezolizumab have exhibited superior outcomes in terms of progression-free survival and overall survival compared to standard treatments in first-line and second-line settings, respectively.[6,7]

The compelling results obtained thus far have led to the exploration of ICI use in the preoperative setting for locally advanced CRC with dMMR/MSI. In the case of colon cancer, dMMR/MSI is present in 18.9%–21.3% of stage II tumors and 14.3%–14.4% of

© 2025 The Author(s). Published by Elsevier Inc.
This is an open access article under the CC BY license (http://creativecommons.org/licenses/by/4.0/).

stage III tumors.[8,9] In NICHE and NICHE-2 study, pathological complete response (pCR) was observed in 60% (12/20) and 67% (72/107), respectively, of patients with stage I–III dMMR colon cancers who were treated with two doses of nivolumab and one dose of ipilimumab.[2,10] In the PICC study, which included 30 dMMR colon tumors, 23 patients (77%) achieved pCR following 3 months of treatment with toripalimab, with or without celecoxib.[11] Notably, no tumor progression was observed during the neoadjuvant treatment in these trials. Although long-term follow-up data are limited, it appears that preoperative ICIs may also extend disease-free survival.[2,12] These encouraging findings demonstrate the potential of ICIs in dMMR colon tumors, particularly those at the T4 stage, which is associated with a poor prognosis[13] and exhibits limited response to neoadjuvant chemotherapy.[14]

In the case of rectal cancer, only a small proportion of patients (4%–10%) are identified as having dMMR/MSI.[15,16] Current standard practice involves neoadjuvant chemotherapy and radiotherapy for resectable T3–4 rectal cancers, as well as for T1–2 rectal cancers unfit for surgery. However, dMMR/MSI locally advanced rectal cancer (LARC) may exhibit progression during neoadjuvant treatment or relapse following surgery subsequent to neoadjuvant chemotherapy or chemoradiotherapy.[17] Furthermore, patients may be subjected to ostomy and experience bowel, urinary, or sexual dysfunction as a result of perioperative treatment. Recent studies have demonstrated the high efficacy of PD-1 blockade in stage II–III dMMR/MSI rectal cancers, with notable rates of clinical complete response (cCR) or pCR.[18,19] These findings have opened up the possibility of sparing chemotherapy, radiotherapy, and even surgery for patients with dMMR/MSI LARC.

Despite the promising outcomes, the available data on neoadjuvant ICIs in dMMR/MSI colon and rectal tumors remain limited. Prior to the initiation of this trial, only the results with nivolumab plus ipilimumab had been published,[10] but ipilimumab was not accessible in China. Given that 29.4% of patients with dMMR/MSI metastatic CRC experienced early progression beyond pembrolizumab in the Keynote-177 trial,[7] concerns arose regarding the potential lack of benefit for all patients from PD-1 antibody monotherapy in the neoadjuvant setting, and there are no clinical trials combining chemotherapy with PD-1 antibody for neoadjuvant therapy.[20] Consequently, the decision was made to combine irinotecan and bevacizumab with toripalimab, based on their immunomodulatory effects.[21–24]

This study aimed to investigate the clinical activity and safety of neoadjuvant treatment with toripalimab in combination with irinotecan and bevacizumab in patients diagnosed with dMMR/MSI T4NanyM0 colon cancer (T4CC) and LARC. During the design of this trial, we acknowledged the potential publication of data from ongoing studies, which could provide additional evidence for neoadjuvant ICIs. Consequently, we implemented a multidisciplinary team-based doctor-patient shared decision-making process to determine whether to include irinotecan or bevacizumab in the treatment regimen, as well as when and whether to proceed with surgery. This process enabled the evaluation of the efficacy of toripalimab with or without irinotecan or bevacizumab and facilitated an exploratory analysis of whether the pCR rate was associated with therapeutic cycles.

RESULTS

Patient characteristics

From December 2020 to February 2023, a total of 26 patients were screened at two centers, with 22 of them deemed eligible for enrollment (Figure 1). The median age of the eligible patients was 47 years, ranging from 33 to 66. Among them, 16 were female and 6 were male. Three patients had a baseline Eastern Cooperative Oncology Group performance status of 1. Prior to confirmation of dMMR or MSI, three patients with colon cancer had received one cycle of neoadjuvant chemotherapy (capecitabine plus oxaliplatin).

In patients with T4NanyM0 colon cancer (colon cohort) (n = 14), 12 had clinical stage T4a, while 2 had clinical stage T4b. In patients with rectal cancer refusing up-front surgery or radiation therapy (rectum cohort) (n = 8), all had clinical stage T3. One patient in the rectum cohort had concomitant colon cancer located at the hepatic flexure; however, the characteristics of the colon tumor were not included in the colon cohort analysis as it required T4 stage criteria, as this patient (patient R3) was classified as T2N0 (Table 1).

Primary endpoint

As of December 1, 2024 (data cutoff), the median duration of follow-up was 35.6 months (interquartile range [IQR]: 29.9–41.3 months). In the colon cohort, per-protocol set (PPS) comprised 12 patients who underwent radical surgery and completed perioperative treatment. Among them, 8 patients (57.1% [95% confidence interval [CI] 28.9–82.3] in full analysis set [FAS] and 66.7% [34.9–90.1] in PPS) achieved a complete pathological response based on blinded, independent, central review (BICR). Two patients achieved cCR based on endoscopy, computed tomography, MRI, and arcinoembryonic antigen (CEA) level (Table 2; Figure 2). In the rectum cohort, PPS comprised 6 patients who underwent radical surgery, and all of them (75.0% [35.6–95.5] in FAS and 100.0% [51.7–100.0] in PPS) achieved a complete pathological response based on BICR. Two patients achieved a cCR and chose a "watch and wait" approach (Table 2; Figure 2). In both the colon and rectum cohorts, the primary endpoints of the first stage were met, indicating successful outcomes. Based on the Bayesian hierarchical model (Methods S1), the probability of success in this trial was estimated to be more than 99% after continuing enrollment in the second stage.

Secondary endpoints

In the FAS, the objective response rate (ORR), R0 resection rates, pCR rate assessed by local investigator, and pCR rate assessed by both BICR and local investigator were 78.6% (95% CI 48.8–94.3) and 87.5% (47.3–99.7), 85.7% (57.2–98.2) and 75.0% (35.6–95.5), 57.1% (28.9–82.3) and 75.0% (35.6–95.5), and 57.1% (28.9–82.3) and 75.0% (35.6–95.5), respectively, in the colon cohort and rectum cohort (Figure 3; Table 2). The summary of these endpoints in the PPS was presented in Table 2. In the PPS, the median time to surgery was 3.5 months (IQR

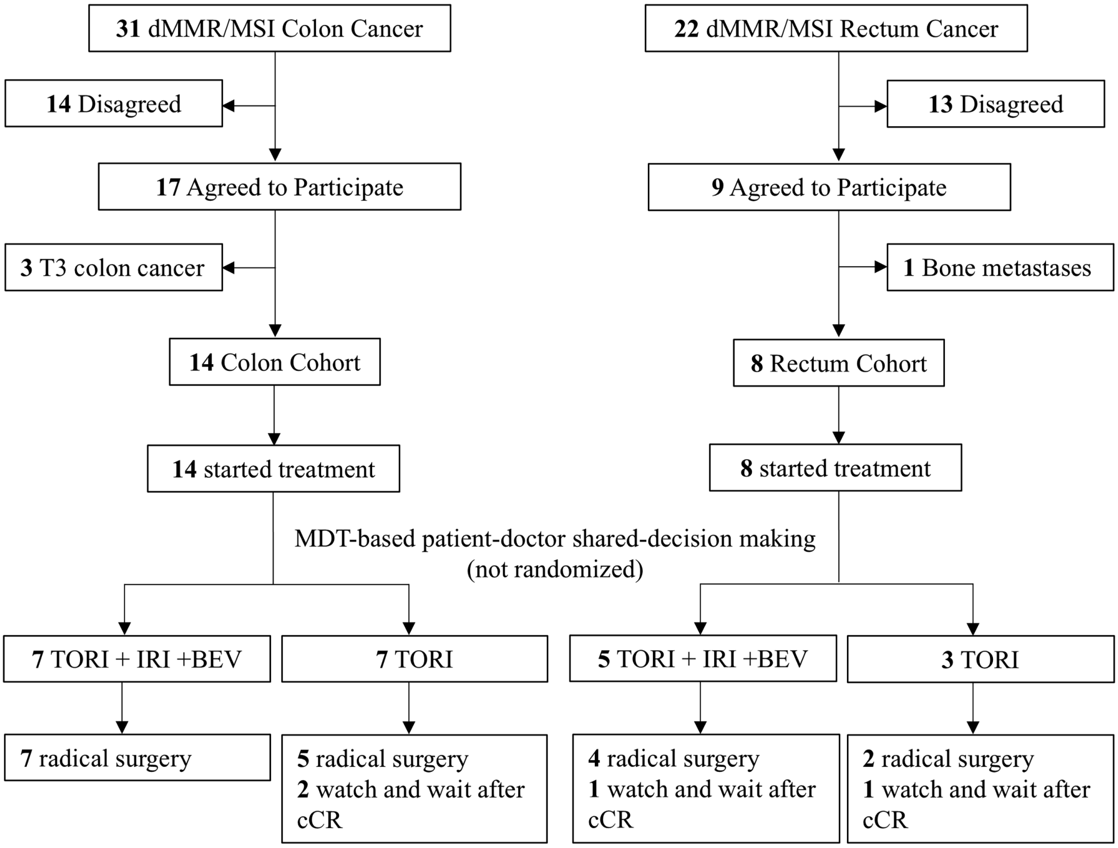

Figure 1. Study flow chart
Patients were enrolled from Beijing Cancer Hospital and The First Hospital of Jilin University. MDT, multidisciplinary team; TORI, toripalimab; IRI, irinotecan; BEV, bevacizumab; cCR, clinical complete response.

2.5–5.6 months) for the colon cohort and 6.0 months (IQR 4.4–7.3 months) for the rectum cohort. Survival endpoints were summarized in Table 2. In patients receiving radical surgery, no one experienced relapse or progression. In the 4 patients with cCR, only one had reginal lymph node progression (Table 3). We were unable to collect the data of life quality at every assessment time point due to COVID-19 epidemic. Individual patient data are summarized in Table 3.

Safety endpoint

Treatment-related adverse events (TRAEs) were summarized in Table S2. Sixteen patients (72.7%) experienced at least one TRAE of drugs during the study. Based on a shared decision-making strategy between doctors and patients, 12 patients received toripalimab in combination with irinotecan and bevacizumab (combination group), while 10 patients received toripalimab monotherapy (monotherapy group) in the neoadjuvant setting. Grade 3 and 4 TRAEs of drugs were only observed in the combination group, including neutropenia (25%), leukopenia (8.3%), and anemia (8.3%), and were not observed in the monotherapy group. Surgery-related adverse events were observed in two patients (2/18, 11.1%). Patient C10 experienced grade 4 intraoperative bleeding and grade 2 pancreatic fistula, and patient R1 developed grade 3 anastomotic fistula.

Exploratory analysis

The mutation status of *KRAS/NRAS/BRAF* was available for 16 patients. Among the 13 patients with *KRAS* mutation and *NRAS/BRAF* wild type, 9 underwent surgery, and 7 achieved pCR. One patient with the *BRAF* p.R239Q mutation and *KRAS/NRAS* wild type also achieved pCR. Among the 2 patients with *KRAS/NRAS/BRAF* wild type, one achieved pCR, while the other did not.

Although the rates of pCR (81.8%) and tumor regression grade (TRG) 0–1 (81.8%) in the combination group (n = 11) were comparable to that in the monotherapy group (71.4% and 100%, respectively, n = 7), ORR seemed higher in the combination group (91.7% vs. 60.0% in the monotherapy group) (Table S3). The median number of therapeutic cycles was 8.5. Patients who received $\geq$9 therapeutic cycles exhibited a higher pCR rate (100%) compared to those who received $\leq$8 cycles (63.6%). However, the rates of TRG 0–1 and ORR did not show a marked increase with additional cycles (Table S4).

DISCUSSION

Given that other neoadjuvant immunotherapy studies have not incorporated chemotherapy, this study investigated the efficacy and safety of combining PD-1 antibody with chemotherapy and

Table 1. Clinical characteristics of patients at baseline

	Overall (*n* = 22)	Colon cohort (*n* = 14)	Rectum cohort (*n* = 8)[a]
Age			
Median (IQR)	47 (33–66)	47 (34–66)	42 (26–61)
Range	19–70	26–69	19–70
Gender			
Male	15 (68.2%)	12 (85.7%)	3 (37.5%)
Female	7 (31.8%)	2 (14.3%)	5 (62.5%)
ECOG			
0	14 (63.6%)	8 (57.1%)	6 (75.0%)
1	8 (36.4%)	6 (42.9%)	2 (25.0%)
Primary tumor location			
Ascending colon	2 (9.1%)	2 (14.3%)	–
Hepatic flexure	6 (27.3%)	6 (42.9%)	–
Transverse colon	2 (9.1%)	2 (14.3%)	–
Descending colon	1 (4.5%)	1 (7.1%)	–
Sigmoid colon	3 (13.6%)	3 (21.4%)	–
Upper rectum	0 (0.0%)	–	0 (0.0%)
Middle rectum	3 (13.6%)	–	3 (37.5%)
Lower rectum	5 (22.7%)	–	5 (62.5%)
Clinical T stage			
T3	8 (36.4%)	0 (0.0%)	8 (100.0%)
T4a	12 (54.5%)	12 (85.7%)	0 (0.0%)
T4b	2 (9.1%)	2 (14.3%)	0 (0.0%)
Clinical N stage			
N negative	3 (13.6%)	2 (14.3%)	1 (12.5%)
N positive	19 (86.4%)	12 (85.7%)	7 (87.5%)
Histological differentiation			
Well to moderate differentiated	15 (68.2%)	8 (57.1%)	7 (87.5%)
Poor to un-differentiated	5 (22.7%)	4 (28.6%)	1 (12.5%)
Unable to determine	2 (9.1%)	2 (14.3%)	0 (0.0%)
Mucinous carcinoma/signet ring cell carcinoma compound			
Yes	0 (0.0%)	0 (0.0%)	0 (0.0%)
No	22 (100.0%)	14 (100.0%)	8 (100.0%)
Prior chemotherapy			
Yes (regimen)	3 (13.6%) (CAPOX)	3 (21.4%) (CAPOX)	0 (0.0%)
No	19 (86.4%)	11 (78.6%)	8 (100.0%)
Lynch syndrome			
Yes	8 (36.4%)	5 (35.7%)	3 (37.5%)
No	8 (36.4%)	4 (28.6%)	4 (50.0%)
Unknown	6 (27.3%)	5 (35.7%)	1 (12.5%)

Data are *n* (%) unless otherwise indicated. Percentages might not total 100 because of rounding. IQR, interquartile range; ECOG, Eastern Cooperative Oncology Group; CAPOX, capecitabine plus oxaliplatin.
[a]One patient with both a rectum and a colon tumor located at hepatic flexure was included in the rectum cohort, and characteristics of the colon tumor were not included in the colon cohort (T4 stage required) due to its T2 stage.

vascular endothelial growth factor (VEGF) antibody in locally advanced dMMR/MSI CRC.[20] The results demonstrated that the combination of toripalimab, irinotecan, and bevacizumab showed satisfactory activity and tolerability. A total of 22 patients were enrolled in the first stage of the study. In the Colon Cohort (*n* = 14), 12 patients underwent radical surgery, and 8 achieved a pCR. In the Rectum Cohort (*n* = 8), 6 patients underwent radical surgery, and all of them achieved pCR. Therefore, the primary endpoint was met in both the Colon and Rectum Cohorts.

T4 stage was considered an advanced stage and was associated with a poorer prognosis in CRC.[13] In dMMR/MSI T2–4 gastric cancers treated with durvalumab plus tremelimumab,

Table 2. Primary endpoint and secondary endpoints of efficacy in both FAS and PPS

	FAS (*n* = 22), % (95% CI)		PPS (*n* = 17), % (95% CI)	
Endpoints	Colon cohort (*n* = 14)	Rectum cohort (*n* = 8)	Colon cohort (*n* = 12)	Rectum cohort (*n* = 6)
Primary endpoint				
pCR rate by BICR	57.1 (28.9–82.3)	75.0 (35.6–95.5)	66.7 (34.9–90.1)	100.0 (51.7–100.0)
Secondary endpoints				
ORR	78.6 (48.8–94.3)	87.5 (47.3–99.7)	75.0 (42.8–94.5)	83.3 (36.5–99.1)
R0 resection rate	85.7 (57.2–98.2)	75.0 (35.6–95.5)	100.0 (73.5–100.0)	100.0 (51.7–100.0)
pCR rate by local assessment	57.1 (28.9–82.3)	75.0 (35.6–95.5)	66.7 (34.9–90.1)	100.0 (51.7–100.0)
pCR rate by both local assessment and BICR	57.1 (28.9–82.3)	75.0 (35.6–95.5)	66.7 (34.9–90.1)	100.0 (51.7–100.0)
TRG				
Grade 0	57.1 (28.9–82.3)	75.0 (35.6–95.5)	66.7 (34.9–90.1)	100.0 (51.7–100.0)
Grade 1	14.3 (1.8–42.8)	0.0 (0.0–36.9)	16.7 (2.1–48.4)	0.0 (0.0–45.9)
Grade 2	7.1 (0.2–33.9)	0.0 (0.0–36.9)	8.3 (0.2–38.5)	0.0 (0.0–45.9)
Grade 3	7.1 (0.2–33.9)	0.0 (0.0–36.9)	8.3 (0.2–38.5)	0.0 (0.0–45.9)
1-year event-free survival rate	100%	88% (64%–100%)	100%	100%
2-year event-free survival rate	100%	88% (64%–100%)	100%	100%
1-year disease-free survival rate	not applicable	not applicable	100%	100%
2-year disease-free survival rate	not applicable	not applicable	100%	100%
1-year overall survival rate	100%	100%	100%	100%
2-year overall survival rate	100%	100%	100%	100%

FAS, full analysis set; PPS, per-protocol set; pCR, pathological complete response; BICR, blinded, independent, central review; ORR, objective response rate; TRG, tumor regression grade.
See also Figure S1; Tables S3 and S4.

the pCR rate was 17% (1/6) in T4 tumors, significantly lower than the rate of 89% (8/9) in T2–3 tumors ($p = 0.011$).[25] It was unknown whether T4 stage is associated with a poor response to ICIs in CRC. Therefore, we focused on T4CC in this trial. All tumors showed shrinkage with an ORR of 78.6%. Among the 12 patients who underwent radical surgery, 8 achieved pCR. The pCR rate was comparable to the rates of 57.1% (4/7) from nivolumab with ipilimumab[10] and 76.9% (20/26) from toripalimab with or without celecoxib in resected T4 tumors.[11] Another retrospective study reported a pCR rate of 59.5% (22/38) in dMMR/MSI T4 CRC treated with PD-1 blockade-based therapy.[12] Taken together, these results demonstrate high anti-tumor efficacy and a promising pCR rate, even in T4CC. Importantly, even considering the high likelihood of recurrence after standard adjuvant chemotherapy,[26] dMMR T4CC appeared unlikely to relapse after neoadjuvant ICIs based on our results and those of other studies.[2,12] This emphasizes the necessity of ICIs before radical surgery in such patients. Based on the inspiring cCR rate in LARC treated with anti-PD-1 antibody,[19] the last two enrolled patients, C13 and C14, refused surgery and received 6-month treatment. They achieved cCR, chose to watch and wait, and had no disease recurrence after 22.3 and 21.9 months, respectively, which was consistent with the previous study.[27]

For LARC, the standard therapy is total neoadjuvant treatment, which includes chemoradiotherapy and induction or consolidation chemotherapy. However, considering the adverse events and unclear benefit of this approach in dMMR/MSI patients, two clinical trials were conducted and provided high pCR or cCR rates after anti-PD-1 antibody.[18,19] In our trial, among the 8 enrolled patients, 6 who underwent surgery achieved pCR, and 2 achieved cCR after 6 months of treatment. These results further support the use of neoadjuvant ICIs as a promising therapy for dMMR/MSI LARC. However, one patient with cCR experienced progression in reginal lymph node and no regrowth in primary tumor after stopping treatment. Though she responded to rechallenge with anti-PD-1 antibody and finally achieved cCR again, the optimal strategy for these patients remained to be explored.

As mentioned earlier, neoadjuvant ICI-based treatment has shown promising results in dMMR/MSI locally advanced CRC. To further advance this approach, it is important to investigate the preferred treatment regimen and treatment duration, as previous reports have provided limited insights into these aspects.

When selecting treatment for dMMR/MSI locally advanced CRC, it is important to consider both avoiding early progression and increasing efficacy. Fortunately, early progression is rare, and distal metastases had not been observed when treated with anti-PD-1 alone.[11,12,18,19,28–30] Additionally, these patients seemed unlikely to relapse after timely salvage surgery.[12,18] It should be noted that primary resistance mechanisms discovered in metastatic disease may not apply to locally advanced stages. A relevant example is the presence of *KRAS* mutations. In the first-line setting, pembrolizumab did not demonstrate longer progression-free survival compared to chemotherapy in *KRAS*-mutant patients.[7] In the late-line setting, the ORR was lower in patients with *KRAS* mutations (33%) than in the *RAS/BRAF* wild-type population (45%) receiving nivolumab.[3] However, among the 13 patients with *KRAS* mutations in our study,

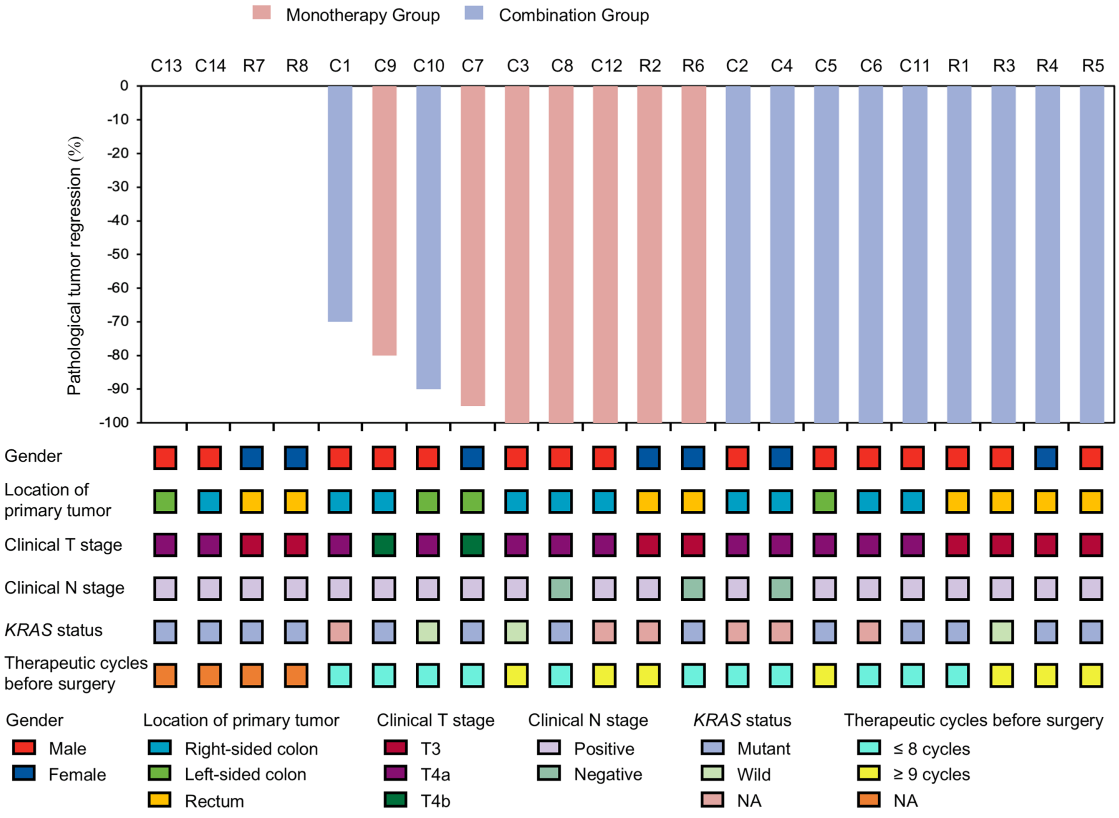

Figure 2. Waterfall plot of pathological tumor regression

Patients C13, C14, R7, and R8 had not undergone radical surgery as of the data cutoff date; therefore, pathological tumor regression information was not available for these cases.

See also Tables S3 and S4.

none experienced tumor growth, and the pCR rate in patients undergoing surgery was 75% (6/8), which was comparable to the overall population and consistent with findings in the NICHE-2 study.[31] Additionally, *KRAS* mutation status showed no association with tumor response and TRG.[12] An intriguing observation in this study was the *KRAS* mutation rate of 81.3%, which was numerically higher than those reported in other studies. In the Asian population, *KRAS* mutation rates have been documented to range between 48.9% and 56.6%.[32–34] By contrast, some large sample studies predominantly enrolling European and American patients, have reported *RAS* mutation rates of approximately 32.7%–35.6%.[7,35,36] In addition, previous studies have demonstrated that patients with Lynch syndrome or Lynch-like syndrome exhibit a higher prevalence of *KRAS* mutations compared to those with sporadic tumors.[37,38] In this present study, germline testing was performed on 16 patients, revealing 8 cases of Lynch syndrome (7 of which harbored *KRAS* mutations, 87.5%), 1 case of Lynch-like syndrome (carrying a *KRAS* mutation), and 7 cases of sporadic CRC (5 with *KRAS* mutations, 71.4%). Therefore, the higher proportion of *KRAS* mutations observed in this study may be attributed to ethnic variations, a relatively higher prevalence of Lynch syndrome or Lynch-like syndrome, and sampling errors. Furthermore, the disparities in *KRAS* mutation rates between Asian populations and those in Europe or America could reflect underlying genetic differences among these populations, underscoring the need for cautious interpretation when extrapolating our findings to other populations.

Combining chemotherapy with PD-1 blockade has been shown to potentially increase efficacy or overcome resistance in dMMR/MSI tumors in the palliative setting.[39,40] However, there is currently no data on the combination of ICIs with chemotherapy and anti-VEGF treatment in the neoadjuvant setting. In our exploratory analysis, although the pCR rates of the two groups were similar, the ORR of the combination group was much higher. This suggests that PD-1 antibody in combination with chemotherapy and bevacizumab may be more suitable for patients who need to minimize the tumor volume and reduce the surgical scope to the greatest extent, which is consistent with our findings in the palliative setting.[40] Furthermore, the addition of celecoxib, a COX-2 inhibitor intended to modulate the tumor microenvironment, did not significantly improve the efficacy of the PD-1 antibody.[11] When used for a relatively short duration, the addition of anti-CTLA-4 antibodies can enhance efficacy in the neoadjuvant setting.[41] However, the differences in pCR rates between PD-1 antibody monotherapy and PD-1 and CTLA-4

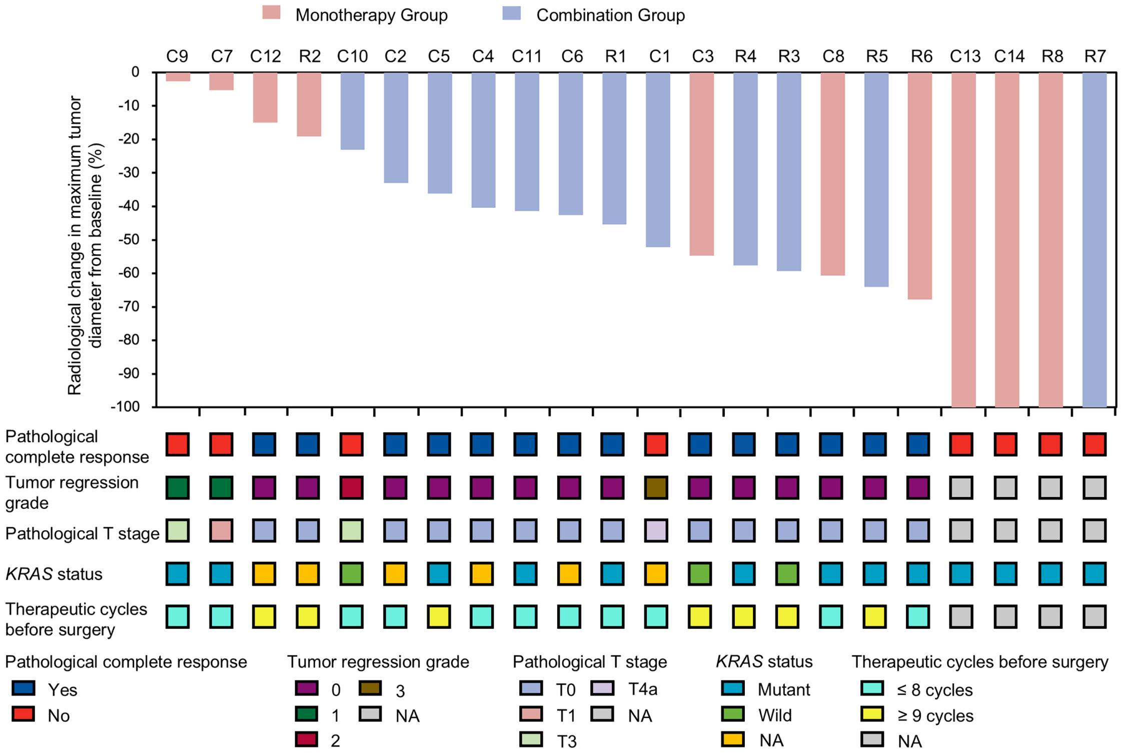

Figure 3. Waterfall plot of radiological change in maximum tumor diameter from baseline according to Response Evaluation Criteria In Solid Tumors v.1.1

Patients C13, C14, R7, and R8 had not received radical surgery by the data cutoff.
See also Tables S3 and S4.

dual blockade might be lesser if more ICI doses were delivered. It should be noticed that CTLA-4 antibody may not be necessary for patients with Lynch syndrome.[36,41] However, it is worth noting that the proportion of Lynch syndrome in the real world may be lower than that reported in published clinical trials, reflecting a biased referral pattern of clinical trial centers.[42]

Duration of treatment appeared to be an important factor associated with pCR in this study, as 100% of patients with therapeutic cycles $\geq$9 achieved pCR compared to 63.6% in those with therapeutic cycles $\leq$8. Additionally, four patients with 12 cycles of therapy achieved cCR. In another trial, only one out of three patients achieved pCR after 3 months of sintilimab treatment, while 11 out of 12 patients who received 6 or more months of treatment achieved cCR or pCR.[18] In the trial conducted by Cercek et al., all tumors were assessed as cCR at 6 months but not at 3 months[19] Based on the current evidence, it is reasonable to assume that 6 months of PD-1 antibody treatment may provide maximum benefit, but this hypothesis should be further validated in prospective clinical trials.

Although the short-term efficacy of ICIs in dMMR/MSI LACRC has been established several years ago, the long-term outcomes were much more important.[20] In this study with a median follow-up of 35.6 months, none of the 18 patients with resected tumors relapsed. Four patients chose watch and wait after cCR, and only one had lymph node progression but responded to rechallenge of PD-1 antibody and finally achieved cCR again. These results were in accordance with our previous findings and those of others.[43–45] The outstanding long-term efficacy of ICIs might change the clinical practice of dMMR/MSI LACRC.

In conclusion, this trial reported the efficacy and safety of toripalimab, with or without irinotecan and bevacizumab, in both dMMR/MSI T4CC and LARC. The results demonstrated a satisfactory pCR rate, and no patients experienced disease progression during the neoadjuvant treatment. After a long follow-up, no patients with resected tumors suffered from disease recurrence. Furthermore, no unexpected TRAEs or grade 4 or 5 TRAEs were observed. The higher pCR rate observed in patients receiving $\geq$9 cycles is encouraging, and we are currently designing a randomized trial to validate these findings.

Limitations of the study

This study had some limitations. First, the sample size was small. Second, the exploratory analysis concerning efficacy differences across various treatment regimens (monotherapy vs. combination) and therapeutic cycles ($\geq$9 vs. $\leq$8) was not included in the original study design. Therefore, we could not conclude whether or not irinotecan and bevacizumab or longer treatment duration contributed to a higher pCR rate. This topic of interest warrants further investigation in future studies. Third, the *KRAS* mutation rate observed in our study was 81.3%, which is higher than that reported in other American or European

Table 3. Individual patient data

	Gender	Age (year)	ECOG	Lynch syndrome	Clinical T and N stage	Primary site	Treatment regimen[b]	*KRAS*	*NRAS*	*BRAF*	Surgery	Cycles of TORI Pre-O	Cycles of TORI Post-O	TTS (month)	Pathological stage	TRG	Follow-up (month)
C1	male	66	1	unknown	T4aN+	hepatic flexure	TORI+IRI+BEV	unknown	unknown	unknown	yes	3	9	2.6	ypT4aN0	3	33.5[c]
C2	male	50	1	unknown	T4aN+	ascending colon	TORI+IRI+BEV	unknown	unknown	unknown	yes	4	2	2.5	ypT0N0	0	32.3[c]
C3	male	31	0	yes	T4aN+	transverse colon	TORI	wild	wild	wild	yes	9	0	5.6	ypT0N0	0	26.3[c]
C4	female	57	0	unknown	T4aN−	ascending colon	TORI+IRI+BEV	unknown	unknown	unknown	yes	3	6	3.1	ypT0N0	0	27.7[c]
C5	male	67	1	unknown	T4aN+	sigmoid colon	TORI+IRI+BEV	mutant	wild	wild	yes	9	0	6.0	ypT0N0	0	27.0[c]
C6	male	41	0	unknown	T4aN+	hepatic flexure	TORI+IRI+BEV	unknown	unknown	unknown	yes	3	9	2.0	ypT0N0	0	25.7[c]
C7	female	33	0	no[a]	T4bN+	sigmoid colon	TORI	mutant	wild	wild	yes	6	6	3.5	ypT1N1	1	24.0[c]
C8	male	34	0	no	T4aN−	hepatic flexure	TORI	mutant	wild	wild	yes	4	0	2.1	ypT0N0	0	20.5[c]
C9	male	69	0	no	T4bN+	hepatic flexure	TORI	mutant	wild	wild	yes	3	9	1.8	ypT3N1a	1	22.6[c]
C10	male	43	0	no	T4aN+	descending colon	TORI+IRI	wild	wild	wild	yes	3	7	1.8	ypT3N0	2	21.6[c]
C11	male	50	1	yes	T4aN+	hepatic flexure	TORI+IRI+BEV	mutant	wild	wild	yes	5	7	2.8	ypT0N0	0	16.0[c]
C12	male	26	1	yes	T4aN+	hepatic flexure	TORI	unknown	unknown	unknown	yes	9	0	3.9	ypT0N0	0	11.1[c]
C13	male	69	0	yes	T4aN+	sigmoid colon	TORI	mutant	wild	wild	no	12	NA	NA	NA	NA	8.0[d]
C14	male	34	1	yes	T4aN+	transverse colon	TORI	mutant	wild	wild	no	12	NA	NA	NA	NA	7.6[d]
R1	male	19	0	yes	T3N+	lower rectum	TORI+IRI+BEV	mutant	wild	wild	yes	7	5	4.0	ypT0N0	0	26.5[c]
R2	female	26	0	unknown	T3N+	middle rectum	TORI	unknown	unknown	unknown	yes	9	3	4.4	ypT0N0	0	24.2[c]
R3	male	70	0	no	T3N+	lower rectum	TORI+IRI+BEV	wild	wild	mutant	yes	9	0	7.3	ypT0N0	0	18.2[c]
R4	female	26	0	no	T3N+	lower rectum	TORI+IRI+BEV	mutant	wild	wild	no	12	0	9.7	ypT0N0	0	17.6[c]
R5	male	51	1	yes	T3N+	middle rectum	TORI+IRI	mutant	wild	wild	yes	10	0	6.4	ypT0N0	0	17.1[c]
R6	female	52	0	no	T3N−	middle rectum	TORI	mutant	wild	wild	yes	8	0	5.5	ypT0N0	0	14.3[c]
R7	female	33	0	no	T3N+	lower rectum	TORI+IRI+BEV	mutant	wild	wild	no	12	NA	NA	NA	NA	12.4[e]
R8	female	70	1	yes	T3N+	lower rectum	TORI	mutant	wild	wild	no	12	NA	NA	NA	NA	8.8[d]

TORI, toripalimab; IRI, irinotecan; BEV, bevacizumab; NA, not applicable; Pre-O, pre-operative; Post-O, post-operative; TRG, tumor regression grade.

See also Figure S1; Tables S3 and S4.

[a]Patient C7 was classified as having a Lynch-like syndrome, as he fulfilled the Amsterdam criteria but had no detectable germline mutations in *MLH1*, *MSH2*, *MSH6*, *PMS2*, or *EPCAM*.

[b]If surgery was planned, patients discontinued bevacizumab while continuing other medications. Surgery was scheduled no earlier than 6 weeks after the last dose of bevacizumab and 2–4 weeks after the last dose of toripalimab or irinotecan.

[c]These patients were disease-free at the last follow-up.

[d]Patients C13, C14, and R8 achieved clinical complete response (cCR) after 6-month treatment and chose watch and wait strategy. They had no evidence of disease recurrence at the last follow-up.

[e]Patient R7 achieved cCR after 6-month treatment and chose watch and wait strategy. However, she had regional lymph node progression and increasing CEA level 3 months later. She refused surgery and responded to rechallenge with anti-PD-1 blockade.

CellPress
OPEN ACCESS

studies. This might suggest a potential difference in genetic background, and thus, the conclusions should be cautiously extrapolated to other populations.

RESOURCE AVAILABILITY

Lead contact

Further information and requests for resources and reagents should be directed to and will be fulfilled by the lead contact, Lin Shen (shenlin@bjmu.edu.cn).

Materials availability

This study did not generate new unique reagents.

Data and code availability

- All data reported in this paper and any additional information required to reanalyze the data will be shared by the lead contact upon reasonable request. Specifically, de-identified individual patient-level data such as baseline clinical variables and grade of adverse events for each participant will be available upon request. Any additional information regarding individual participants that may result in breach of patient confidentiality will not be provided.
- This publication does not generate new code.
- Any additional information required to reanalyze the data reported in this work paper is available from the lead contact upon request.

ACKNOWLEDGMENTS

We thank Junshi Biosciences for providing JS001 for free and assisting in conducting this trial. We thank Yuxiao Tong for helping translating the protocol from Chinese to English. This study was funded by National Natural Science Foundation of China (nos. 82403443 and 91959130), Beijing Xisike Clinical Oncology Research Foundation (nos. Y-HH202101-0068 and Y-tongshu2021/ms-0040), and Beijing Hospitals Authority Clinical Medicine Development of Special Funding Support (no. ZLRK202327). This study was supported by Junshi Biosciences. The funders had no role in the design and conduct of the study; collection, management, analysis, and interpretation of the data; preparation, review, or approval of the manuscript; and decision to submit the manuscript for publication.

AUTHOR CONTRIBUTIONS

L.S. and J. Li. contributed to the conception and design of the study. All authors contributed to the acquisition, analysis, or interpretation of data. Z.W., X.W., X.Z., J. Leng., T.X., and M. Chen. wrote the drafting of the manuscript. Z.W., X.W., and J. Li. contributed to the critical revision of the manuscript for important intellectual content. Z.W., T.X., and M. Chen. performed the statistical analysis. All authors have read and agreed to the published version of the manuscript.

DECLARATION OF INTERESTS

The authors declare no competing interests.

STAR★METHODS

Detailed methods are provided in the online version of this paper and include the following:

- KEY RESOURCES TABLE
- EXPERIMENTAL MODEL AND STUDY PARTICIPANT DETAILS
- METHOD DETAILS
  - Patient eligibility
  - Subject allocation
  - Study design
  - Outcomes
  - Exploratory analysis
- QUANTIFICATION AND STATISTICAL ANALYSIS
- ADDITIONAL RESOURCES

SUPPLEMENTAL INFORMATION

Supplemental information can be found online at https://doi.org/10.1016/j.xcrm.2025.102296.

Received: December 22, 2024
Revised: May 9, 2025
Accepted: July 20, 2025
Published: August 15, 2025

REFERENCES

1. Sung, H., Ferlay, J., Siegel, R.L., Laversanne, M., Soerjomataram, I., Jemal, A., and Bray, F. (2021). Global Cancer Statistics 2020: GLOBOCAN Estimates of Incidence and Mortality Worldwide for 36 Cancers in 185 Countries. CA Cancer J. Clin. *71*, 209–249.
2. Chalabi, M., Verschoor, Y.L., van den Berg, J., Sikorska, K., Beets, G., Lent, A.V., Grootscholten, M., Aalbers, A., Buller, N., Marsman, H., et al. (2022). LBA7 Neoadjuvant immune checkpoint inhibition in locally advanced MMR-deficient colon cancer: The NICHE-2 study. Ann. Oncol. *33*, S1389.
3. Overman, M.J., Lenz, H.-J., Andre, T., Aglietta, M., Wong, M.K., Luppi, G., Van Cutsem, E., McDermott, R.S., Hendlisz, A., Cardin, D.B., et al. (2022). Nivolumab (NIVO) ± ipilimumab (IPI) in patients (pts) with microsatellite instability-high/mismatch repair-deficient (MSI-H/dMMR) metastatic colorectal cancer (mCRC): Five-year follow-up from CheckMate 142. J. Clin. Oncol. *40*, 3510.
4. Li, J., Deng, Y., Zhang, W., Zhou, A.P., Guo, W., Yang, J., Yuan, Y., Zhu, L., Qin, S., Xiang, S., et al. (2021). Subcutaneous envafolimab monotherapy in patients with advanced defective mismatch repair/microsatellite instability high solid tumors. J. Hematol. Oncol. *14*, 95.
5. Le, D.T., Kim, T.W., Van Cutsem, E., Geva, R., Jäger, D., Hara, H., Burge, M., O'Neil, B., Kavan, P., Yoshino, T., et al. (2020). Phase II Open-Label Study of Pembrolizumab in Treatment-Refractory, Microsatellite Instability-High/Mismatch Repair-Deficient Metastatic Colorectal Cancer: KEYNOTE-164. J. Clin. Oncol. *38*, 11–19.
6. Taieb, J., Bouche, O., André, T., Barbier, E., Laurent-Puig, P., Bez, J., Toullec, C., Borg, C., Randrian, V., Evesque, L., et al. (2022). LBA23 Avelumab versus standard second-line treatment chemotherapy in metastatic colorectal cancer (mCRC) patients with microsatellite instability (MSI): The SAMCO-PRODIGE 54 randomised phase II trial. Ann. Oncol. *33*, S1390–S1391.
7. Andre, T., Shiu, K.K., Kim, T.W., Jensen, B.V., Jensen, L.H., and Punt, C. (2020). Pembrolizumab in Microsatellite-Instability-High Advanced Colorectal Cancer. N. Engl. J. Med. *383*, 2207–2218.
8. Bertagnolli, M.M., Redston, M., Compton, C.C., Niedzwiecki, D., Mayer, R.J., Goldberg, R.M., Colacchio, T.A., Saltz, L.B., and Warren, R.S. (2011). Microsatellite instability and loss of heterozygosity at chromosomal location 18q: prospective evaluation of biomarkers for stages II and III colon cancer–a study of CALGB 9581 and 89803. J. Clin. Oncol. *29*, 3153–3162.
9. Ribic, C.M., Sargent, D., Moore, M.J., Thibodeau, S.N., French, A.J., Goldberg, R.M., Hamilton, S.R., Laurent-Puig, P., Gryfe, R., Shepherd, L.E., et al. (2003). Tumor microsatellite-instability status as a predictor of benefit from fluorouracil-based adjuvant chemotherapy for colon cancer. N. Engl. J. Med. *349*, 247–257.
10. Chalabi, M., Fanchi, L.F., Dijkstra, K.K., Van den Berg, J.G., Aalbers, A.G., Sikorska, K., Lopez-Yurda, M., Grootscholten, C., Beets, G.L., Snaebjornsson, P., et al. (2020). Neoadjuvant immunotherapy leads to pathological responses in MMR-proficient and MMR-deficient early-stage colon cancers. Nat. Med. *26*, 566–576.

11. Hu, H., Kang, L., Zhang, J., Wu, Z., Wang, H., Huang, M., Lan, P., Wu, X., Wang, C., Cao, W., et al. (2022). Neoadjuvant PD-1 blockade with toripalimab, with or without celecoxib, in mismatch repair-deficient or microsatellite instability-high, locally advanced, colorectal cancer (PICC): a single-centre, parallel-group, non-comparative, randomised, phase 2 trial. Lancet Gastroenterol. Hepatol. *7*, 38–48.

12. Xiao, B.Y., Zhang, X., Cao, T.Y., Li, D.D., Jiang, W., Kong, L.H., Tang, J.H., Han, K., Zhang, C.Z., Mei, W.J., et al. (2023). Neoadjuvant Immunotherapy Leads to Major Response and Low Recurrence in Localized Mismatch Repair-Deficient Colorectal Cancer. J. Natl. Compr. Canc. Netw. *21*, 60–66.

13. Gunderson, L.L., Jessup, J.M., Sargent, D., Greene, F.L., Stewart, A.K., and Stewart, A.K. (2010). Revised TN categorization for colon cancer based on national survival outcomes data. J. Clin. Oncol. *28*, 264–271.

14. Morton, D., Seymour, M., Magill, L., Handley, K., Glasbey, J., Glimelius, B., Palmer, A., Seligmann, J., Laurberg, S., Murakami, K., et al. (2023). Preoperative Chemotherapy for Operable Colon Cancer: Mature Results of an International Randomized Controlled Trial. J. Clin. Oncol. *41*, 1541–1552.

15. Cortes-Ciriano, I., Lee, S., Park, W.Y., Kim, T.M., and Park, P.J. (2017). A molecular portrait of microsatellite instability across multiple cancers. Nat. Commun. *8*, 15180.

16. Hause, R.J., Pritchard, C.C., Shendure, J., and Salipante, S.J. (2016). Classification and characterization of microsatellite instability across 18 cancer types. Nat. Med. *22*, 1342–1350.

17. Cercek, A., Dos Santos Fernandes, G., Roxburgh, C.S., Ganesh, K., Ng, S., Sanchez-Vega, F., Yaeger, R., Segal, N.H., Reidy-Lagunes, D.L., Varghese, A.M., et al. (2020). Mismatch Repair-Deficient Rectal Cancer and Resistance to Neoadjuvant Chemotherapy. Clin. Cancer Res. *26*, 3271–3279.

18. Chen, G., Jin, Y., Guan, W.L., Zhang, R.X., Xiao, W.W., Cai, P.Q., Liu, M., Lin, J.Z., Wang, F.L., Li, C., et al. (2023). Neoadjuvant PD-1 blockade with sintilimab in mismatch-repair deficient, locally advanced rectal cancer: an open-label, single-centre phase 2 study. Lancet Gastroenterol. Hepatol. *8*, 422–431.

19. Cercek, A., Lumish, M., Sinopoli, J., Weiss, J., Shia, J., Lamendola-Essel, M., El Dika, I.H., Segal, N., Shcherba, M., Sugarman, R., et al. (2022). PD-1 Blockade in Mismatch Repair-Deficient, Locally Advanced Rectal Cancer. N. Engl. J. Med. *386*, 2363–2376.

20. Veen, T., Kanani, A., Lea, D., and Søreide, K. (2023). Clinical trials of neoadjuvant immune checkpoint inhibitors for early-stage operable colon and rectal cancer. Cancer Immunol. Immunother. *72*, 3135–3147.

21. Heinhuis, K.M., Ros, W., Kok, M., Steeghs, N., Beijnen, J.H., and Schellens, J.H.M. (2019). Enhancing antitumor response by combining immune checkpoint inhibitors with chemotherapy in solid tumors. Ann. Oncol. *30*, 219–235.

22. Li, J.Y., Chen, Y.P., Li, Y.Q., Liu, N., and Ma, J. (2021). Chemotherapeutic and targeted agents can modulate the tumor microenvironment and increase the efficacy of immune checkpoint blockades. Mol. Cancer *20*, 27.

23. Terme, M., Pernot, S., Marcheteau, E., Sandoval, F., Benhamouda, N., Colussi, O., Dubreuil, O., Carpentier, A.F., Tartour, E., Taieb, J., et al. (2013). VEGFA-VEGFR pathway blockade inhibits tumor-induced regulatory T-cell proliferation in colorectal cancer. Cancer Res. *73*, 539–549.

24. Min, A.K.T., Mimura, K., Nakajima, S., Okayama, H., Saito, K., Sakamoto, W., Fujita, S., Endo, H., Saito, M., Saze, Z., et al. (2021). Therapeutic potential of anti-VEGF receptor 2 therapy targeting for M2-tumor-associated macrophages in colorectal cancer. Cancer Immunol. Immunother. *70*, 289–298.

25. Pietrantonio, F., Raimondi, A., Lonardi, S., Murgioni, S., Cardellino, G.G., Tamberi, S., Strippoli, A., Palermo, F., Prisciandaro, M., Randon, G., et al. (2023). INFINITY: A multicentre, single-arm, multi-cohort, phase II trial of tremelimumab and durvalumab as neoadjuvant treatment of patients with microsatellite instability-high (MSI) resectable gastric or gastroesophageal junction adenocarcinoma (GAC/GEJAC). J. Clin. Oncol. *41*, 358.

26. Cohen, R., Taieb, J., Fiskum, J., Yothers, G., Goldberg, R., Yoshino, T., Alberts, S., Allegra, C., de Gramont, A., Seitz, J.F., et al. (2021). Microsatellite Instability in Patients With Stage III Colon Cancer Receiving Fluoropyrimidine With or Without Oxaliplatin: An ACCENT Pooled Analysis of 12 Adjuvant Trials. J. Clin. Oncol. *39*, 642–651.

27. Yu, J.H., Xiao, B.Y., Li, D.D., Jiang, W., Ding, Y., Wu, X.J., Zhang, R.X., Lin, J.Z., Wang, W., Han, K., et al. (2024). Neoadjuvant camrelizumab plus apatinib for locally advanced microsatellite instability-high or mismatch repair-deficient colorectal cancer (NEOCAP): a single-arm, open-label, phase 2 study. Lancet Oncol. *25*, 843–852.

28. Xie, Y., Lin, J., Zhang, N., Wang, X., Wang, P., Peng, S., Li, J., Wu, Y., Huang, Y., Zhuang, Z., et al. (2023). Prevalent Pseudoprogression and Pseudoresidue in Patients With Rectal Cancer Treated With Neoadjuvant Immune Checkpoint Inhibitors. J. Natl. Compr. Canc. Netw. *21*, 133–142.

29. Zhang, X., Yang, R., Wu, T., Cai, X., Li, G., Yu, K., Li, Y., Ding, R., Dong, C., Li, J., et al. (2022). Efficacy and Safety of Neoadjuvant Monoimmunotherapy With PD-1 Inhibitor for dMMR/MSI rectangleH Locally Advanced Colorectal Cancer: A Single-Center Real-World Study. Front. Immunol. *13*, 913483.

30. Pei, F., Wu, J., Zhao, Y., He, W., Yao, Q., Huang, M., and Huang, J. (2023). Single-Agent Neoadjuvant Immunotherapy With a PD-1 Antibody in Locally Advanced Mismatch Repair-Deficient or Microsatellite Instability-High Colorectal Cancer. Clin. Colorectal Cancer *22*, 85–91.

31. Chalabi, M., Verschoor, Y.L., Tan, P.B., Balduzzi, S., Van Lent, A.U., Grootscholten, C., Dokter, S., Büller, N.V., Grotenhuis, B.A., Kuhlmann, K., et al. (2024). Neoadjuvant Immunotherapy in Locally Advanced Mismatch Repair-Deficient Colon Cancer. N. Engl. J. Med. *390*, 1949–1958.

32. Wang, Z., Zhang, Q., Qi, C., Bai, Y., Zhao, F., Chen, H., Li, Z., Wang, X., Chen, M., Gong, J., et al. (2022). Combination of AKT1 and CDH1 mutations predicts primary resistance to immunotherapy in dMMR/MSI-H gastrointestinal cancer. J. Immunother. Cancer *10*, e004703.

33. Hwang, H.S., Kim, D., and Choi, J. (2021). Distinct mutational profile and immune microenvironment in microsatellite-unstable and POLE-mutated tumors. J. Immunother. Cancer *9*, e002797.

34. Chida, K., Kawazoe, A., Kawazu, M., Suzuki, T., Nakamura, Y., and Nakatsura, T. (2021). A Low Tumor Mutational Burden and PTEN Mutations Are Predictors of a Negative Response to PD-1 Blockade in MSI-H/dMMR Gastrointestinal Tumors. Clin. Cancer Res. *27*, 3714–3724.

35. Fuca, G., Cohen, R., Lonardi, S., Shitara, K., Elez, M.E., and Fakih, M. (2022). Ascites and resistance to immune checkpoint inhibition in dMMR/MSI-H metastatic colorectal and gastric cancers. J. Immunother. Cancer *10*, e004001.

36. Andre, T., Elez, E., Lenz, H.J., Jensen, L.H., Touchefeu, Y., and Van Cutsem, E. (2025). Nivolumab plus ipilimumab versus nivolumab in microsatellite instability-high metastatic colorectal cancer (CheckMate 8HW): a randomised, open-label, phase 3 trial. Lancet *405*, 383–395.

37. Colle, R., Lonardi, S., Cachanado, M., Overman, M.J., Elez, E., Fakih, M., Corti, F., Jayachandran, P., Svrcek, M., Dardenne, A., et al. (2023). BRAF V600E/RAS Mutations and Lynch Syndrome in Patients With MSI-H/dMMR Metastatic Colorectal Cancer Treated With Immune Checkpoint Inhibitors. Oncologist *28*, 771–779.

38. Nakamori, S., Takao, M., Takao, A., Natsume, S., Iijima, T., Kojika, E., Nakano, D., Kawai, K., Inokuchi, T., Fujimoto, A., et al. (2024). Clinicopathological characteristics of Lynch-like syndrome. Int. J. Clin. Oncol. *29*, 944–952.

39. Chen, M., Wang, Z., Liu, Z., Liu, N., Fang, W., Zhang, H., Jin, X., Li, J., Zhao, W., Qu, H., et al. (2022). The Optimal Therapy after Progression on Immune Checkpoint Inhibitors in MSI Metastatic Gastrointestinal Cancer Patients: A Multicenter Retrospective Cohort Study. Cancers (Basel) *14*, 5158.

40. Chen, M., Wang, Z., Liu, Z., Deng, T., Wang, X., Chang, Z., Zhang, Q., Yang, W., Liu, N., Ji, Z., et al. (2023). PD-1/PD-L1 Inhibitor Plus Chemotherapy Versus PD-1/PD-L1 Inhibitor in Microsatellite Instability

Gastrointestinal Cancers: A Multicenter Retrospective Study. JCO Precis. Oncol. *7*, e2200463.

41. Xu, R.-H., Wang, F., Chen, G., Qiu, M., Ma, J., Liu, H., Mo, X., Li, Y., Wan, X., Luo, J., et al. (2024). Neoadjuvant treatment of IBI310 (anti-CTLA-4 antibody) plus sintilimab (anti-PD-1 antibody) in patients with microsatellite instability-high/mismatch repair-deficient colorectal cancer: Results from a randomized, open-labeled, phase Ib study. J. Clin. Oncol. *42*, 3505.
42. Kanani, A., Veen, T., Lea, D., Zaharia, C., Watson, M., Alexeeva, M., Thorsen, K., and Søreide, K. (2025). Neoadjuvant Immunotherapy Followed by Surgery Compared with Upfront Surgery Alone in Operable Colon Cancer with Deficient Mismatch Repair: Modeling Oncological Outcomes and Numbers Needed to Treat. Ann. Surg Oncol. *32*, 3068–3077.
43. Chalabi, M., van den Dungen, L.D.W., Verschoor, Y.L., Balduzzi, S., de Gooyer, P.G.M., Kok, N., Kerver, E., Grootscholten, C., Voest, E., Burger, J., et al. (2024). LBA24 Neoadjuvant immunotherapy in locally advanced MMR-deficient colon cancer: 3-year disease-free survival from NICHE-2. Ann. Oncol. *35*, S1217–S1218.
44. Hu, H., Zhang, J., Xie, X., Shi, L., Cai, Y., Li, W., Xie, Y., Wu, Z., Qin, G., Li, J., and Deng, Y. (2024). 542P Long-term outcomes of neoadjuvant toripalimab with or without celecoxib in patients with dMMR/MSI-H locally advanced colorectal cancer: 3-month treatment cohort of the randomized phase II PICC trial. Ann. Oncol. *35*, S452–S453.
45. Wang, Z., Cheng, S., Yao, Y., Liu, S., Liu, Z., Liu, N., Jin, Y., Zhang, Y., Yin, F., Han, G., et al. (2024). Long-term survivals of immune checkpoint inhibitors as neoadjuvant and adjuvant therapy in dMMR/MSI-H colorectal and gastric cancers. Cancer Immunol. Immunother. *73*, 182.
46. Berry, S.M., Broglio, K., Groshen, S., Berry, D.A., Fau - Berry, D.A., and Berry, D.A. (2013). Bayesian hierarchical modeling of patient subpopulations: efficient designs of Phase II oncology clinical trials. Clin. Trials *10*, 720–734.

STAR★METHODS

KEY RESOURCES TABLE

REAGENT or RESOURCE	SOURCE	IDENTIFIER
Biological samples		
Formalin-fixed paraffin-embedded archival tumor specimens	This manuscript	N/A
Chemicals, peptides, and recombinant proteins		
Toripalimab/JS001	Junshi Biosciences	www.junshipharma.com
Irinotecan	Pfizer	www.pfizer.com
Bevacizumab	Roche	www.roche.com
Deposited data		
Patient data	This manuscript	N/A
Software and algorithms		
SPSS software (version 23)	IBM	www.ibm.com
R version 4.3.0	The R Foundation	www.r-project.org

EXPERIMENTAL MODEL AND STUDY PARTICIPANT DETAILS

Chinese adults, both male and female, with histologically confirmed resectable rectal cancer who refused up-front surgery or radiation therapy (rectum cohort) or T4NanyM0 colon cancer (colon cohort) with deficiency of mismatch repair (dMMR) or microsatellite instability (MSI) were enrolled in the study. Demographic information was provided in Table 1, and no significant association of gender with the results of the study was found. All patients provided written informed consent prior to enrollment.

The study was conducted in accordance with the principles of the Declaration of Helsinki and the International Conference on Harmonization and Good Clinical Practice Guidelines. Each center had independent ethics committee that granted approval for the research protocol (Ethics Approval Number: 2019YJZ66 in Beijing Cancer Hospital, and 21K085-002 in The First Hospital of Jilin University).

METHOD DETAILS

Patient eligibility

Patients were eligible for enrollment if they had histologically confirmed colorectal cancer (CRC) with local confirmation of dMMR or MSI status, and met one of the following criteria: a) resectable rectal cancer classified as T3-4 or T1-2 with the refusal of up-front surgery or radiation therapy (rectum cohort), or b) resectable colon cancer classified as T4a-b (colon cohort). Key inclusion criteria included an age range of 18–75 years, adequate hematological, hepatic, and renal function, measurable disease as per the Response Evaluation Criteria In Solid Tumors (RECIST) v1.1 criteria, and no prior local treatment. Key exclusion criteria included prior blockade of PD-1/PD-L1/PD-L2 or CTLA-4 and any contraindication to toripalimab, irinotecan and bevacizumab. The full list of inclusion and exclusion criteria is provided in the protocol (Methods S1).

Subject allocation

This current phase Ib/II clinical trial is a single arm study with no control group. All patients are planned to receive toripalimab plus irinotecan and bevacizumab followed by radical surgery. However, this trial allows a doctor-patient shared-decision making process to determine whether to omit irinotecan or bevacizumab and the optimal surgery timing.

Study design

This dual-institution, open-label, phase Ib/II trial was conducted at two hospitals in China, namely Beijing Cancer Hospital and The First Hospital of Jilin University. Enrolled patients received toripalimab (3mg/kg d1), irinotecan (180mg/m2 d1) and bevacizumab (5mg/kg d1) every two weeks for three cycles, followed by radical surgery and adjuvant toripalimab at a dose of 240mg every 3 weeks. The entire perioperative treatment duration did not exceed six months. Based on the most recent findings from ongoing trials such as PICC and the study by Cercek et al. (11, 19), a multidisciplinary team (MDT)-based doctor-patient shared decision-making process was implemented in this trial. MDT members would inform patients about the latest advancements in the neoadjuvant immunotherapy field, and then discuss with them whether to omit irinotecan or bevacizumab at baseline and determine whether to repeat another three cycles of therapy or proceed to radical surgery at each tumor assessment timepoint until surgery was performed. If surgery was planned, patients discontinued bevacizumab while continuing other medications. Surgery was scheduled no earlier

than 6 weeks after the last dose of bevacizumab and 2–4 weeks after the last dose of toripalimab or irinotecan. This process also guided the decision of whether to proceed with radical surgery after the initial three cycles or to continue neoadjuvant treatment.

Radiological assessments of the tumor were conducted using thoracoabdominopelvic CT scans at baseline and after every three cycles of treatment. Patients in the Rectum Cohort underwent additional pelvic MRI. Clinical staging and changes in the maximum tumor diameter were evaluated by a senior radiologist (X.Z.). The extent of viable tumor remaining in the resected primary tumors (pathological regression) was assessed by a senior gastrointestinal pathologist (Y.S.).

Adverse events were monitored from the initiation of treatment until 90 days after the last dose of toripalimab or 30 days after surgery. The severity of adverse events was graded according to the Common Terminology Criteria for Adverse Events (CTCAE; version 5.0).

Following surgery, patients underwent physical examinations and measurement of carcinoembryonic antigen every three months for the first three years and every six months from years 4–5. Thoracoabdominopelvic CT scans for imaging surveillance were performed every six months during years 1–3 and every 12 months during years 4–5. A total colonoscopy was required within the first year, and a mandatory colonoscopy was conducted three years postoperatively.

Outcomes

The primary endpoint of this study was the pCR rate in all patients who received neoadjuvant therapy, as determined by a blinded, independent, central review (BICR). Secondary endpoints included the R0 resection rate, time to surgery (TTS), pCR rate assessed by the local investigator, pCR rate based on both BICR and the local investigator, tumor regression grade (TRG), objective response rate (ORR) based on RECIST 1.1 criteria, event-free survival (time from study treatment to the first documented inoperable disease progression, local or distant recurrence, or death), disease-free survival (time from the date of surgery to disease relapse or death), one-year and two-year disease-free survival rates (percentage of patients achieving disease-free survival for more than one and two years, respectively, from the date of surgery), one-year and two-year overall survival rates (percentage of patients surviving for more than one and two years, respectively, from the date of the first dose), and assessment of life quality (using the EORTC QLQ-C30 and EORTC QLQ-CR29 scales). Safety endpoints included incidence of treatment-related adverse events (TRAEs) associated with drugs and surgery.

Exploratory analysis

We retrospectively collected the mutation status of *KRAS*, *NRAS*, and *BRAF* from patients who underwent local testing, regardless of the testing platform and panel used. We assessed the pathological response and radiological response based on different gene statuses (wild-type vs. mutation), treatment regimens (toripalimab alone vs. toripalimab ± irinotecan ± bevacizumab) and therapeutic cycles ($\leq$8 vs. $\geq$ 9).

QUANTIFICATION AND STATISTICAL ANALYSIS

Sample size calculations were performed using a Bayesian hierarchical model[46] and were determined through a large-scale simulation study. The assumed pCR rate was 35% in the Rectum Cohort and 10% in the historical control group. In the Colon Cohort, the pCR rate was assumed to be 15%, while the historical control group had a rate of 3%. The Type I error rate was set at 5%, and the Type II error rate (statistical power) was set at 20% (i.e., achieving 80% power).

Enrollment was conducted in two stages to determine the required number of patients. In the first stage, 11 patients with colon cancer and 8 patients with rectal cancer were enrolled. If more than 2 out of the 11 patients in the Colon Cohort and more than 3 out of the 8 patients in the Rectum Cohort achieved pCR in the first stage, an additional 17 colon cancer patients and 8 rectal cancer patients would be enrolled in the second stage. Efficacy Endpoints in the Colon Cohort and Rectum Cohort were analyzed separately and summarized in full analysis set (FAS) (all patients who signed the informed consent form, met the inclusion criteria, and did not meet any exclusion criteria) and per-protocol set (PPS) (patients who signed the informed consent form, had no major protocol deviations, and had valid baseline data and primary endpoint measurements). Safety endpoints were analyzed in safety set (SS) (patients who received at least one dose of toripalimab) (Methods S1).

The pCR rate, ORR, R0 resection rate, and rates of different tumor regression grade (TRG) were summarized as frequencies and proportions. Two-sided 95% confidence intervals (CI) were calculated using the Clopper-Pearson method. Safety data were presented as the frequency and proportion of patients experiencing each event. Kaplan-Meier analysis was used to estimate event-free survival, disease-free survival, and overall survival, along with corresponding 95% CIs. The reverse Kaplan-Meier method was employed to calculate the median follow-up time and corresponding interquartile range (IQR). All statistical analyses were performed using SPSS software (version 23) and R software (version 4.3.0).

ADDITIONAL RESOURCES

This trial was the retrospectively registered at ClinicalTrials.gov as NCT04988191 on July 31, 2021. Prior to registration, a total of six patients were enrolled in the study, including five with colon cancer and one with rectal cancer, and other 16 patients were enrolled after registration (Table S1).

Cell Reports Medicine, Volume *6*

Supplemental information

Toripalimab, bevacizumab, and irinotecan in dMMR/MSI locally advanced colorectal cancer: First-stage results from a phase 1b/2 trial

Zhenghang Wang, Xicheng Wang, Xiaoyan Zhang, Jiahua Leng, Ming Cui, Ji Zhang, Quan Wang, Yu Sun, Ting Xu, Mifen Chen, Jian Li, and Lin Shen

Supplementary Materials

The PDF file includes:

Supplementary Figure S1

Supplementary Tables S1 to S5

Supplementary Methods S1

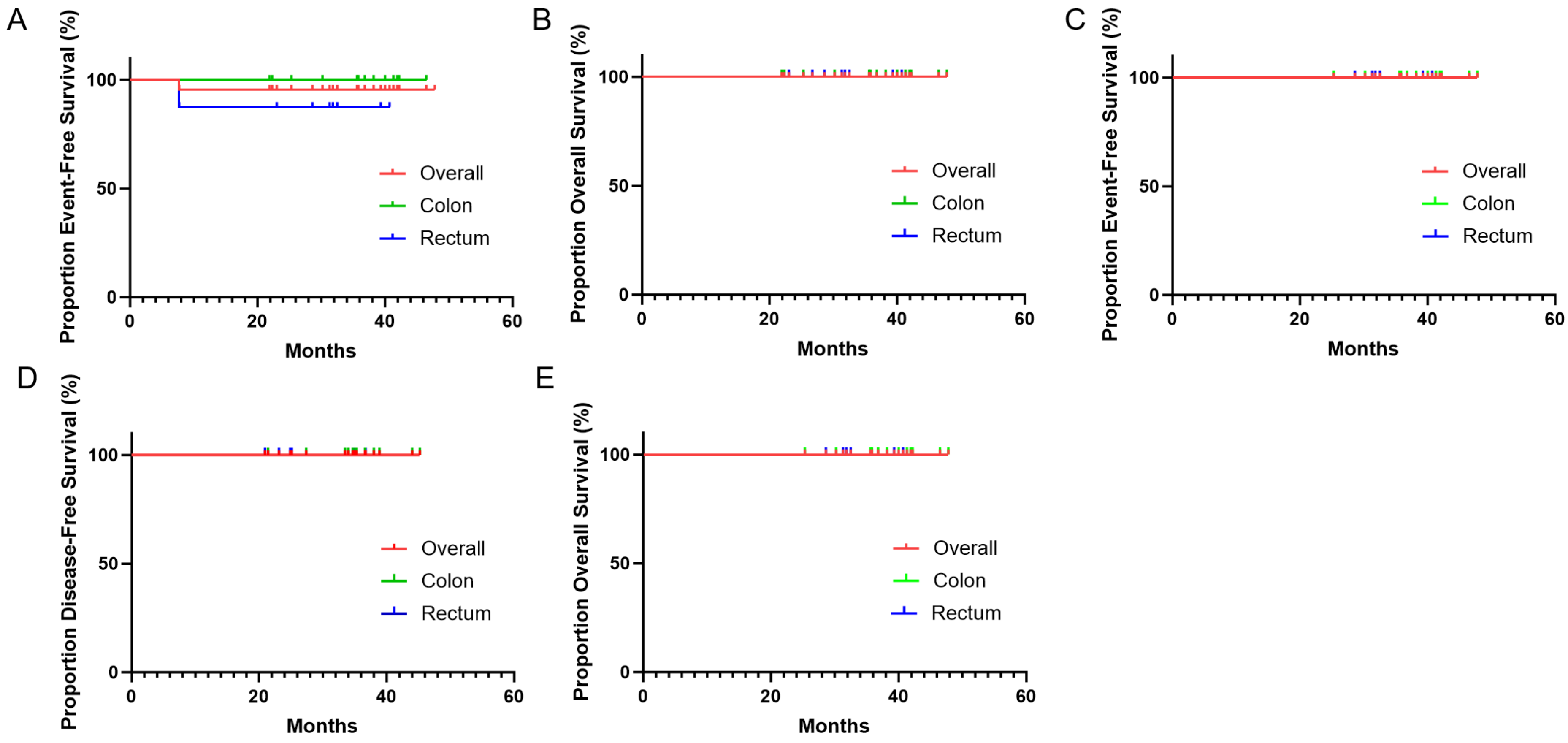

Supplementary Figure S1. Kaplan Meier curves of event-free survival (EFS), disease-free survival (DFS) and overall survival (OS) in the full analysis set (FAS) and per protocol set (PPS). A and B. EFS (A) and OS (B) of all patients (n=22), colon cancer patients (n=14) and rectal cancer patients (n=8) in FAS. C-E. EFS (C), DFS (D) and OS (E) of all patients (n=18), colon cancer patients (n=12) and rectal cancer patients (n=6) in PPS. Related to Tables 2 and 3.

Supplementary Table S1. Dates of Informed Consent Acquisition. Related to STAR Methods.

	Informed consent date	Before or after registration (2021-07-31)
C1	2020-12-24	Before
C2	2021-02-02	Before
C3	2021-06-09	Before
C4	2021-06-17	Before
C5	2021-07-06	Before
C6	2021-08-19	After
C7	2021-10-08	After
C8	2021-12-23	After
C9	2021-11-19	After
C10	2021-12-13	After
C11	2022-06-02	After
C12	2022-10-28	After
C13	2023-02-02	After
C14	2023-02-09	After
R1	2021-07-28	Before
R2	2021-09-06	After
R3	2022-04-18	After
R4	2022-04-20	After
R5	2022-04-29	After
R6	2022-07-19	After
R7	2022-09-22	After
R8	2023-01-11	After

Supplementary Table S2. Treatment related adverse events of drugs. Related to STAR Methods.

	All patients (n=22), n (%)		Combination (n=12), n (%)		Monotherapy (n=10), n (%)	
	Grade 1-2	Grade 3-4	Grade 1-2	Grade 3-4	Grade 1-2	Grade 3-4
Pruritus	7 (31.8)	0	3 (25.0)	0	4 (40.0)	0
Diarrhea	5 (22.7)	0	4 (33.3)	0	1 (10.0)	0
Alanine aminotransferase increased	4 (18.2)	0	3 (25.0)	0	1 (10.0)	0
Bilirubin increased	4 (18.2)	0	3 (25.0)	0	1 (10.0)	0
Leukopenia	6 (27.3)	1 (4.5)	6 (50.0)	1 (8.3)	0	0
Anemia	4 (18.2)	1 (4.5)	4 (33.3)	1 (8.3)	0	0
Abdominal pain	3 (13.6)	0	3 (25.0)	0	0	0
Fever	3 (13.6)	0	3 (25.0)	0	0	0
Rash	3 (13.6)	0	0	0	3 (30.0)	0
Nausea	3 (13.6)	0	3 (25.0)	0	0	0
Proteinuria	3 (13.6)	0	3 (25.0)	0	0	0
Decreased appetite	2 (9.1)	0	2 (16.7)	0	0	0
Hyperthyroidism	2 (9.1)	0	2 (16.7)	0	0	0
Aspartate aminotransferase increased	2 (9.1)	0	1 (8.3)	0	1 (10.0)	0
Hypothyroidism	3 (13.6)	0	3 (25.0)	0	0	0
Neutropenia	2 (9.1)	3 (13.6)	1 (8.3)	3 (25.0)	1 (10.0)	0
Fatigue	2 (9.1)	0	2 (16.7)	0	0	0
Hyperhidrosis	1 (4.5)	0	1 (8.3)	0	0	0
Swollen gums	1 (4.5)	0	1 (8.3)	0	0	0
Headache	1 (4.5)	0	1 (8.3)	0	0	0
Vomiting	1 (4.5)	0	1 (8.3)	0	0	0
Hypotension	1 (4.5)	0	1 (8.3)	0	0	0

Sinus tachycardia	1 (4.5)	0	1 (8.3)	0	0	0
Hypokalemia	1 (4.5)	0	1 (8.3)	0	0	0
Creatinine increased	1 (4.5)	0	1 (8.3)	0	0	0
Facial edema	1 (4.5)	0	1 (8.3)	0	0	0
Cardiac troponin I increased	1 (4.5)	0	1 (8.3)	0	0	0
Hair loss	2 (9.1)	0	2 (16.7)	0	0	0
Blood glucose increased	1 (4.5)	0	1 (8.3)	0	0	0

Supplementary Table S3. Efficacy summary in Monotherapy Group and Combination Group. Related to Figures 2, 3 and Table 3.

	FAS (n=22), % (95% CI)		PPS (n=18), % (95% CI)	
	Combination Group (n=12)	Monotherapy Group (n=10)	Combination Group (n=11)	Monotherapy Group (n=7)
pCR rate by BICR	75.0 (42.8-94.5)	50.0 (18.7-81.3)	81.8 (48.2-97.7)	71.4 (29.0-96.3)
pCR rate by local assessment	75.0 (42.8-94.5)	50.0 (18.7-81.3)	81.8 (48.2-97.7)	71.4 (29.0-96.3)
pCR rate by both local assessment and BICR	75.0 (42.8-94.5)	50.0 (18.7-81.3)	81.8 (48.2-97.7)	71.4 (29.0-96.3)
ORR	91.7 (61.5-99.8)	60.0 (26.2-87.8)	90.9 (58.7-99.8)	42.9 (9.9-81.6)
R0 resection rate	91.7 (61.5-99.8)	70.0 (34.8-93.3)	100.0 (71.5-100.0)	100.0 (59.0-100.0)
TRG				
Grade 0	75.0 (42.8-94.5)	50.0 (18.7-81.3)	81.8 (48.2-97.7)	71.4 (29.0-96.3)
Grade 1	0.0 (0.0-26.5)	20.0 (2.5-55.6)	0.0 (0.0-28.5)	28.6 (3.7-71.0)
Grade 2	8.3 (0.2-38.5)	0.0 (0.0-30.8)	9.1 (0.3-41.3)	0.0 (0.0-41.0)
Grade 3	8.3 (0.2-38.5)	0.0 (0.0-30.8)	9.1 (0.3-41.3)	0.0 (0.0-41.0)
1-year event-free survival rate	100%	92% (76%-100%)	100%	100%
2-year event-free survival rate	100%	92% (76%-100%)	100%	100%
1-year disease-free survival rate	Not appliable	Not appliable	100%	100%
2-year disease-free survival rate	Not appliable	Not appliable	100%	100%
1-year overall survival rate	100%	100%	100%	100%
2-year overall survival rate	100%	100%	100%	100%

FAS=full analysis set. PPS=per protocol set. pCR=pathological complete response. BICR=blinded, independent, central review. ORR=objective response rate. TRG=tumor regression grade.

Supplementary Table S4. Efficacy summary in patients receiving different therapeutic cycles. Related to Figures 2, 3 and Table 3.

	FAS (n=22), % (95% CI)		PPS (n=18), % (95% CI)	
	≤ 8 cycles (n=11)	≥ 9 cycles (n=11)	≤ 8 cycles (n=11)	≥ 9 cycles (n=7)
pCR rate by BICR	63.6 (30.8-89.1)	63.6 (30.8-89.1)	63.6 (30.8-89.1)	100.0 (59.0-100.0)
pCR rate by local assessment	63.6 (30.8-89.1)	63.6 (30.8-89.1)	63.6 (30.8-89.1)	100.0 (59.0-100.0)
pCR rate by both local assessment and BICR	63.6 (30.8-89.1)	63.6 (30.8-89.1)	63.6 (30.8-89.1)	100.0 (59.0-100.0)
ORR	72.7 (39.0-94.0)	81.8 (48.2-97.7)	72.7 (39.0-94.0)	71.4 (29.0-96.3)
R0 resection rate	100.0 (71.5-100.0)	63.6 (30.8-89.1)	100.0 (71.5-100.0)	100.0 (59.0-100.0)
TRG				
Grade 0	63.6 (30.8-89.1)	63.6 (30.8-89.1)	63.6 (30.8-89.1)	100.0 (59.0-100.0)
Grade 1	18.2 (2.3-51.8)	0.0 (0.0-28.5)	18.2 (2.3-51.8)	0.0 (0.0-41.0)
Grade 2	9.1 (0.3-41.3)	0.0 (0.0-28.5)	9.1 (0.3-41.3)	0.0 (0.0-41.0)
Grade 3	9.1 (0.3-41.3)	0.0 (0.0-28.5)	9.1 (0.3-41.3)	0.0 (0.0-41.0)
1-year event-free survival rate	100%	91% (73%-100%)	100%	100%
2-year event-free survival rate	100%	91% (73%-100%)	100%	100%
1-year disease-free survival rate	Not appliable	Not appliable	100%	100%
2-year disease-free survival rate	Not appliable	Not appliable	100%	100%
1-year overall survival rate	100%	100%	100%	100%
2-year overall survival rate	100%	100%	100%	100%

FAS=full analysis set. PPS=per protocol set. pCR=pathological complete response. BICR=blinded, independent, central review. ORR=objective response rate. TRG=tumor regression grade.

Supplementary Table S5. Efficacy summary according to different time to surgery (TTS) in PPS. Related to Figures 2, 3 and Table 3.

	PPS (n=18), % (95% CI)	
	≤ 3 months (n=11)	＞ 9 cycles (n=7)
pCR rate by BICR	63.6 (7/11)	100 (7/7)
pCR rate by local assessment	63.6 (7/11)	100 (7/7)
pCR rate by both local assessment and BICR	63.6 (7/11)	100 (7/7)
ORR	72.7 (8/11)	71.4 (5/7)
R0 resection rate	100 (11/11)	100 (7/7)
TRG		
Grade 0	63.6 (7/11)	100 (7/7)
Grade 1	18.2 (2/11)	0
Grade 2	9.1 (1/11)	0
Grade 3	9.1 (1/11)	0
1-year event-free survival rate	100%	100%
2-year event-free survival rate	100%	100%
1-year disease-free survival rate	100%	100%
2-year disease-free survival rate	100%	100%
1-year overall survival rate	100%	100%
2-year overall survival rate	100%	100%

PPS=per protocol set. pCR=pathological complete response. BICR=blinded, independent, central review. ORR=objective response rate. TRG=tumor regression grade.

Supplementary Methods S1. The latest protocol version before enrolment began. Related to STAR Methods.

Clinical study protocol

An Open-label, Multicenter, Single-arm Phase Ib/II Study to Evaluate Toripalimab (JS001) Efficacy and Safety of Combined Bevacizumab and Chemotherapy as Neoadjuvant Therapy in Patients with MSI-H (High Frequency Microsatellite Instability) or dMMR (Mismatch Repair Defects) Advanced Colorectal Cancer

Protocol No.: JS001-ISS-115

Version No.: 1.3

Version Date: 2020.5.22

Clinical Trial Institution:

Beijing Cancer Hospital and other

Principal Investigator: Lin Shen

Initiator of clinical trial: Beijing Cancer Hospital

Confidentiality Statement

The information contained in this document is proprietary and confidential. It may not be copied, circulated or otherwise disseminated

CONTENTS 2
PROTOCOL SUMMARY 6
List of Abbreviations and Associated Terms 22
1 STUDY BACKGROUND 25
1.1 Background Associated with Colorectal Cancer 25
1.1.1 Treatment of locally advanced rectal cancer 26
1.1.2 Treatment of locally advanced colon cancer 26
1.1.3 Colorectal cancer treatment with MSI-H (high frequency microsatellite instability) or dMMR (mismatch repair deficiency) 26
1.2 Immunotherapy Background 28
1.3 Background of Terdiplumab(JS001) Drug 30
1.3.1 Preclinical Studies 30
1.3.2 Clinical Safety 32
1.3.3 Clinical Efficacy 33
1.3.4 CLINICAL PHARMACOKINETICS, DOSE SELECTION AND IMMUNOGENICITY 34
1.4 STUDY RATIONALE 35
1.5 Benefit - Risk Assessment 37
2 Purpose of Study 38
2.1 Purpose of Study 38
2.2 Study Endpoints 39
3 STUDY DESIGN 42
3.1 Overall Design 42
3.2 Duration and End of Study 46
4 Study Population 47
4.1 Patient Inclusion Criteria 47
4.2 Patient Exclusion Criteria 48
4.3 Criteria for Discontinuation 51
4.4 Handling of Patients Discontinued 52
4.5 Removal Criteria 52
4.6 Replacement of Patients 52
4.7 Criteria for Terminating the Trial 52
5 Study Assessments 54
5.1 Screening/Baseline Period (Day -28 to Day -1) 54
5.2 TREATMENT PERIOD VISITS 55
5.2.1 Neoadjuvant therapy phase (predose 1) 55
5.2.2 Within 3 days before surgery and surgery (tumor imaging assessment can be performed within 7 days) 56
5.2.3 Adjuvant therapy phase 57
5.2.4 End of Treatment/Early Withdrawal Visit 59
5.2.5 Relapse-Free Survival Follow-up 60
5.2.6 Survival Follow-up 60
6 Study Drug and Administration 61
6.1 General Information of Study Drug 61
6.1.1 Toripalimab Injection(JS001) 61
6.1.2 Chemotherapy agents and bevacizumab 61

6.2 Management of Study Drug 61
6.2.1 Receipt and Storage 61
6.2.2 DISPOSITION OF STUDY DRUG 61
6.2.3 Dosing Regimen 61
6.2.4 TREATMENT COMPLIANCE 64
6.3 Concomitant Medications and Concomitant Treatments 65
6.4 Prohibited Medications and Treatments 65
7 Biomarker Testing 66
8 Tumor Assessment 67
9 Safety/Tolerability Assessment 69
9.1 Monitoring 69
9.2 JS001 Specific Adverse Event Management 70
9.2.1 Infusion Reactions 71
9.2.2 Serious allergic reactions 71
9.2.3 Immune-related Adverse Events 72
9.3 JS001 Dose Modification 73
9.4 Chemotherapy and Bevacizumab Safety Management and Dose Modifications 74
9.4.1 General Principles 74
9.4.2 CapeOX Safety Management and Dose Modifications 75
9.4.3 Bevacizumab Safety Management and Dose Modifications 76
9.5 Safety Parameters and Definitions 78
9.5.1 Safety/tolerability evaluation indicators 78
9.5.2 Definition of Adverse Events 78
9.5.3 Abnormal Laboratory Findings 79
9.5.4 Serious Adverse Events 79
9.5.5 JS001 Adverse Events of Special Interest (AESIs) 80
9.6 Causality Assessment of Adverse Events 81
9.7 Severity evaluation of adverse events 81
9.8 Recording and Reporting of Adverse Events and Serious Adverse Events 82
9.8.1 Recording and Reporting of Adverse Events 82
9.8.2 Reporting of Serious Adverse Events 83
9.8.3 Reporting and tracking of pregnancies 84
9.9 Documentation Procedures for Adverse Events 85
9.9.1 Infusion Reactions 85
9.9.2 Diagnosis and Signs and Symptoms 85
9.9.3 Adverse Events Secondary to Other Events 85
9.9.4 Persistent or Recurrent Adverse Events 86
9.9.5 Abnormal Laboratory Values 86
9.9.6 Abnormal Vital Signs Values 87
9.9.7 Abnormal liver function tests 87
9.9.8 Death 88
9.9.9 Pre-existing Disease Conditions 88
9.9.10 Disease Progression 88
9.9.11 Hospitalization or Prolonged Hospitalization 89
10 Data Management 90
10.1 Data Entry 90
10.2 Database Lock 90
11 Statistical Analysis 90

11.1 Sample Size 91
11.1.1 Colon cancer 91
11.1.2 Rectal cancer 92
11.2 Data Analysis Set 92
11.3 Analytical Procedures 92
11.3.1 Enrollment, Demographics, and Baseline Disease Characteristics 92
11.3.2 Efficacy Analyses 92
11.3.3 Biomarker Analysis 93
11.3.4 Safety Analysis 93
11.4 Real-time monitoring of efficacy 94
11.5 Extended Decision 95
12 STUDY MANAGEMENT 97
12.1 Ethical Considerations 97
12.2 Informed Consent 97
12.3 Compensation for Health Damage to Patients 97
12.4 Recording and Retention of Study Data 98
12.5 Return or destruction of study drug/therapeutic products 98
12.6 Quality Control and Quality Assurance 98
12.7 Monitoring and Auditing 98
12.8 Protocol Modifications 98
12.9 Study Termination 99
12.10 Study Summary Report 99
12.11 Confidentiality and Publication of Study Results 99
13 REFERENCES 101
14 APPENDICES 103
APPENDIX 1 STUDY FLOW CHART 103
Appendix 2 Eastern Cooperative Oncology Group(ECOG) Score Sheet 108
Appendix 3 Response Evaluation Criteria in Solid Tumors 109
APPENDIX 4 PRECAUTIONS FOR ALLERGIC REACTIONS 127
Appendix 5 Recommendations for replacement therapy for hypothyroidism 128
APPENDIX 6 Bayesian Hierarchical Model (Extension of Berry Method) 130
ATTACHMENT 6.1 MODELS AND METHODS 130
ATTACHMENT 6.2 Simulation Studies 132
Appendix7 EORTC QLQ-C30 Scale 135
Appendix 8 Questionnaire EORTC QLQ-CR29 Scale 137

List of Tables

Table1 JS001 Monitoring During Infusion 56
Table 2 Treatment Modification Guidelines for Infusion-Related Reactions 63
Table 3 Dose Modification Regimen for Capecitabine (X) in Combination with Oxaliplatin (O) According to Hematologic Toxicities During Planned Treatment 67
Table 4 Dose Modification Regimen for Hematotoxicity During Capecitabine (X) Treatment Cycles 67
Table 5 Dose Modifications for Oxaliplatin in Non-hematologic Toxicities 68
Table 6 Oxaliplatin Dose Modifications Associated with Neurotoxicity 68
Table7 Adverse Event Severity Grading Scale for Events Not Specified in CTCAE v5.0 74
Table 8 Critical value of invalid termination for monitoring of efficacy in colon cancer and rectal cancer 86

List of Figures

Figure 1 Study Design Flow Chart 37
Figure 2 MUCE versus Frequency School performing hypothesis testing (Freq) separately for each group 118

PROTOCOL SUMMARY

**Study title**	An Open-label, Multicenter, Single-arm Phase 1b/2 Study to Evaluate Toripalimab (JS001) in Combination with Bevacizumab and Chemotherapy as Neoadjuvant Therapy MSI-H (High-frequency Microsatellite Instability) or dMMR (Mismatch Modification)<br>Multiple defects) efficacy and safety in patients with advanced colorectal cancer
**Protocol No.**	JS001-ISS-115
**Study Phase**	Phase 1b/2
**Study duration**<br>**Time**	Duration of this study from the start of the informed consent form signed by the first patient (ICF) until the end of the study<br>Approximately 30 months.

**Study background**	According to the 2015 China Cancer Statistics, there were 376,300 new cases of colorectal cancer in 2015. In 2009, the Specialized Committee of Colorectal Cancer, Chinese Anti-Cancer Association published the clinicopathological data of 31,246 cases of colorectal cancer in 18 major hospitals, and rectal cancer still accounted for 61.95%. Because of the lack of specific symptoms of its early lesions, 70 to 80% of patients have locally advanced disease at the time of clinical diagnosis, including 30 to 40% of stage III patients.<br>According to the 2017 CSCO Guidelines for the Diagnosis and Treatment of Colorectal Cancer, the treatment strategy of preoperative concurrent chemoradiotherapy + surgery + adjuvant chemotherapy remains the standard treatment strategy for middle and low locally advanced rectal cancer (stage II and III). Many studies have shown that tumor pathological regression grade (TRG) is closely related to the prognosis of rectal cancer patients receiving preoperative radiotherapy. Pathologically confirmed pCR rates of locally advanced rectal cancer after preoperative chemoradiotherapy and radical surgery ranged from 7% to 38%. Preoperative radiotherapy combined with 5-FU/capecitabine effectively reduced the risk of local recurrence, but failed to effectively improve the overall survival rate of patients. No large phase III clinical trials have confirmed that preoperative chemoradiotherapy benefits patient survival. Therefore, many studies have tried to combine more intense chemotherapy and targeted drugs simultaneously on the basis of preoperative radiotherapy, as well as induction chemotherapy before preoperative radiotherapy and consolidation chemotherapy after preoperative radiotherapy in order to obtain a better outcome. However, multiple multicenter phase 3 randomized controlled studies have shown that increasing cytotoxic drugs fail to effectively improve pathological complete response (Pathological Complete Response, pCR) and long-term survival, and significantly increase treatment toxicity.<br>Colorectal cancers with MSI-H (high frequency microsatellite instability) or dMMR (mismatch repair deficiency) are able to benefit from anti-PD-1 therapy. This population accounts for 10 – 15% of all colorectal cancers, compared with only 5% of patients with advanced disease. In the field of colorectal cancer, the Le team conducted a phase II study in 2015 of pembrolizumab in refractory progressive metastatic cancer in 41 patients, with a median OS and PFS of 40% Vs 0% and 78% Vs 11% in colorectal cancer patients with mismatch repair deficiency (dMMR/MSI-H) versus normal mismatch repair (pMMR), respectively, and 5 months and 2.2 months, respectively. Whole genome sequencing detected tumor mutation burden (TMB), and the results showed that patients with dMMR had an average of 1782 somatic mutations per tumor, while patients with pMMR had only 73 somatic mutations, and a high somatic mutation burden was significantly associated with prolonged PFS. For patients with metastatic colorectal cancer who had received second-line therapy with dMMR, CheckMate-142 found an ORR of 31% with nivolumab alone and 55% with nivolumab and ipilimumab, a 1-year PFS of 71%, and an OS of 85%.

	In summary, in the field of colorectal cancer, the effective population of PD-1 inhibitors is mainly MSI-H/dMMR.<br>In the field of neoadjuvant therapy, PD-1/PD-L1 pathway therapy has achieved preliminary clinical results in multiple cancer types, including preliminary results of nivolumab as neoadjuvant therapy in resectable non-small cell lung cancer published in the New England Journal of Medicine (NEJM) in April 2018. In this Phase 1b study, 21 patients were treated with nivolumab at a dose of 3 mg/kg twice intravenously every two weeks before surgery and surgery was scheduled 4 weeks later. Positive treatment efficacy was initially observed in the trial, with radical surgery achieved in > 95% (20/21) of patients, and the major pathological response rate was 45% (9/20), 95% CI: 23% to 68%. Imaging assessment according to RECIST 1.1 revealed partial response in 10% (2/21) of patients and stable disease in 86% (18/21) of patients, without other safety concerns, and adverse reactions were generally consistent with most other studies of nivolumab. In 2018, ASCO reported a phase II study of single dose of pembrolizumab 200 mg as neoadjuvant therapy for head and neck squamous cell carcinoma, which observed a pCR rate of 50%; another neoadjuvant therapy of atezolizumab for myometrial invasive bladder cancer, which observed a pCR rate of 29%; it can be seen from multiple early studies that immunotherapy has achieved preliminary efficacy in multiple neoadjuvant therapies for solid tumors with acceptable feasibility and safety.<br>At present, there are no clinical results on the treatment of MSI-H/dMMR. At present, the efficacy of concurrent chemoradiotherapy + surgery + adjuvant chemotherapy, the standard treatment strategy for middle and low locally advanced rectal cancer (stage II and III), i.e., concurrent chemoradiotherapy + surgery + adjuvant chemotherapy, is still limited. Based on the clinical data of the above similar products in the population carrying MSI-H/dMMR, as well as the significant clinical benefits achieved by neoadjuvant therapy in the fields of NSCLC, head and neck squamous cell carcinoma and bladder cancer, it supports further exploration of the feasibility, safety and preliminary efficacy of JS001 as neoadjuvant therapy in colorectal cancer patients carrying MSI- H/dMMR. It is of great clinical significance to observe whether the blockade of PD-1/PD-L1 pathway can intensify tumor immunity, reduce tumor volume, achieve pathological response, reduce the complexity of surgery, improve the radical effect of surgery, thereby delaying the recurrence of the disease and prolonging the survival of patients.

**Study background**	In the field of neoadjuvant therapy, PD-1/PD-L1 pathway therapy has achieved preliminary clinical results in multiple cancer types, including preliminary results of nivolumab as neoadjuvant therapy in resectable non-small cell lung cancer published in the New England Journal of Medicine (NEJM) April 2018. In this Phase 1b study, 21 patients received two doses of nivolumab 3 mg/kg intravenously every two weeks prior to surgery 4 weeks later as planned. The trial initially observed a positive treatment effect, > 95% (20/21) of patients achieved radical surgery, with a major pathological response rate (Major Pathological Response) of 45% (9/20) , 95% CI: 23% to 68% , as assessed by imaging according to RECIST 1.1 , 10% (2/21) of patients had partial response and 86% (18/21) had stable disease without other safety concerns, and adverse reactions were generally consistent with nivolumab in most other studies. In 2018 ASCO reported a Phase 2 study of pembrolizumab 200 mg administered as a single dose as neoadjuvant therapy for head and neck squamous cell carcinoma, which observed 50% pCR rate; another atezolizumab administered as neoadjuvant therapy for myometrial invasive bladder cancer, which observed 29% pCR rate; From a number of early studies, it can be seen that immunotherapy has achieved preliminary efficacy in neoadjuvant therapy for a number of solid tumors with acceptable feasibility and safety.<br>At present, there are no clinical results for the treatment of MSI-H/dMMR, and the efficacy of concurrent chemoradiotherapy + surgery + adjuvant chemotherapy, the current standard treatment strategy for middle and low locally advanced rectal cancer (II , III), is still limited, based on the clinical data of the above similar products in the population carrying MSI-H/dMMR, as well as the significant clinical benefits achieved by neoadjuvant therapy in the fields of NSCLC , head and neck squamous cell carcinoma and bladder cancer, To further explore the feasibility, safety and preliminary efficacy of JS001 as neoadjuvant therapy in patients with MSI-H/dMMR carrying colorectal cancer, and observe whether blockade of PD-1/PD-L1 pathway can intensify tumor immunity, reduce tumor size, achieve pathological response, reduce surgical complexity, and improve the radical effect of surgery, thereby delaying the recurrence of the disease and prolonging the survival of patients is of great clinical significance.
**Study objectives**	Primary objective:<br>• To evaluate the rate of complete pathological response (pCR) by blinded, independent, central review (BICR) of toripalimab (JS001) combined with bevacizumab and chemotherapy as neoadjuvant therapy MSI-H (microsatellite instability-high) or dMMR (mismatch repair deficiency) for advanced colon cancer;<br>• To evaluate the rate of complete pathological response (pCR) with BICR of toripalimab (JS001) combined with bevacizumab and chemotherapy as neoadjuvant therapy in patients with MSI-H or dMMR advanced rectal

	cancer.<br>Secondary objectives<br>• To evaluate the preliminary efficacy of JS001 combined with bevacizumab and chemotherapy as neoadjuvant therapy in patients with MSI-H or dMMR advanced colon cancer: R0 resection rate, time to surgery, pCR rate assessed by pathologists at participating centers, pCR rate assessed by both BICR and pathology at participating centers, tumor regression grade (TGR), objective response rate assessed by investigators (ORR), event-free survival (EFS)), disease-free survival (DFS), 12 months and 24 months disease-free survival (DFS12 , DFS24), 12 months and 24 months survival (OS) rate;<br>• To evaluate the preliminary efficacy of JS001 combined with bevacizumab and chemotherapy as neoadjuvant therapy in patients with MSI-H or dMMR advanced rectal cancer: R0 surgery rate, time to surgery, pCR rate assessed by pathologists at participating centers, pCR rate assessed by BICR and pathology at participating centers, tumor regression grade (TGR), objective response rate assessed by investigators (ORR), event-free survival (EFS)), disease-free survival (DFS), 12 months and 24 months disease-free survival (DFS12 , DFS24), 12 months and 24 months survival (OS) rate;<br>• To evaluate the quality of life of JS001 combined with bevacizumab and chemotherapy as neoadjuvant therapy in patients with MSI-H or dMMR advanced colon and rectal cancer using EORTC QLQ-C30 and CR29 scales;<br>• To evaluate the safety of JS001 combined with bevacizumab and chemotherapy as neoadjuvant therapy in patients with MSI-H or dMMR advanced colon and rectal cancer;<br>Exploratory objectives<br>• To evaluate the correlation between immune-related biomarkers and efficacy in colon and rectal cancer.
**Overall design**	Multicenter, open-label, single-arm, two-cohort, two-stage trial design.

**Study Design**	This was a multicenter, open-label, single-arm study planned to enroll patients with MSI-H (high-frequency microsatellite instability) or dMMR (mismatch repair deficiency) advanced colorectal cancer to evaluate the safety and feasibility of toripalimab (JS001) combined with bevacizumab and chemotherapy as neoadjuvant therapy.<br>Imaging assessments and laboratory tests were performed during the screening period. According to the staging criteria and treatment roadmap of Chinese Guidelines for the Diagnosis and Treatment of Colorectal Cancer (Ministry of Health 2017), patients with stage T3-T4Nx rectal cancer or stage T1-2 < 12 cm from the anal verge who refused direct surgery or radiotherapy and patients with stage T 4a-b colon cancer who had MSI-H (high-frequency microsatellite instability) or dMMR (mismatch repair deficiency) could be enrolled in this study if they met the remaining inclusion and exclusion criteria and obtained informed consent.<br>Eligible patients received neoadjuvant therapy (see Administration) after enrollment, JS001 + Irinotecan + Bevacizumab, once every 2 weeks for 2 cycles; JS001 + Irinotecan, once every 2 weeks for 1 cycle; after which preoperative examination and preparation were perfected and patients received resection surgery, which should be performed within 4 weeks after neoadjuvant therapy. Specific medication regimens may be adjusted based on newly published clinical data and within the framework of shared decision-making between doctors and patients; if patients do not complete neoadjuvant therapy or disease progression or other reasons require early or delayed surgery, the decision to proceed with the next treatment requires discussion with the clinical multidisciplinary MDT.<br>Adjuvant therapy with JS001 may be continued 4 weeks (+/- 7 days) after the date of surgery every 3 weeks, and postoperative adjuvant therapy may be adjusted according to the actual clinical situation of the patient. Until disease recurrence, withdrawal of consent or intolerable toxicity, death, or study termination by the sponsor, whichever comes first, adjuvant JS001 therapy will continue for up to 9 cycles (up to 1 2 cycles for overall neoadjuvant and adjuvant JS001 therapy) if the patient remains disease-free, after which patients continue to undergo relapse and survival follow-up.<br>Pelvic MRI (for rectal cancer) and abdominal + thoracic CT to rule out distant metastasis were performed during the screening period, and enteroscopy was performed to identify the preoperative stage if necessary. Patients underwent an imaging assessment at 6 weeks ± 7 days during the neoadjuvant phase to assess lesion response to neoadjuvant therapy.<br>Specimen sampling and pathological assessment of resected tumor are conducted according to the standards of diagnosis and treatment. In addition to routine pathological

	examination and reporting, assessment of tumor regression grade is performed as shown in the table below. Complete pathological response (Pathological Complete Response, pCR) is defined as no active cancer cells found in any lesion or nodule after neoadjuvant therapy.

Grading	Description	Presentation
Grade 0	Complete regression	No residual tumor cells
Grade 1	Moderate regression	Single or small foci of residual tumor cells
Grade 2	Minor regression	Residual tumor with extensive fibrotic stroma
Grade 3	No regression	Extensive residual tumor, no or minimal tumor cell necrosis

In the adjuvant phase, CEA and CA19-9 are routinely performed every 6 weeks and MRI or CT imaging is performed every 12 weeks for 36 months after surgery until disease recurrence, withdrawal of consent, death, or study termination by the sponsor, whichever came first. Enteroscopy is performed if necessary to assess tumor recurrence. And unscheduled imaging or endoscopy may be performed if abnormal elevations of CEA or CA19-9 are found in laboratory tests. CEA and CA19-9 are routinely performed every 12 weeks after 36 months, and MRI or CT imaging and enteroscopy are performed every 24 weeks to assess tumor recurrence.

For patients with radiologically confirmed disease recurrence, survival follow-up will be performed every 3 months to collect information on subsequent anti-tumor treatment and survival until patient death, withdrawal of informed consent, loss to follow-up , or study termination by the sponsor, whichever occurs first.

Patients who discontinue treatment due to non-disease recurrence (e.g., toxicity) or reaching the maximum duration of treatment for JS001 will continue to undergo scheduled tumor assessments until radiologically confirmed disease recurrence, withdrawal of consent, patient death, or study termination by the originator, whichever occurs first. If patients receive other adjuvant therapy or observation follow-up after the completion of adjuvant therapy with JS001, disease recurrence follow-up and survival follow-up should be performed using the same tumor assessment method and frequency as previously described.

Observe any adverse events (AE) occurring in all patients during the clinical study, record the clinical symptoms, severity, occurrence time, ending time, duration, treatment measures and outcome, and determine their relationship with the study drug. The investigator should follow up all adverse events until resolution to baseline or

	better, the event is assessed as stable by the investigator, the patient is lost to follow-up, or the patient withdraws consent. Every effort should be made to follow all serious adverse events considered related to the study drug or study procedures until their final outcome can be reported.<br>No dose modifications of JS001 will be performed in this study. If a patient experiences an adverse event that requires a dose interruption, study treatment may be held 56 days after the last dose. If an adverse event causes JS001 to be suspended for 56 days after the last dose, the patient will discontinue treatment, and patients who discontinue treatment due to serious adverse reactions should be followed up until they return to normal or baseline levels, and patients who cannot tolerate it or patients who experience immune-related adverse reactions can undergo dose interruption or receive glucocorticoid therapy.<br>Prior to patient enrollment, tumor tissue samples from the patient 's primary tumor will be collected for MSI testing. Resected tumor tissue samples were subjected to pathological evaluation.

**Criteria for inclusion**	Patients meeting all of the following criteria are eligible to be enrolled<br>① Special inclusion criteria:<br>1) Histopathologically confirmed rectal or colon cancer:<br>a) Adenocarcinoma<br>b) Patients with T3-4 resectable rectal cancer<br>c) Patients with T1-2 stage < 12 cm from anal verge who refuse direct surgery or radiotherapy<br>d) Patients with T4a-b resectable colon cancer<br>2) MSI-H, defined as MSI-H confirmed by polymerase chain reaction (PCR) testing, or dMMR, before enrollment, more than 5 tissue FFPE slides are submitted for MSI testing and biomarker study. (Tumor tissue samples must be fresh or archived samples obtained 3 months prior to enrollment; Fresh tissue must be a core needle biopsy or excisional biopsy specimen. EBUS does not yield enough tissue for biomarker review).<br>3) Have at least one evaluable lesion (including measurable or non-measurable according to RECIST1.1) and did not receive local treatment<br>② General inclusion criteria:<br>4) Eastern Cooperative Oncology Group (ECOG) PS score 0-1;<br>5) Has fully understood the study and voluntarily signed the ICF;

6) Age 18 to 75 years, male or female;

7) Patients who, in the opinion of the investigator, are capable of complying with the protocol;

8) Laboratory values must meet the following criteria within 7 days prior to the start of neoadjuvant therapy:

a) Neutrophils ≥ 1.5 x 10 ^{9}/L;

b) Platelets ≥ 100 × 10 ^{9}/L;

c) Hemoglobin ≥ 100 g/L (no packed red blood cell transfusion within 2 weeks);

d) Serum creatinine ≤ 1.5 x upper limit of normal (ULN) and creatinine clearance ≥ 50 mL/min. Creatinine clearance was estimated based on the Cockcroft-Gault formula as follows:

$$= \frac{(140\ \text{Age})\ \text{Body weight (kg)} \times (\text{Female}\ 0.85)}{0.818\ \text{Serum creatinine}\ (\mu\text{mol/L})}$$

e) Aspartate aminotransferase (AST), alanine aminotransferase (ALT) ≤ 2.5× ULN;

f) Patients not receiving anticoagulant therapy: INR or aPTT ≤ 1.5 × ULN. Patients receiving prophylactic anticoagulant therapy may be enrolled if INR ≤ 2 ULN and aPTT is within normal values 14 days before the start of study treatment.

g) Serum total bilirubin ≤ 2 × ULN

9) Within 21 days prior to enrollment, women of childbearing potential must have a confirmed negative serum pregnancy test and agree to use effective contraception during study drug use and 60 days following the last dose of study drug. Examples of contraceptive methods with an annual contraceptive failure rate < 1% include bilateral tubal ligation, male sterilization, hormonal contraceptives that inhibit ovulation, hormone-releasing intrauterine devices, and copper ring intrauterine devices. Or double barrier contraception defined as condom containing spermicidal jelly, foam, suppository, or film; or diaphragm containing spermicide and male condom and diaphragm. Reliability of sexual abstinence should be evaluated relative to the duration of the clinical trial and the preferred lifestyle and lifestyle of daily living of the patient. Periodic abstinence (e.g., calendar day, ovulation, symptothermal, or post-ovulation methods) and withdrawal are not acceptable methods of contraception. In this protocol

Females of childbearing potential are defined as sexually mature females:

i. Hysterectomy or bilateral oophorectomy not performed;

ii. Spontaneous amenorrhea not continuing for 12 consecutive months

	(amenorrhea following cancer treatment does not exclude childbearing potential) (i.e., experienced at any time during the preceding 12 consecutive months Menstruation).
**Exclusion Criteria**	**Patients meeting any of the following criteria will not be included in this study:**<br>1) Patients who have previously received anti-programmed death receptor -1 (PD-1) antibody, anti-programmed death ligand - 1 (PD-L1) antibody, anti-programmed death ligand -2 (PD-L2) antibody, or anti-cytotoxic T lymphocyte-associated antigen -4 (CTLA-4) antibody therapy, including patients who have participated in JS001 clinical studies;<br>2) Presence of clinical manifestations of intestinal obstruction or active bleeding uncontrolled from the primary tumor.<br>3) Contraindications to bevacizumab or irinotecan.<br>4) Severe hypersensitivity to other monoclonal antibodies.<br>5) Have any active, known, or suspected autoimmune disease:<br>Patients who are hypothyroidism but on stable doses of thyroid hormone replacement therapy and those with type I diabetes whose blood glucose is controlled may be included in the study.<br>6) Uncontrolled pleural effusion, pericardial effusion, or moderate ascites;<br>7) History of idiopathic pulmonary fibrosis, organizing pneumonia (e.g., bronchiolitis obliterans), drug-induced pneumonia, idiopathic pneumonia, interstitial pneumonia, or evidence of active pneumonia on screening chest enhanced CT scan;<br>8) Major surgery 4 weeks prior to enrollment that has not fully recovered from previous surgery;<br>9) Active bleeding or coagulation abnormalities [activated partial thrombin time (aPTT) > 43 s, thrombin time (international normalized ratio (INR) > 1.5 × ULN], bleeding tendency or receiving thrombolytic or anticoagulant therapy; Prophylactic anticoagulation against an open intravenous infusion system was permitted as long as the drug activity had INR ≤ 2 × ULN and aPTT within normal limits 14 days prior to starting study treatment.<br>10) Patients who have received previous allogeneic stem cell or parenchymal organ transplantation;<br>11) Any significant clinical and laboratory abnormalities that, in the opinion of the investigator, affect patients' safety, such as: uncontrolled active infection, uncontrolled diabetes mellitus, hypertension that cannot be controlled within the following ranges by monotherapy (systolic blood pressure < 140 mmHg ,

	diastolic blood pressure < 90 mmHg), II or higher peripheral neuropathy, congestive heart failure, cardiac disease as defined by the New York Heart Association (II or higher), myocardial infarction within 3 months before enrollment, unstable arrhythmia, unstable angina pectoris, chronic kidney disease, thyroid dysfunction, etc., previous or concurrent other malignancies;<br>12) History of electrolyte disturbances such as uncorrectable serum potassium, calcium, or magnesium<br>13) Known human immunodeficiency virus (HIV) infection;<br>14) Active hepatitis B virus (HBV), hepatitis C virus (HCV) infection:<br>a) Active viral hepatitis is defined as HBV infection with hepatitis B virus deoxyribonucleic acid (HBV DNA) ≥ 1000 cps/ml or 200 IU/ml or above their upper limit of normal; or HCV infection;<br>b) Patients with previous HBV infection or cured HBV infection: Defined as hepatitis B core antibody (HBcAb) positive and hepatitis B surface antigen (HBsAg) negative], can participate in this study. HBV DNA test results of these patients must be < 1000cps/ml or 200 IU/ml or lower than the upper limit of normal, and the doctor judges that the disease is stable and antiviral therapy is not required;<br>c) Patients who tested positive for HCV antibodies can be included in the study only if they are tested negative for hepatitis C virus ribonucleic acid (HCV RNA).<br>d) Patients cannot be enrolled if they are receiving antiviral therapy at the time of enrollment and are required to maintain stable antiviral therapy throughout the study, and any patients with concerns about compliance are not enrolled.<br>15) Female patients who are pregnant or lactating, or unwilling to take contraception during the trial;<br>16) History of other malignancy within 5 years prior to enrollment, except for those with negligible risk of metastases or death [such as malignancy with expected 5-year overall survival (OS) > 90%] and expected to be cured with treatment (e.g., appropriately treated cervical carcinoma in situ, basal or squamous cell skin cancer, localized prostate cancer treated with radical surgery, ductal carcinoma in situ treated with radical surgery);<br>17) Immunosuppressants, or systemic, or absorbable topical hormonal therapy for immunosuppressive purposes (dose > 10 mg/day prednisone or other equal-potency hormone) and continuing 2 weeks prior to enrollment;<br>18) Patients with active pulmonary tuberculosis (TB) who are receiving anti-tuberculosis treatment or who have received anti-tuberculosis treatment 1

	years prior to screening;<br>19) Active infection or treatment with oral or intravenous antibiotics 2 weeks prior to the start of neoadjuvant therapy, with the exception of prophylactic use;<br>20) Any anti-infective vaccine (eg, influenza vaccine, varicella vaccine, etc.) within 4 weeks prior to the start of neoadjuvant therapy;<br>21) Receipt of other investigational agents or participation in a clinical study for other treatment purposes within 28 days prior to the start of neoadjuvant therapy;<br>22) Any other disease, metabolic disorder, physical examination finding, or laboratory abnormality that would contraindicate the use of the trial drug, or compromise the reliability of the study results, or place the patient at high risk for treatment complications, or compromise patient compliance.
**Number of patients**	**Colon cancer**<br>In this study, it is planned to enroll 28 patients with colon cancer who meet the study inclusion criteria to receive neoadjuvant therapy with toripalimab (JS001) combined with bevacizumab and chemotherapy. The above 28 patients with colon cancer will be enrolled in two stages, 11 in the first stage and 17 in the expansion stage.<br>**Rectal cancer**<br>This study plans to enroll 16 patients with rectal cancer who meet the study inclusion criteria to receive neoadjuvant therapy with toripalimab (JS001) combined with bevacizumab and chemotherapy. The above 16 patients with rectal cancer will be enrolled in two stages, 8 in the first stage and 8 in the expansion stage.
**Investigational drug**	Toripalimab (abbreviated as JS001, also known as recombinant humanized anti- PD-1 monoclonal antibody): specification: 240 mg/6 mL/vial; sterile water for injection; shelf life: 24 months; production date: subject to the production date shown on the product package; storage condition: protected from light, 2 ~ 8 ° C refrigerated.
**Dosing**	**Neoadjuvant phase:**<br>**Combination Phase of JS001 + Irinotecan + Bevacizumab for 2 Cycles**<br>JS001 Administered dose: 3 mg/kg via intravenous infusion on d1 every 2 weeks,<br>Bevacizumab Dose administered: 5 mg/kg by intravenous infusion every 2 weeks<br>Irinotecan administered dose: 180 mg/m^2 via intravenous drip over 2 hours, d1, every 2 weeks.

	**JS001 + Irinotecan Combination Phase, One Cycle**<br>JS001 Administered dose: 3 mg/kg via intravenous infusion on d1 every 2 weeks<br>Dose of Irinotecan: 180 mg/m 2 via intravenous drip 2 hours, d1, every 2 weeks<br><br>**Adjutant phase**<br>JS001 is given by intravenous infusion at a dose of 240mg every 3 weeks for up to 9 cycles.<br><br>Carefully check and confirm that each bottle of JS001 Injection is not damaged and that the solution in the bottle does not appear solidified, turbid, or precipitated before use.<br>It is recommended to withdraw the corresponding volume of study drug with a syringe and inject 100 mL of normal saline, gently invert the diluted solution 3 to 5 times to mix, avoiding forceful shaking, and use an in-line filter (0.2 or 0.22 μm) for intravenous drip for at least 60 minutes after mixing. Because this product does not contain antimicrobial preservatives, the formulated solution must be performed under aseptic conditions. If the prepared diluted solution cannot be used immediately, it can be stored in a refrigerator at 2 to 8 ° C for a maximum of 16 hours or 4 hours at room temperature.<br>Bevacizumab and Irinotecan, refer to the package insert for the specific configuration.

**Efficacy evaluation indicators**	The following efficacy evaluation indicators are applicable to colon cancer and rectal cancer.<br>**Primary efficacy indicator**<br>• BICR pCR rate<br>**secondary efficacy measure**<br>• R0 Surgery rate<br>• Time to Surgery<br>• pCR rate assessed by participating site pathologists<br>• pCR rate assessed by both BICR and participating site<br>• Tumor regression grade (TGR)<br>• Investigator-assessed objective response rate (ORR)<br>• Event-free survival assessed by investigator (EFS)<br>• Disease-free survival (DFS)<br>• 12 month or 24 months disease-free survival (DFS12 , DFS24)<br>• 12 months or 24 months survival (OS) rate<br>• Quality of life score (using EORTC QLQ-C30 and CR29 scales)
**Safety/ Tolerability measures**	**Safety/Tolerability measures included:**<br>• AEs (including SAEs and AESIs) assessed according to CTCAE v5.0;<br>• Vital signs, ECG and physical examination;<br>• Clinical laboratory tests;
**Exploratory evaluation indicators**	Tumor tissue samples before neoadjuvant therapy, surgery, and recurrence (optional), as well as peripheral blood samples before neoadjuvant therapy, surgery, and postoperative follow-up were collected for comprehensive biomarker analysis, not limited to ctDNA, TMB, and PD-L1 expression, to evaluate the effect of JS001 treatment on the colorectal cancer microenvironment. And the dynamic changes in the types of T-cell clones within and in the periphery of the tumor.
**Analysis Set**	The analysis sets were differentiated for colon cancer patients and rectal cancer patients according to the following data analysis set definitions.<br>Full Analysis Set (FAS): all patients who sign informed consent and meet the inclusion and don't meet exclusion criteria.<br>Per-Protocol Set (PPS): all patients who have signed informed consent, have no major protocol violations, and have valid baseline and primary endpoint measures.<br>Safety Set (Safety Set, SS): patients enrolled in the trial and treated with at least one dose of study drug.

**Sample Size**	**The sample size calculation was based on a Bayesian hierarchical model (an extended approach by Berry et. al. (2013)), and the sample size for both colorectal cohorts was set as follows by data simulation:**<br>**Colon cancer**<br>This study plans to enroll 28 patients with colon cancer who meet the study inclusion criteria and receive neoadjuvant therapy with toripalimab (JS001) combined with bevacizumab and chemotherapy, and will provide 76% power to prove pCR rate in patients with colon cancer receiving this neoadjuvant therapy is superior to historical control at a one-sided statistical significance level of 0.05. The above sample size calculations are based on the following assumptions:<br>● Blinded Independent Central Pathology Evaluation of Colon Cancer Patients Receiving Neoadjuvant Therapy with Toripalimab (JS001) in Combination with Bevacizumab and Chemotherapy pCR rate 15%<br>● Historical control blinded independent central pathology assessment pCR rate 3%<br>The above 28 patients with colon cancer will be enrolled in two stages, 11 in the first stage and 17 in the expansion stage. 11 patients enrolled in Stage I will be used for real-time efficacy monitoring.<br>**Rectal cancer**<br>This study plans to enroll 16 patients (including 8 patients in Phase 1 and 8 patients in Extension Phase) with rectal cancer who meet the study entry criteria to receive neoadjuvant therapy with toripalimab (JS001) combined with bevacizumab and chemotherapy, and will provide 80% power to demonstrate pCR rate in patients with rectal cancer receiving this neoadjuvant therapy is superior to historical control at a one-sided statistical significance level of 0.05. The above sample size calculations are based on the following assumptions:<br>● Blinded Independent Central Pathology Evaluation of Rectal Cancer Patients Receiving Neoadjuvant Therapy with Toripalimab (JS001) Combined with Bevacizumab and Chemotherapy The pCR rate was 35%<br>● Historical control blinded independent central pathology assessment pCR rate 10%<br>The above 16 patients with rectal cancer will be enrolled in two stages, with 8 planned in the first stage and 8 planned in the expansion stage. 8 patients enrolled in Stage I will be used for real-time efficacy monitoring.
	Unless otherwise specified, rectal cancer patients and colon cancer patients will be summarized and analyzed separately in this study. Descriptive statistics: continuous variables (e.g., age) will be statistically described using the number of observations, mean, median, standard deviation, minimum and maximum; categorical variables will

**Statistical Analysis**	be statistically described using the frequency and percentage of each category. Specific analytical methods will be described in the Statistical Analysis Plan.<br>**Efficacy Analysis**<br>BICR assessed pCR rate was the primary efficacy measure in this study. Corresponding 95% confidence intervals were calculated using the Clopper-Pearson method for pCR rates based on the FAS set. Sensitivity analyses will be performed on the BICR pCR rate based on the PPS set. At the same time, the results of the Bayesian hierarchical model will play an auxiliary supporting role.<br>For the secondary efficacy measures, FAS set was the primary analysis set and PPS was the sensitivity analysis set.<br>For the secondary efficacy endpoints R0 surgery rate, pCR rate assessed by the pathologist at the participating site, pCR rate by both BICR and participating site, ORR, the same statistical analysis method as the primary efficacy endpoint was used.<br>95% confidence intervals will be calculated for each TGR grade for the secondary efficacy endpoint of tumor regression grade using the same statistical analysis as for the primary efficacy endpoint.<br>Kaplan-Meier method will be used for secondary efficacy measures of time to surgery, investigator assessments EFS, DFS, and OS, calculating the corresponding median time. The 95% confidence intervals will be estimated by the Brookmeyer-Crowley method using a log-log function transformation to reach a normal approximation. For DFS12 and DFS24, the rate values at specific times were first estimated with KM method, then corresponding 95% confidence intervals were estimated using Greenwood formula.<br>Descriptive statistics were used to analyze the secondary efficacy measure quality of life scores using the EORTC QLQ-C30 and CR29 scales.<br>**Safety/Tolerability evaluation**<br>Descriptive statistical analysis is performed for various types of adverse events and serious adverse events that occur in this study. Laboratory test results describe the conditions that are normal before the test but abnormal after treatment and the relationship with the study drug in case of abnormal changes.<br>**Exploratory evaluation indicators**<br>Descriptive statistical analysis will be performed on the exploratory biomarkers collected in this study.

List of acronyms and related terms

Abbreviation	Definition
ADA	Anti-drug antibody
AE	Adverse Events
AESI	Adverse events of special interest
APTT	Activated partial thrombin time
ALP	Alkaline phosphatase
ALT	Alanine Aminotransferase
ARDS	Acute respiratory distress syndrome
AST	Aspartate aminotransferase
BICR	Blinded Independent Central Review
C1D1	Cycle 1 Day 1
C2D1	Cycle 2 Day 1
C3D1	Cycle 3 Day 1
NMPA	National Drug Administration
CI	Confidence interval
CRO	Contract research organization
CT	Computed tomography
CTCAE	Criteria for Common Adverse Events
CTL	Cytotoxic T lymphocytes
CTLA-4	Cytotoxic T lymphocyte associated antigen -4
DCR	Disease control rate
RFS	Disease-free survival
RFS_{12}	12 Month Disease-free survival
RFS_{24}	24 Month Disease-free survival
DLT	Dose limiting toxicity
DoR	Duration of response
EC	Ethics Committee
ECG	Electrocardiogram
ECOG	Eastern Cooperative Oncology Group
EFS	Event-free survival
CRF	Case Report Form
ESMO	European Society of Clinical Oncology
FAAN	Food Allergy and Allergy Network
FAS	Full Analysis Set
FDA	US Food and Drug Administration
FT3	Free triiodothyronine
FT4	Free thyroxine
GCP	Good Clinical Practice
HBcAb	Hepatitis B core antibody
HBeAb	Hepatitis B Virus e Antibody

Abbreviation	Definition
HBeAg	Hepatitis B e antigen
HBsAb	Hepatitis B surface antibody
HBsAg	Hepatitis B surface antigen
HBV	Hepatitis B Virus
HBV DNA	Hepatitis B Virus Deoxyribonucleic Acid
HCC	Hepatocellular carcinoma
HCV	Hepatitis C Virus
HCV RNA	HCV ribonucleic acid
HDV	Hepatitis D Virus
HIV	Human immunodeficiency virus
ICC	Intrahepatic cholangiocarcinoma
ICF	Informed Consent Form
ICH	International Conference on Harmonisation
IFN-γ	Interferon -γ
INR	International normalized ratio
IrAE	Immune-related adverse events
IrRECIST	Immune-related Response Criteria in Solid Tumors
LPLV	Last Patient Last Visit
LVEF	Left ventricular ejection fraction
MedDRA	Medical Dictionary for Regulatory Activities
MSI	Microsatellite instability
MRI	Magnetic resonance imaging
MVI	Microvascular invasion
NIAID	National Institute of Allergy and Infectious Diseases
NOAEL	No observed adverse effect level
NSAID	Nonsteroidal anti-inflammatory drugs
ORR	Objective response rate
OS	Overall survival
PCR	Polymerase chain reaction
PCR	Complete response rate
PD-1	Programmed death receptor -1
PD-L1	Programmed death ligand -1
PK	Pharmacokinetics
PR	Partial response
PT	Prothrombin time
Q2W	1 every 2 weeks
RECIST	Response Evaluation Criteria in Solid Tumors
SAE	Serious Adverse Events
SD	Stable disease
SOP	Standard Operating Procedure
SS	Safety Analysis Set

**Abbreviation**	**Definition**
TACE	Percutaneous transhepatic arterial chemoembolization
TB	Tuberculosis
TGR	Tumor regression grade
TIL	Tumor-infiltrating lymphocytes
TSH	Thyrotropin
T3	Total triiodothyronine
T4	Total thyroxine
TT	Thrombin time
TTP	Time to Progression
ULN	Upper limit of normal

1. Study background

1.1 Colorectal Cancer-Related Background

Colorectal cancer is one of the most common malignant tumors in China. In 2012, there were 253,000 new cases of colorectal cancer in China, accounting for 18.6% worldwide. And morbidity and mortality are on the rise. According to the 2015 China Cancer Statistics, the incidence and mortality of colorectal cancer in China rank the fifth among all malignant tumors, with 376,000 new cases and 1.91 million deaths 1 . Because of the lack of specific symptoms in their early lesions, 70 to 80% of patients have locally advanced disease at the time of clinical diagnosis, including 30 to 40% of stage III patients. Most patients are found in the middle and advanced stages.

1.1.1 Treatment of locally advanced rectal cancer

According to the 2017 CSCO Guidelines for the Diagnosis and Treatment of Colorectal Cancer [1], the treatment strategy of preoperative concurrent chemoradiotherapy + surgery + adjuvant chemotherapy remains the standard treatment strategy for middle and low locally advanced rectal cancer (stage II, III) due to the high risk of local recurrence. Study AIO-94, a Phase 3 controlled study comparing preoperative and postoperative concurrent chemoradiotherapy in patients with stage II/III rectal cancer, showed a significant reduction in local recurrence with neoadjuvant chemoradiotherapy (6% vs 13%, P = .006), a reduction in treatment-related toxicity (27% vs 40%, P = .001), and no difference in overall survival [2]. A meta-analysis of 5 randomized controlled studies showed that the pCR rate (ypT0N0) was significantly higher in the preoperative chemoradiotherapy group than in the preoperative radiotherapy alone group in patients with resectable stage II/III rectal cancer (11.8% vs 3.5%, OR 3.52, 95% CI 2.12-5.84, P < 0.00001) [3]. NSABP R-04 was a randomized phase 3 X 2 study design comparing 5-FU or capecitabine, alone or in combination with oxaliplatin in patients with stage II/III rectal cancer to compare the efficacy and safety, DFS and OS, local recurrence, and pCR rates between different neoadjuvant chemoradiation regimens, with pCR rates ranging from 17.8% to 20.7% [4]. FOWARC was a Chinese multicenter, randomized, open-label, phase III trial comparing the efficacy and safety of mFOLFOX6 or fluorouracil combined with radiotherapy and mFOLFOX6 alone as neoadjuvant therapy for locally advanced rectal cancer, and the preliminary results showed that in the pathological complete response rate (pCR) was 14.0%, 27.5%, and 6.6%, and the downstaging (ypStage 0 to 1) reached 37.1%, 56.4%, and 35.5%, respectively in the fluorouracil chemoradiotherapy group, the mFOLFOX6 chemoradiotherapy group and the mFOLFOX6 group [5] . Although radiotherapy/chemoradiotherapy can reduce local recurrence of rectal cancer, it is also accompanied by increased toxicity, including radiation-mediated toxicity, such as radiation enteritis, cystitis, reproductive dysfunction, hematological and

non-hematological toxicities. A phase 2 study evaluated capecitabine, oxaliplatin, bevacizumab combined with neoadjuvant chemoradiotherapy in T3/T4 rectal cancer, with a pCR rate of 17%, but chemoradiotherapy toxicity and surgical toxicity were evident and led to decreased treatment compliance in the postoperative adjuvant phase [6]. In addition, several randomized phase 2 and 3 studies have explored different chemotherapy regimens with more intensive chemotherapy combined with targeted agents, and the pCR remains between 15% and 20%, with no significant difference in DFS, OS, or local recurrence between the groups, and higher toxicity and more postoperative complications are observed [7-8].

1.1.2 Treatment of locally advanced colon cancer

According to the 2017 CSCO Guidelines for the Diagnosis and Treatment of Colorectal Cancer 1, resection of the corresponding intestinal segment of the colon plus regional lymph node dissection is the treatment strategy for T2-4 colon cancer. For patients with preoperative imaging reporting T4 colon cancer, neoadjuvant chemotherapy followed by colectomy is feasible under the premise of multidisciplinary discussion, and chemotherapy regimens with high objective response rate or chemotherapy combined with targeted therapy are recommended. For patients with high-risk factors in stage II, such as poor histological differentiation (grade 3-4), T4, angiolymphatic invasion, preoperative intestinal obstruction or intestinal perforation, insufficient lymph nodes detected in surgical specimens (less than 12), and stage III, adjuvant chemotherapy is recommended to be started about 4 weeks after surgery, and the duration of chemotherapy is 3-6 months. CAPEOX and FOLFOX or capecitabine alone are recommended for chemotherapy regimens.

1.1.3 Treatment for Colorectal Cancer with MSI-H (Microsatellite Instability-high) or dMMR (Mismatch Repair Deficiency)

The patients with MSI-H or dMMR accounts for 10-15% of all patients with colorectal cancers, compared with only 5% of patients with metastatic disease 9. Deficiency of DNA mismatch repair (MMR) can be detected by immunohistochemical staining lacking MMR proteins MLH1, MSH2, MSH6, or PMS2 or by identifying changes in microsatellite length between patient tumors and normal tissue or blood samples by PCR. Next generation sequencing of tumors has shown accurate detection of microsatellite instability (MSI) status (mSINGS29, MSIsensor30, 31, and MOSAIC32) over the past 5 years. Lynch syndrome patients are characterized with MSI-H. Besides, other mechanisms, such as somatic mutations in the MMR genes and hypermethylation of the MLH1 promoter will result in sporadic MSI-H tumors. Compared to other types of CRC, immune cells are clearly infiltrated in the dMMR/MSI-H subtype, particularly CD8+ tumor-infiltrating lymphocytes (TILs), T helper 1 (TH1) CD4+ TILs macrophages, and have a type I interferon-rich

microenvironment. Therefore, it is considered to be an immunogenic tumor and is a hot spot for anti-tumor immunotherapy.

In 2015, Le et. al. conducted a phase II study of pembrolizumab in refractory metastatic cancers with a total of 41 patients. In colorectal cancer patients with dMMR/MSI-H versus normal mismatch repair (pMMR), the immune-related objective response rate (irORR) was 40% vs 0%, and immune-related PFS (irPFS) was 78% vs 11%. The median OS and PFS of the former were not reached, and the latter were 5 months and 2.2 months, respectively 10. Whole genome sequencing showed that patients with dMMR had an average of 1782 somatic mutations per tumor, while patients with pMMR had only 73 somatic mutations, and a high somatic mutation burden was significantly associated with prolonged PFS. For patients with metastatic colorectal cancer who have received second-line therapy with dMMR, 74 patients with dMMR-MSI-H advanced CRC were treated with nivolumab in CheckMate 142 trial. Investigator-assessed objective responses were obtained in 23 (31%) patients and disease control ⩾ 12 weeks was observed in 51 (69%) patients. Median PFS was 14.3 months (95%CI 4.3 months, NA) and 12-month PFS was 50% (95% CI 38-61%). Overall survival at 12 months was 73% (95% CI 62-82%) 11-12. The combination of nivolumab and ipilimumab was also assessed in this trial with an ORR of 55%, 1-year PFS of 71%, and OS of 85% 11. Combined nivolumab and ipilimumab treatment resulted in an increased incidence of drug-related immune-related adverse events, with 32% of patients experiencing grade 3-4 treatment-related adverse events compared with 20% of patients treated with nivolumab alone. Based on the compelling data on immunotherapy in dMMR/MSI-H CRC, the FDA approved pembrolizumab in May 2017 and nivolumab in the second-line treatment of patients with dMMR/MSI-H CRC in July 2017. So far, no drugs have been approved by the European Medicines Agency (EMA), awaiting the results of a phase III randomized controlled study.

Many studies are currently ongoing to assess the efficacy and safety of PD1 or PDL1 blockade in patients with dMMR/MSI-H CRC. Keynote 177, for example, is a randomized controlled phase 3 study to assess the efficacy and safety of pembrolizumab versus standard of care in treatment-naïve dMMR/MSI-H CRC. A randomized controlled phase 3 study (NCT02997228) assessed the efficacy and safety of atezolizumab or atezolizumab in combination with FOLFOX plus bevacizumab versus FOLFOX plus bevacizumab in treatment-naïve dMMR/MSI-H CRC. Another randomized controlled phase 3 study (NCT02912559) assessed the efficacy and safety of atezolizumab in combination with comparator FOLFOX as adjuvant therapy for dMMR/MSI-H CRC.

Preliminary results from a single-arm study of short-term combined nivolumab and ipilimumab in patients with resectable CRC presented at the 2018 ESMO meeting were encouraging 13 . The

primary endpoint of this study was safety and feasibility, and secondary endpoints included pathological response. All patients underwent surgery up to 6 weeks after informed consent. All 7 patients with dMMR-MSI-H tumors had a major pathologic response, with 4 (57%) having a complete response. In eight patients with pMMR/MSI-L tumors, no major pathologic response was identified, but T-cell infiltration was significantly increased after treatments in both dMMR/MSI-H (P = 0.0009) and pMMR/MSI-L population (P = 0.018).

1.2 Background of Immunotherapy

Programmed death-1 (PD-1) is an inhibitory receptor of the immunoglobulin family on the surface of activated T lymphocytes whose ligands are B7 homologous protein programmed death-1 (PD-L1) (also called B7-H1) and programmed death-2 (PD-L2) (also called B7-DC). PD-1/PD-L1 binding plays an important role in down-regulating T cell activation and maintaining peripheral immune tolerance, so tumor cells inhibit T cell activation by expressing PD-L1 and then interacting with PD-1 to evade the killing of immune cells. Blocking this immune checkpoint can enhance the proliferation, survival and killing activity of T cells and achieve the effect of cancer immunotherapy.

Pembrolizumab, developed by Merck Sharp & Dohme in 2014, is the first PD-1 inhibitor and was approved by the US Food and Drug Administration (FDA) in 2014 for the treatment of advanced or unresected melanoma, with indications subsequently approved for non-small cell lung cancer, renal cell carcinoma, classical Hodgkin lymphoma, head and neck cancer, bladder cancer (urothelial carcinoma), gastric cancer, liver cancer, Merkel cell skin cancer, solid tumors carrying MSI-H or dMMR, and MSI-H/dMMR colorectal cancer (see Section 1.1.3) . Nivolumab, developed by Bristol-Myers Squibb, is the second PD-1 inhibitor that was approved by the FDA in 2015 for the treatment of metastatic melanoma and subsequently for the treatment of non-small cell lung cancer, renal cell carcinoma, classical Hodgkin lymphoma, head and neck squamous cell carcinoma, urothelial carcinoma, MSI-H cancer, and HCC. In addition, multiple PD-1/PD-L1 inhibitors have been approved in multiple cancers, including toripalimab (JS001), which was conditionally approved by China National Medical Products Administration on 17 December 2018 for the treatment of locally advanced or metastatic melanoma after failure of previous standard therapies.

PD-1/PD-L1 blockade combined with chemotherapy has achieved superior clinical efficacy in multiple cancers and has now been approved in non-small cell lung cancer, small cell lung cancer, and triple-negative breast cancer. For example, KEYNOTE 189 was a randomized controlled phase 3 study comparing pembrolizumab in combination with pemetrexed versus pemetrexed in the treatment of untreated locally advanced or metastatic non-small cell lung cancer 14. The study

randomized 616 patients according to a ratio of 2:1. After a median follow-up of 10.5 months, the 12-month survival rate was 69.2% (95% confidence interval [CI], 64.1-73.8) in the pembrolizumab combination group and 49.4% (95% CI, 42.1-56.2) in the placebo group, with an OS hazard ratio of 0.49 (95% CI, 0.38 to 0.64; P < 0.001). Improvements in overall survival were observed across all PD-L1 categories assessed. Median progression-free survival was 8.8 months (95% CI, 7.6-9.2) in the combined group and 4.9 months (95% CI, 4.7-5.5) in the placebo group, with a PFS hazard ratio of 0.52 (95% CI, 0.43-0.64; P < 0.001). The combined group had an objective response rate of 47.6% (95% CI, 42.6-52.5) compared with 18.9% (95% CI, 13.8-25.0) in the control group. Grade 3 or higher adverse events occurred in 67.2% of patients in the combined group and 65.8% of patients in the placebo group. The combination therapy was generally well tolerated and efficacy was independent of PD-L1 expression in the tumor, and even PD-L1-negative patients could benefit from the combination therapy, suggesting that inhibiting the PD-L1/PD-1 signaling pathway together with activated immune system with high levels of tumor antigens resulted from tumor cell killed by cytotoxic chemotherapy can restore tumor-specific T-cell immunity and may result in deeper and durable responses than standard chemotherapy alone [15].

A synergistic effect was also observed with PD-1/PD-L1 antibody in combination with VEGFR pathway drugs, such as interim results presented at the 2018 ESMO meeting in a phase 1 study of atezolizumab in combination with bevacizumab in advanced HCC [16], which showed a response rate of 32% (23/73), among which 52% of patients responded for 6 months or longer, 26% responded for 12 months or longer. Grade 3-4 treatment-related adverse event rate was 27% (28/103), with hypertension being most common adverse event (10%, 10/103). The safety of this combination therapy was tolerable and controllable. A global phase 3 clinical trial (NCT03434379) is currently ongoing to compare the efficacy of combination therapy with sorafenib monotherapy in first-line setting in advanced HCC.

In the field of neoadjuvant therapy, PD-1/PD-L1 blockade has achieved preliminary clinical results in multiple cancer types, including preliminary results of nivolumab as neoadjuvant therapy in resectable non-small cell lung cancer published in the New England Journal of Medicine (NEJM) in April 2018. In this Phase 1b study, 21 patients received two doses of nivolumab (3 mg/kg, every two weeks) before surgery and surgery was scheduled 4 weeks later. Positive treatment efficacy was observed in the trial. Radical surgery was achieved in > 95% (20/21) of patients, with a major pathological response rate of 45% (9/20) (95% CI 23-68%), and imaging assessment according to RECIST 1.1 revealed partial response in 10% (2/21) of patients and stable disease in 86% (18/21) of patients. Adverse events were generally consistent with most other studies of nivolumab with no other safety concerns [17]. In 2018 ASCO meeting, the results of a phase 2 study of single dose of

pembrolizumab 200 mg as neoadjuvant therapy for head and neck squamous cell carcinoma were reported, with a pCR rate of 50% [18]. Another neoadjuvant therapy of atezolizumab for muscle-invasive bladder cancer reported a pCR rate of 29% [19]. These early-phase studies showed that immunotherapy has achieved promising efficacy in multiple neoadjuvant therapies for solid tumors with acceptable feasibility and safety.

Based on the clinical data and clinical development progress of PD-1/PD-L1 blockade in dMMR/MSI-H CRC, as well as significant clinical benefits of neoadjuvant immunotherapy in NSCLC, head and neck squamous cell carcinoma and bladder cancer, it is worthy to investigate the efficacy and safety of JS001 in the neoadjuvant setting in locally advanced dMMR/MSI-H rectal cancer and colon cancer, and to explore whether PD-1/PD-L1 blockade combined with chemotherapy and bevacizumab can improve the immune microenvironment, activate tumor immunity, reduce tumor volume, achieve pathological response, reduce the complexity of surgery, improve the radical effect of surgery, thereby delaying the recurrence of the disease and prolonging the survival of patients.

1.3 Toripalimab (JS001) Drug Background

Toripalimab (hereinafter referred to as JS001), a recombinant humanized anti-PD-1 monoclonal antibody independently developed by Shanghai Junshi Bio-pharmaceutical Technology Co., Ltd., is a novel recombinant humanized anti-PD-1 monoclonal antibody belonging to the human IgG4/Kappa subtype, which binds to PD-1 with high affinity and selectively blocks the binding of PD-1 to its ligands PD-L1 and PD-L2, thereby activating T lymphocytes, improving lymphocyte proliferation and cytokine secretion, especially interferon-γ (IFN-γ).

Toripalimab (JS001) has been conditionally approved by China National Medical Products Administration on 12 17 2018 for the treatment of locally advanced or metastatic melanoma after failure of prior standard therapy.

1.3.1 Preclinical studies

1.3.1.1 Pharmacodynamics

In vitro pharmacodynamic studies showed that JS001 significantly stimulated T cell proliferation and promoted IFN-γ release. The results of in vivo experiments in NSG mice showed that JS001 could effectively increase the proliferation of human CD4 + and CD8 + T cells in vivo, and better promote the activation of human effector/memory T cells, and its stimulation effect was significantly stronger than that of nivolumab, a similar target drug marketed abroad. JS001 abrogated

tumor immune suppression of T cells in vivo, and cytotoxic T lymphocytes (CTL) killed tumor cells. In addition, JS001 did not cause antibody-dependent cell-mediated cytotoxicity and cytokine storm.

Receptor occupancy assay in cynomolgus monkeys showed a dose-response relationship between JS001 binding to PD-1 on the cell surface, and the effective dose started at an antibody serum concentration of 0.3 μg/mL and reached saturation at 3 μg/mL, exerting the best biological effect. These preclinical data provide a basis for dose extrapolation to support further clinical studies with JS001.

1.3.1.2 Pharmacokinetics/Pharmacodynamics and Immunogenicity

Following a single intravenous infusion of different doses (1, 10, 75 mg/kg) of the test drug JS001 in cynomolgus monkeys, serum drug exposure levels basically increased linearly with dose over the dose range of 1 to 10 mg/kg. C max in each dose group was 27.70 ± 12.29 μg/mL, 216.11 ± 34.52 μg/mL, and 1891.72 ± 270.16 μg/mL, respectively. Its in vivo t 1/2 ranged from 134 to 194 hours, which was similar to the reported pharmacokinetic (PK) data for nivolumab and pembrolizumab, and no significant immunogenicity was found.

1.3.1.3 Toxicology

Because JS001 did not specifically bind to murine PD-1, cynomolgus monkeys were selected as the main test animals in toxicology studies referring to the Guidelines for Nonclinical Safety Evaluation of Therapeutic Biological Products. National (Shanghai) Center for Safety Evaluation and Research of New Drugs was entrusted to conduct systematic preclinical safety toxicological study and evaluation in strict accordance with the requirements of Good Laboratory Practice for Non-Clinical Laboratory Studies. General pharmacology, acute toxicity, long-term toxicity testing, hemolysis and local irritation and other toxicity studies were performed.

In the safety pharmacology evaluation study of JS001, intravenous injection of JS001 at doses of 10, 30 and 100 mg/kg in cynomolgus monkeys had no significant effect on the cardiovascular system and respiratory rate. Meanwhile, JS001 had no effect on motor coordination and behavioral activity in rats.

The acute toxicity test showed that a single intravenous injection of JS001 in cynomolgus monkeys showed no significant acute toxic effects, and the no-observed-adverse-effect level (NOAEL) was 406 mg/kg.

In the subacute toxicity test of repeated intravenous injection of JS001 once a week for four consecutive weeks, no significant toxic side effects were observed in JS001. No treatment-related abnormal reactions were observed in any treated animal. No regular changes of toxicological significance were observed in body weight, body temperature, electrocardiogram (ECG) parameters,

coagulation function, ophthalmic examination, urine routine distribution, serum cytokines and various blood biochemical indicators at each test time point in each dose group. JS001 exhibited linear kinetics in cynomolgus monkeys and accumulation after repeated dosing.

During the long-term toxicity test of JS001 at doses of 10, 30 and 100 mg/kg in cynomolgus monkeys intravenously injected for 26 consecutive weeks, no JS001-related abnormal changes in body weight, food consumption, clinical observation, body temperature, ECG, ophthalmology, blood routine and coagulation function, serum biochemistry, immune function, urine and fecal examination, gross pathology, organ weight, and histopathology showed no significant toxic reactions. Therefore, the NOAEL for JS001 was 100 mg/kg.

Detailed information about JS001 preclinical studies can be found in the JS001 Investigator 's Brochure.

1.3.2 Clinical safety

Safety was summarized for 598 patients from different tumor types, including melanoma, nasopharyngeal carcinoma, esophageal squamous cell carcinoma, gastric adenocarcinoma, head and neck squamous cell carcinoma, NSCLC, triple-negative breast cancer, malignant lymphoma, soft tissue sarcoma, etc.

576 (96.3%) of 598 patients experienced at least 1 AE and 556 (93.0%) patients experienced AEs related to the study. The most frequently reported (⩾ 10%) study drug-related adverse events were anemia, alanine aminotransferase increased, aspartate aminotransferase increased, pyrexia, cough, white blood cell count decreased, rash, decreased appetite, asthenia, and hypothyroidism (the causal relationship of this adverse event/serious adverse event will be classified as related to study drug if the investigator judges it to be definitely related, probably related, possibly related, or unlikely related). Eighty-eight (14.7%) patients experienced serious adverse events related to the study drug: pulmonary infection 10 (1.7%), death 8 (1.3%), and pneumonia 6 (1.0%), platelet count decreased 5 (0.8%). 78 (13.0%) patients experienced study drug-related adverse events leading to permanent discontinuation of trial medication. Fifty-two (8.7%) patients experienced adverse events related to study drug that led to interruption of trial medication. 142 (23.7%) patients experienced study drug-related adverse events of grade 3 and higher, of which ⩾ 4 (0.7%) events observed were anemia 16 (2.7%), hyponatremia 16 (2.7%), lipase increased 9 (1.5%), death 8 (1.3%), lung infection 7 (1.2%), amylase increased 6 (1.0%), aspartate aminotransferase increased 6 (1.0%), alanine aminotransferase increased 5 (0.8%), platelet count decreased 4 (0.7%), blood triglycerides increased 4 (0.7%), γ - glutamyltransferase increased 4 (0.7%), hypokalemia 4 (0.7%), hyperuricemia 4 (0.7%), fatigue 4 (0.7%), hypertension 4 (0.7%), pneumonia 4 (0.7%). 155 (25.9%)

cases of immune-related adverse events were observed, manifested as immune-related interstitial lung disease, immune-related hypothyroidism and hyperthyroidism, immune-related pancreatitis, immune-related hyperglycemia or diabetes, immune-related liver dysfunction, immune-related adrenocortical insufficiency. Most of these immune-related adverse events were grade 1-2, mostly did not lead to termination or interruption of the investigational product, and most of them were in complete remission. These adverse events were consistent with immune-related adverse events observed in other similar products, and no new immune-related adverse events were found.

Please refer to the Investigator 's Brochure for details.

1.3.3 Clinical efficacy

HMO-JS001-II-CRP (CT4) trial was an open-label, multicenter, single-arm, phase 2 clinical study to investigate the efficacy and safety of JS001 3 mg/kg Q2W in patients with locally advanced or metastatic melanoma after failure of standard therapy, and the primary endpoint was objective response rate (ORR) assessed by an independent imaging review committee based on Response Evaluation Criteria in Solid Tumors (RECIST1.1), As of 15 March 2018 , 128 patients with locally advanced or metastatic melanoma who had failed previous treatment were enrolled and evaluated by an independent evaluation committee according to the RECIST1.1, in the evaluable population, 1 patients (0.83%) had a complete response (CR), 21 (16.54%) partial response, 51 (40.16%) stable disease and 48 (37.80%) progressive disease. ORR was 17.32% (95% CI :11.19-25.04%), and the disease control rate (DCR) was 57.48% (95% CI: 48.40-66.20%). Duration of response (DOR) analysis showed that 22 patients CR or PR had 21 ongoing responses as of the data cutoff date and the median duration of response had not been reached. DOR ranged from a minimum of 1.87 months to a maximum of 10 months, all of which were censored data. Analysis of time to response (TTR) showed that 22 of 127 patients had CR or PR, with a median TTR of 3.45 months, 95% CI (1.74-3.57), with 1.61 months being the minimum and 7.34 months being the maximum. Median progression-free survival was 3.61 months (95% CI 2.72-5.48 months), the longest progression-free survival was 13.61 months, and 6 months progression-free survival rate was 34.89%. OS data are far from mature (107 censored among 127 patients), and the median overall survival has not yet been reached, with the longest survival of 14.52 months, 6 months survival rate of 88.87%, and 12-months survival rate of 78.97%.

JS001-Ib-CRP-1.0 (CT5) trial is a multi-cohort phase Ib/II clinical study (basket trial) to preliminarily evaluate the anti-tumor activity and tolerability of JS001 in the treatment of advanced gastric adenocarcinoma, esophageal squamous cell carcinoma, nasopharyngeal carcinoma and head and neck squamous cell carcinoma, providing a basis for subsequent phase 3 clinical studies. The

primary endpoint was objective response rate as assessed by RECIST1.1. An interim analysis as of 30, November 2017 showed 1 complete response (esophageal cancer) and 35 partial responses with an objective response rate of 22.4% in evaluable population as evaluated by the investigator according to RECIST1.1. 44 patients (27.3%) in the evaluable population had stable disease, and the disease control rate was 49.7%. 8 patients with gastric adenocarcinoma had partial responses, with objective response rate 20.0%. 1 patient with esophageal squamous cell carcinoma had complete response, 9 patients had partial responses, with objective response rate of 20.8%. 14 patients with nasopharyngeal carcinoma had partial responses, with objective response rate of 28.0%. 4 patients with head and neck squamous cell carcinoma had partial response, with objective response rate of 17.4%.

Please refer to the Investigator 's Brochure for details.

1.3.4 Clinical Pharmacokinetics, Dose Selection, and Immunogenicity

Preliminary PK data (0.3 to 10 mg/kg) showed that JS001 basically exhibited linear PK profiles over this dose range. The drug exhibited some nonlinear PK profiles when the dose was increased to 10 mg/kg. After continuous intravenous drip of JS001 for about 3 ~ 4 times in clinical patients, the plasma concentration basically reached steady state. In vitro experiments showed that PD-1 receptor on the surface of T cells could be saturated when JS001 concentration > 20 nM or 3 μg/mL. Considering the limited entry of antibody macromolecules into the tumor microenvironment, JS001 in peripheral blood was generally maintained at 25 μg/mL. Steady-state minimum plasma concentrations were approximately 20 to 40 μg/mL at 3 mg/kg every 2 weeks. In addition, each phase 1 study showed that complete PD- 1 receptor occupancy could be maintained throughout the treatment period at different dose groups (0.3, 1, 3, 10 mg/kg, biweekly dosing). Based on the above pharmacokinetic study results as well as data on receptor occupancy, and the 3 mg/kg dose every 2 weeks was selected for the CT4 Phase 2 pivotal study.

360 mg fixed dose administered every 3 weeks was explored in two earlier studies (CT5 and CT7), in which preliminary pharmacokinetics derived from 9 patients showed that compared to 3mg/kg every two weeks (11 patients derived from CT1), steady-state peak concentrations increased 153% (166.53 ug/mL, 95%CI 121.0-212.0 vs 108.83 ug/mL, 95% CI 87.5-130.0), steady-state peak concentration increased 133%(47.92 ug/mL, 95% CI 31.8-64.0 vs 36.09 ug/mL, 95% CI 27.7-44.5), AUC 0-Day85 increased 138% (164,993 ug/mL*hr vs 119814 ug/mL*hr). Based on PK model prediction, trough concentration (~ 32 ug/mL) and drug exposure was similar between 240mg every 3 weeks and 3mg/kg every two weeks. Taken together, the results from the pivotal JS001

pharmacokinetic, receptor occupancy, and phase 2 melanoma studies supported JS001 240 mg as a fixed dose administered every 3 weeks as the recommended dose for phase 3 clinical studies.

In addition, the pharmacokinetic data in single and multiple dose phases of CT1 showed that JS001 accumulated in humans to some extent after multiple doses. No dose-limiting toxicity was observed from 0.3 mg/kg to 10 mg/kg in previous escalation studies and JS001 was well tolerated. Combined with the above information, JS001 was administered at a fixed dose of 240mg, which was recommended for phase 3 clinical studies.

The use of any recombinant protein has the potential to induce local and systemic immune responses. Patients should be carefully monitored for anti-drug antibodies (ADAs) using additional tests, such as autoimmune serology or biopsy, to determine the probability of possible immunogenicity and impact on the patient.

The results of the present pooled analysis based on 518 patients showed that ADAs were observed in all patients treated with 0.3-10 mg/kg, and the individual ADA positive rate of patients was 17.2%. Individual ADA positive rate 18% was observed in 128 melanoma patients who received toripalimab 3 mg/kg. According to the pharmacokinetic and exposure - response analyses, no evidence of loss of efficacy, changes in toxicity profile, or changes in pharmacokinetic profile was observed in the presence of anti-drug antibodies, and no differences in efficacy and safety were observed between ADA -positive and -negative patients.

Please refer to the Investigator 's Brochure for details.

1.4 Study rationale

Because of the high risk of local recurrence, preoperative concurrent chemoradiotherapy + surgery + adjuvant chemotherapy remains the standard treatment strategy for middle and low locally advanced rectal cancer (II, III). Although radiotherapy/chemoradiotherapy can reduce local recurrence of rectal cancer, it is also accompanied by increased toxicity, including radiation-mediated toxicity, such as radiation enteritis, cystitis, reproductive dysfunction, hematological and non-hematological toxicity. Colon cancer staged as T4 on preoperative imaging also carries a high risk of local recurrence, and current guidelines recommend neoadjuvant chemotherapy followed by colectomy under multidisciplinary discussion. The efficacy of current combined chemoradiotherapy with more intensive chemotherapy and targeted agents did not significantly improve the pCR rate, pCR remained between 15% and 20%, DFS, OS, and local recurrence were not significantly different between the groups, and higher toxicity and more postoperative complications were observed.

In the meantime, immunotherapy has made major breakthroughs in the treatment of advanced dMMR/MSI-H CRC. Compared with other types of CRC, dMMR/MSI-H subtype was characterized with highly infiltrated immune cells, particularly CD8 + tumor-infiltrating lymphocytes (TILs), T helper 1 (TH1) CD4 + TILs macrophages, and type I interferon-rich microenvironment. It was therefore considered a kind of immunogenic tumor. PD-1 blockade monotherapy has a response rate of 31% to 40%, and FDA has now approved pembrolizumab and nivolumab for second-line treatment of dMMR/MSI-H CRC patients.

PD-1/PD-L1 blockade has achieved significant preliminary clinical efficacy in neoadjuvant therapy in NSCLC, head and neck squamous cell carcinoma and bladder cancer, with pCR rates of 30-50%, and phase 3 clinical trials are currently ongoing. The results of a phase 1 study reported at the ESMO meeting 2018 showed that short-term neoadjuvant therapy with nivolumab and ipilimumab achieved major pathological responses in all 7 patients with dMMR/MSI-H CRC, with 4 (57%) having complete responses. In 8 patients with pMMR/MSI-L CRC, no major pathologic response was observed. And T cell infiltration was significantly increased after treatment in both dMMR/MSI-H ($P = 0.0009$) and pMMR/MSI-L population ($P = 0.018$). These results suggest that immune neoadjuvant therapy has encouraging promise in patients with dMMR/MSI-H CRC.

PD-1/PD-L1 blockade combined with chemotherapy and anti-VEGFR treatmetn achieved superior clinical efficacy in multiple cancers, suggesting that inhibiting the PD-L1/PD-1 signaling pathway together with activated immune system with high levels of tumor antigens resulted from tumor cell killed by cytotoxic chemotherapy can restore tumor-specific T-cell immunity and may result in deeper and durable responses than standard chemotherapy alone.

At present, the efficacy of concurrent chemoradiotherapy + surgery + adjuvant chemotherapy, a standard treatment strategy for middle and low locally advanced rectal cancer (II, III), remains limited. There is no neoadjuvant treatment result of immunotherapy combined with chemotherapy and bevacizumab for CRC patients with MSI-H/dMMR, which is an unmet clinical need. Based on the clinical data of the above similar products in the population carrying MSI-H/dMMR , as well as NSCLC, significant clinical benefits of neoadjuvant therapy in the areas of head and neck squamous cell carcinoma and bladder cancer support further exploration of the feasibility, safety and preliminary efficacy of JS001 as neoadjuvant therapy in colorectal cancer patients carrying MSI-H/dMMR, and it is necessary to observe whether PD-1/PD-L1 pathway blockers combined with chemotherapy and bevacizumab can improve the immune microenvironment, intensify tumor immunity, reduce tumor size, achieve pathological response, reduce the complexity of surgery, and improve the radical effect of surgery, thereby delaying the recurrence of the disease and prolonging the survival of patients. Thus, it is of great clinical significance to understand whether the new

treatment mode has better tolerability and clinical efficacy compared with the traditional chemoradiotherapy treatment mode.

1.5 Benefit - Risk Assessment

As a novel recombinant humanized anti- PD-1 monoclonal antibody, several clinical studies of JS001 are ongoing in China. In terms of safety, DLT has not been observed in each dose group (0.3, 1, 3, 10 mg/kg Q2W, etc.) so far in the phase 1 study of JS001. The 240 mg Q2W dose group has been expanded, which is generally well tolerated, and no DLT has been observed. The most common treatment-related AEs were grade 1-2, which were found to be related to study drug by preliminary analysis。 There was no dose-response relationship between toxicity and drug dose. Most irAEs did not lead to discontinuation or interuption of the investigational product, which were consistent with those observed in other similar products. JS001 was well tolerated, and no new safety signals were identified.

In terms of efficacy, recent clinical study data have shown that the similar drugs nivolumab and pembrolizumab monotherapy have significant benefits and are well tolerated in patients with advanced dMMR/MSI-H CRC, which have been approved for accelerated approval by FDA. These results suggest the antitumor activity of this kind of drugs in patients with dMMR/MSI-H CRC. In the field of neoadjuvant therapy, PD-1 antibodies have achieved significant pathological response rates in early clinical studies as neoadjuvant therapy in NSCLC, head and neck squamous cell carcinoma, bladder cancer and other fields except dMMR/MSI-H CRC. In addition, significant preliminary clinical benefit and good tolerability of JS001 in combination with standard chemotherapy for the treatment of non-small cell lung cancer, gastric cancer, and esophageal cancer have been observed in JS001 phase 2 studies. It therefore supports further exploration of the feasibility, safety, and preliminary efficacy of JS001 in combination with chemotherapy and bevacizumab as neoadjuvant therapy in patients with locally advanced dMMR/MSI-H colorectal cancer.

A detailed risk management plan will be developed for this study to ensure that potential AEs are expected to cause minimal harm to patients. At the same time, patients will be closely observed for AEs and irAEs during study drug treatment, and the study doctor will immediately take appropriate measures to ensure the safety of patients in case of relevant adverse events.

2. Study objectives

2.1 Study objectives

Primary objective:

- To evaluate the rate of complete pathological response (pCR) by blinded, independent, central review (BICR) of toripalimab (JS001) combined with bevacizumab and chemotherapy as neoadjuvant therapy MSI-H (microsatellite instability-high) or dMMR (mismatch repair deficiency) for advanced colon cancer;

Study hypothesis 1: Toripalimab (JS001) combined with bevacizumab and chemotherapy as neoadjuvant therapy improves the rate of complete pathological response (pCR) as assessed by blinded independent central pathology in patients with MSI-H/dMMR advanced colon cancer compared with historical control, where the historical control pCR rate is 3%.

- To evaluate the rate of complete pathological response (pCR) with BICR of toripalimab (JS001) combined with bevacizumab and chemotherapy as neoadjuvant therapy in patients with MSI-H or dMMR advanced rectal cancer;

Study hypothesis 2: Toripalimab (JS001) combined with bevacizumab and chemotherapy as neoadjuvant therapy improves the rate of complete pathological response (pCR) as assessed by blinded independent central pathology in patients with MSI-H/dMMR advanced rectal cancer compared with historical controls, where the historical control pCR rate is 10%.

Secondary objectives

- To evaluate the preliminary efficacy of JS001 combined with bevacizumab and chemotherapy as neoadjuvant therapy in patients with MSI-H or dMMR advanced colon cancer: R0 resection rate, time to surgery, pCR rate assessed by pathologists at participating centers, pCR rate assessed by both BICR and pathology at participating centers, tumor regression grade (TGR), objective response rate assessed by investigators (ORR), event-free survival (EFS)), disease-free survival (DFS), 12 months and 24 months disease-free survival (DFS12 , DFS24), 12 months and 24 months survival (OS) rate;
- To evaluate the preliminary efficacy of JS001 combined with bevacizumab and chemotherapy as neoadjuvant therapy in patients with MSI-H or dMMR advanced

rectal cancer: R0 surgery rate, time to surgery, pCR rate assessed by pathologists at participating centers, pCR rate assessed by BICR and pathology at participating centers, tumor regression grade (TGR), objective response rate assessed by investigators (ORR), event-free survival (EFS)), disease-free survival (DFS), 12 months and 24 months disease-free survival (DFS12 , DFS24), 12 months and 24 months survival (OS) rate;

- To evaluate the quality of life of JS001 combined with bevacizumab and chemotherapy as neoadjuvant therapy in patients with MSI-H or dMMR advanced colon and rectal cancer using EORTC QLQ-C30 and CR29 scales;
- To evaluate the safety of JS001 combined with bevacizumab and chemotherapy as neoadjuvant therapy in patients with MSI-H or dMMR advanced colon and rectal cancer;

Exploratory objectives

- To evaluate the correlation between immune-related biomarkers and efficacy in colon and rectal cancer.

2.2 Study Endpoints

The following definitions and specifications for the primary efficacy endpoint, secondary efficacy endpoints, safety endpoints, biomarker endpoints apply to both colon and rectal cancer patient populations.

Primary Efficacy Measures

- Complete pathological response (pCR) rate by blinded independent central pathology assessment

pCR defined as absence of viable cancer cells in any lesion or nodule after neoadjuvant therapy. To reduce bias resulting from assessments, pCR assessments will be performed by blinded independent central pathology assessments. The pCR rate was calculated as the percentage of patients did not achieve pCR among all patients who received neoadjuvant therapy. Patients who did not complete the postoperative pathologic response assessment or who did not undergo surgery were considered non-pCR.

Secondary Efficacy Measures

- R0 resection rate, defined as the percentage of patients who met criteria for R0 resection among all patients receiving neoadjuvant therapy, as judged by the

investigator according to the R0 resection definition.

- Time to surgery, from first neoadjuvant administration to surgery for colon or rectal cancer.
- Rate of complete pathological response (pCR) assessed by the participating site pathologist.

  pCR assessments in this measure will be performed by the participating site pathologist. The pCR rate was calculated as the percentage of patients did not achieve pCR among all patients who received neoadjuvant therapy. Patients who did not complete the postoperative pathologic response assessment or who did not undergo surgery were considered non-pCR.
- pCR rate assessed by both BICR and local investigator
- Tumor regression grade (TGR)
- Investigator-assessed objective response rate (ORR), defined as the best tumor response (complete response or partial response) as assessed by the investigator according to RECIST v1.1 from start of neoadjuvant therapy to the time of colorectal cancer surgery or a tumor assessment (for patients who did not undergo colorectal cancer surgery).
- Event-free survival (EFS), as assessed by the investigator, is defined as the time from the first neoadjuvant therapy with roplerimumab injection (JS001) in combination with bevacizumab and chemotherapy to the first documented inoperable disease progression, local or distant recurrence, or death from any cause, whichever occurs first. EFS assessments were assessed separately by the investigator.
- Disease-free survival (DFS), defined as the time from the date of surgery until the first documented local or distant recurrence or death from any cause, whichever came first.
- 12-month or 24-month disease-free survival (DFS12, DFS24)
- Overall survival (OS), defined as the time from the first dose of neoadjuvant therapy to death from any cause
- Patient quality of life will be measured by cycle using the EORTC QLQ-C30 and CR29 scales.

Safety Endpoints

- Incidence of adverse events, laboratory tests, vital signs, ECG and other safety indicators.

Biomarker Endpoints

- PD-L1, ctDNA, and TMB

3. Study Design

3.1 Overall design

This is a multicenter, open-label, single-arm phase 1b/2 study to enroll patients with MSI-H/dMMR advanced colon and rectal cancer to evaluate the safety and feasibility of toripalimab (JS001) combined with bevacizumab and chemotherapy as neoadjuvant therapy, as well as efficacy indicators, including pathological complete response rate (pCR), ORR, DFS and OS.

To reduce the risk of exposing patients to ineffective treatment regimens, this study will be divided into two stages (Stage I and Stage 2-Extension stage) to enroll patients with colon cancer and rectal cancer. 11 patients with MSI-H/dMMR colon cancer and 8 patients with rectal cancer are planned to be enrolled in stage 1. When 11 patients with colon cancer are enrolled and evaluable for response, a decision will be made whether to continue enrolling patients with colon cancer and possibly to adjust the final number of patients enrolled in this cohort based on the actual response data collected after the end of Stage 1, giving the principle of maximizing the benefit/hazard ratio for patients. The same decision-making process was performed after 8 rectal cancer patients were enrolled. Specific analytical methods and sample sizes are described in Section 11. The study design is detailed in Figure 1

Figure 1 Flow Chart of Study Design

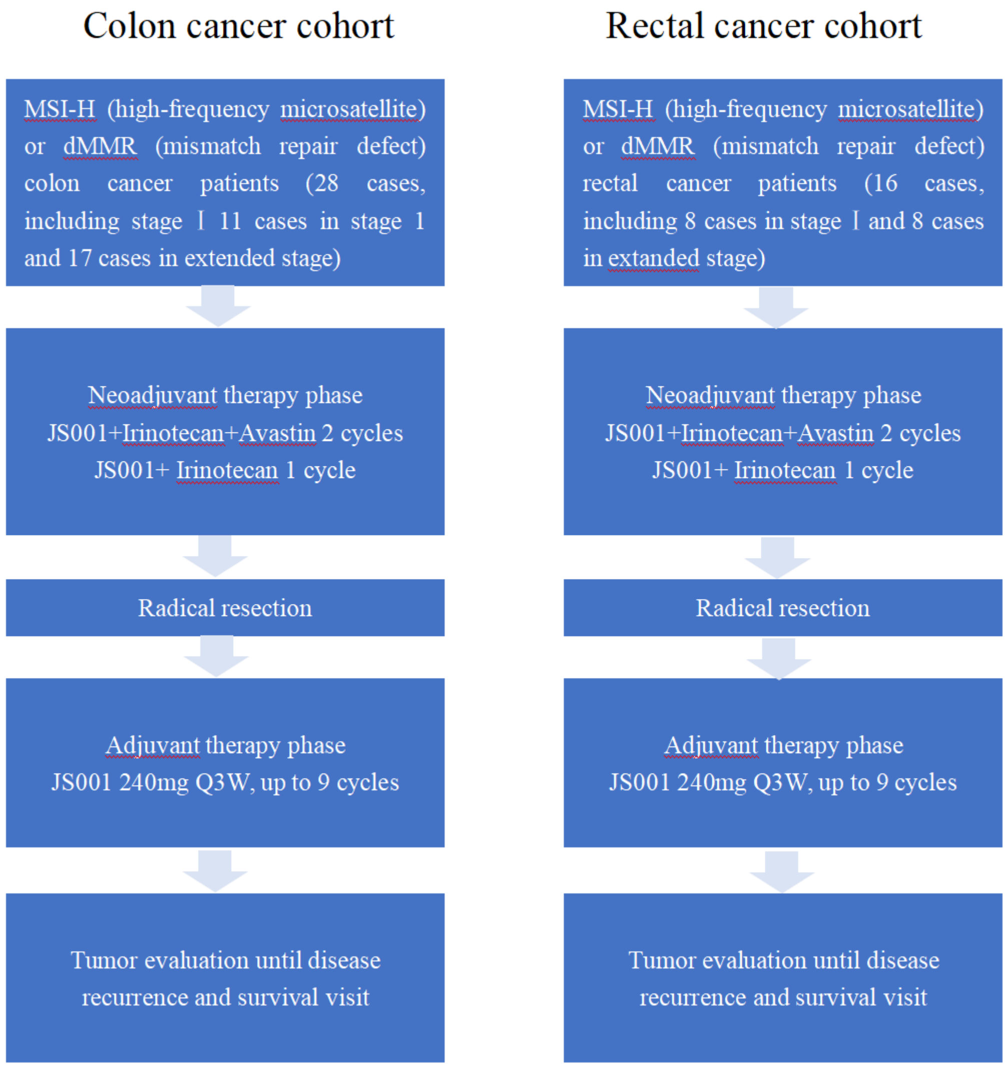

Imaging assessments and laboratory tests were performed during the screening period. According to the staging criteria and treatment roadmap of Chinese Guidelines for the Diagnosis and Treatment of Colorectal Cancer (Ministry of Health 2017), patients with stage T3-T4Nx rectal cancer or stage T1-2 < 12 cm from the anal verge who refused direct surgery or radiotherapy and patients with stage T 4a-b colon cancer who had MSI-H (high-frequency microsatellite instability) or dMMR (mismatch repair deficiency) could be enrolled in this study if they met the remaining inclusion and exclusion criteria and obtained informed consent.

Eligible patients received neoadjuvant therapy (see Administration) after enrollment, JS001 + Irinotecan + Bevacizumab, once every 2 weeks for 2 cycles; JS001 + Irinotecan, once every 2 weeks for 1 cycle; after which preoperative examination and preparation were perfected and patients received resection surgery, which should be performed within 4 weeks after neoadjuvant therapy. Specific medication regimens may be adjusted based on newly published clinical data and within the framework of shared decision-making between doctors and patients; if patients do not complete neoadjuvant therapy or disease progression or other reasons require early or delayed surgery, the decision to proceed with the next treatment requires discussion with the clinical multidisciplinary MDT.

Adjuvant therapy with JS001 may be continued 4 weeks (+/- 7 days) after the date of surgery every 3 weeks, and postoperative adjuvant therapy may be adjusted according to the actual clinical situation of the patient. Until disease recurrence, withdrawal of consent or intolerable toxicity, death, or study termination by the sponsor, whichever comes first, adjuvant JS001 therapy will continue for up to 9 cycles (up to 1 2 cycles for overall neoadjuvant and adjuvant JS001 therapy) if the patient remains disease-free, after which patients continue to undergo relapse and survival follow-up.

Pelvic MRI (for rectal cancer) and abdominal + thoracic CT to rule out distant metastasis were performed during the screening period, and enteroscopy was performed to identify the preoperative stage if necessary. Patients underwent an imaging assessment at 6 weeks ± 7 days during the neoadjuvant phase to assess lesion response to neoadjuvant therapy.

Specimen sampling and pathological assessment of resected tumor are conducted according to the standards of diagnosis and treatment. In addition to routine pathological examination and reporting, assessment of tumor regression grade is performed as shown in the table below.

Grading	Description	Presentation
Grade 0	Complete regression	No residual tumor cells
Grade 1	Moderate regression	Single or small foci of residual tumor cells

Grade 2	Minor regression	Residual tumor with extensive fibrotic stroma
Grade 3	No regression	Extensive residual tumor, no or minimal tumor cell necrosis

Complete pathological response (Pathological Complete Response, pCR) is defined as no active cancer cells found in any lesion or nodule after neoadjuvant therapy.

During the study, tumor assessments will be performed by the investigator according to Response Evaluation Criteria in Solid Tumors (RECIST) v1.1 (described in Appendix 4). Patients underwent baseline imaging assessments before the start of neoadjuvant therapy, and the recommended imaging assessments were magnetic resonance imaging (MRI) and abdominal contrast-enhanced computed tomography (CT). Patients will receive imaging assessment every 6 weeks during neoadjuvant phase, and imaging examination 3 days before surgery may be considered if necessary to assess lesion response to neoadjuvant therapy.

Patients may enter the adjuvant phase if the investigator believes that they may benefit from adjuvant therapy. CEA and CA19-9 are routinely performed every 6 weeks and MRI or CT imaging is performed every 12 weeks for 36 months after surgery until disease recurrence, withdrawal of consent, death, or study termination by the sponsor, whichever came first. Enteroscopy is performed if necessary to assess tumor recurrence. And unscheduled imaging or endoscopy may be performed if abnormal elevations of CEA or CA19-9 are found in laboratory tests. CEA and CA19-9 are routinely performed every 12 weeks after 36 months, and MRI or CT imaging and enteroscopy are performed every 24 weeks to assess tumor recurrence.

For patients with radiologically confirmed disease recurrence, survival follow-up will be performed every 3 months to collect information on subsequent anti-tumor treatment and survival until patient death, withdrawal of informed consent, loss to follow-up, or study termination by the sponsor, whichever occurs first.

Patients who discontinue treatment due to non-disease recurrence (e.g., toxicity) or reaching the maximum duration of treatment for JS001 will continue to undergo scheduled tumor assessments until radiologically confirmed disease recurrence, withdrawal of consent, patient death, or study termination by the originator, whichever occurs first. If patients receive other adjuvant therapy or observation follow-up after the completion of adjuvant therapy with JS001, disease recurrence follow-up and survival follow-up should be performed using the same tumor assessment method and frequency as previously described.

Observe any adverse events (AE) occurring in all patients during the clinical study, record the

clinical symptoms, severity, occurrence time, ending time, duration, treatment measures and outcome, and determine their relationship with the study drug. The investigator should follow up all adverse events until resolution to baseline or better, the event is assessed as stable by the investigator, the patient is lost to follow-up, or the patient withdraws consent. Every effort should be made to follow all serious adverse events considered related to the study drug or study procedures until their final outcome can be reported.

No dose modifications of JS001 will be performed in this study. If a patient experiences an adverse event that requires a dose interruption, study treatment may be held 56 days after the last dose. If an adverse event causes JS001 to be suspended for 56 days after the last dose, the patient will discontinue treatment, and patients who discontinue treatment due to serious adverse reactions should be followed up until they return to normal or baseline levels, and patients who cannot tolerate it or patients who experience immune-related adverse reactions can undergo dose interruption or receive glucocorticoid therapy.

Prior to patient enrollment, tumor tissue samples from the patient 's primary tumor will be collected for MSI testing. Resected tumor tissue samples were subjected to pathological evaluation. Peripheral blood samples were also collected continuously after surgery, until radiologically confirmed disease recurrence, withdrawal of consent, death, or study termination by the sponsor, whichever came first. The analysis includes programmed death ligand -1 (PD-L1) expression, TMB detection and ctDNA detection. Patients participating in the biomarker study and providing tumor tissues above need to sign ICF before performing the above operations, and patients can withdraw at any time.

3.2 Duration and end of study

The study started on the date the informed consent form (ICF) was signed by the 1st patient. End of study is the date of last visit (LPLV) for the last patient or statistical analysis, which is the date of collection of the last data point required for the final overall survival (OS) analysis or the date last patient is followed for safety, whichever occurs first.

The duration of this study is expected to be approximately 30 months and the enrollment period is expected to be approximately 6 months with the last patient enrolled.

The neoadjuvant phase, surgery, postoperative adjuvant therapy, and survival follow-up period will last approximately 24 months.

4. Study Population

4.1 Patient Inclusion Criteria

Patients meeting all of the following criteria were eligible to participate

① Special inclusion criteria:

1) Histopathologically confirmed rectal or colon cancer:

   a) Adenocarcinoma

   b) Patients with T3-4 resectable rectal cancer

   c) Patients with T1-2 stage < 12 cm from anal verge who refuse direct surgery or radiotherapy

   d) Patients with T4a-b resectable colon cancer

2) MSI-H, defined as MSI-H confirmed by polymerase chain reaction (PCR) testing, or dMMR, before enrollment, more than 5 tissue FFPE slides are submitted for MSI testing and biomarker study. (Tumor tissue samples must be fresh or archived samples obtained 3 months prior to enrollment; Fresh tissue must be a core needle biopsy or excisional biopsy specimen. EBUS does not yield enough tissue for biomarker review).

3) Have at least one evaluable lesion (including measurable or non-measurable according to RECIST1.1) and did not receive local treatment

② General inclusion criteria:

4) Eastern Cooperative Oncology Group (ECOG) PS score 0-1;

5) Has fully understood the study and voluntarily signed the ICF;

6) Age 18 to 75 years, male or female;

7) Patients who, in the opinion of the investigator, are capable of complying with the protocol;

8) Laboratory values must meet the following criteria within 7 days prior to the start of neoadjuvant therapy:

   a) Neutrophils $\geq 1.5 \times 10^9$/L;

   b) Platelets $\geq 100 \times 10^9$/L;

   c) Hemoglobin ≥ 100 g/L (no packed red blood cell transfusion within 2 weeks);

d) Serum creatinine ≤ 1.5 x upper limit of normal (ULN) and creatinine clearance ≥ 50 mL/min. Creatinine clearance was estimated based on the Cockcroft-Gault formula as follows:

$$= \frac{(140\ \text{Age})\ \text{Body weight (kg)} \times (\text{Female } 0.85)}{0.818\ \text{Serum creatinine } (\mu\text{mol/L})}$$

h) Aspartate aminotransferase (AST), alanine aminotransferase (ALT) ≤ 2.5× ULN;

i) Patients not receiving anticoagulant therapy: INR or aPTT ≤ 1.5 × ULN. Patients receiving prophylactic anticoagulant therapy may be enrolled if INR ≤ 2 ULN and aPTT is within normal values 14 days before the start of study treatment.

j) Serum total bilirubin ≤ 2 × ULN

9) Within 21 days prior to enrollment, women of childbearing potential must have a confirmed negative serum pregnancy test and agree to use effective contraception during study drug use and 60 days following the last dose of study drug. Examples of contraceptive methods with an annual contraceptive failure rate < 1% include bilateral tubal ligation, male sterilization, hormonal contraceptives that inhibit ovulation, hormone-releasing intrauterine devices, and copper ring intrauterine devices. Or double barrier contraception defined as condom containing spermicidal jelly, foam, suppository, or film; or diaphragm containing spermicide and male condom and diaphragm. Reliability of sexual abstinence should be evaluated relative to the duration of the clinical trial and the preferred lifestyle and lifestyle of daily living of the patient. Periodic abstinence (eg, calendar day, ovulation, symptothermal, or post-ovulation methods) and withdrawal are not acceptable methods of contraception. In this protocol
Females of childbearing potential are defined as sexually mature females:

   i. Hysterectomy or bilateral oophorectomy not performed;

   ii. Spontaneous amenorrhea not continuing for 12 consecutive months (amenorrhea following cancer treatment does not exclude childbearing potential) (ie, experienced at any time during the preceding 12 consecutive months Menstruation).

4.2 Patient Exclusion Criteria

Patients meeting any of the following criteria will not be included in this study:

1) Patients who have previously received anti-programmed death receptor -1 (PD-1)

antibody, anti-programmed death ligand - 1 (PD-L1) antibody, anti-programmed death ligand -2 (PD-L2) antibody, or anti-cytotoxic T lymphocyte-associated antigen -4 (CTLA-4) antibody therapy, including patients who have participated in JS001 clinical studies;

2) Presence of clinical manifestations of intestinal obstruction or active bleeding uncontrolled from the primary tumor.

3) Contraindications to bevacizumab or irinotecan.

4) Severe hypersensitivity to other monoclonal antibodies.

5) Have any active, known, or suspected autoimmune disease:

   Patients who are hypothyroidism but on stable doses of thyroid hormone replacement therapy and those with type I diabetes whose blood glucose is controlled may be included in the study.

6) Uncontrolled pleural effusion, pericardial effusion, or moderate ascites;

7) History of idiopathic pulmonary fibrosis, organizing pneumonia (e.g., bronchiolitis obliterans), drug-induced pneumonia, idiopathic pneumonia, interstitial pneumonia, or evidence of active pneumonia on screening chest enhanced CT scan;

8) Major surgery 4 weeks prior to enrollment that has not fully recovered from previous surgery;

9) Active bleeding or coagulation abnormalities [activated partial thrombin time (aPTT) > 43 s, thrombin time (international normalized ratio (INR) > 1.5 × ULN], bleeding tendency or receiving thrombolytic or anticoagulant therapy;

   Prophylactic anticoagulation against an open intravenous infusion system was permitted as long as the drug activity had INR ≤ 2 × ULN and aPTT within normal limits 14 days prior to starting study treatment.

10) Patients who have received previous allogeneic stem cell or parenchymal organ transplantation;

11) Any significant clinical and laboratory abnormalities that, in the opinion of the investigator, affect patients' safety, such as: uncontrolled active infection, uncontrolled diabetes mellitus, hypertension that cannot be controlled within the following ranges by monotherapy (systolic blood pressure < 140 mmHg , diastolic blood pressure < 90 mmHg), II or higher peripheral neuropathy, congestive heart failure, cardiac disease as defined by the New York Heart Association (II or higher), myocardial infarction within

3 months before enrollment, unstable arrhythmia, unstable angina pectoris, chronic kidney disease, thyroid dysfunction, etc., previous or concurrent other malignancies;

12) History of electrolyte disturbances such as uncorrectable serum potassium, calcium, or magnesium
13) Known human immunodeficiency virus (HIV) infection;
14) Active hepatitis B virus (HBV), hepatitis C virus (HCV) infection:
    a) Active viral hepatitis is defined as HBV infection with hepatitis B virus deoxyribonucleic acid (HBV DNA) ≥ 1000 cps/ml or 200 IU/ml or above their upper limit of normal; or HCV infection;
    b) Patients with previous HBV infection or cured HBV infection: Defined as hepatitis B core antibody (HBcAb) positive and hepatitis B surface antigen (HBsAg) negative], can participate in this study. HBV DNA test results of these patients must be < 1000cps/ml or 200 IU/ml or lower than the upper limit of normal, and the doctor judges that the disease is stable and antiviral therapy is not required;
    c) Patients who tested positive for HCV antibodies can be included in the study only if they are tested negative for hepatitis C virus ribonucleic acid (HCV RNA).
    d) Patients cannot be enrolled if they are receiving antiviral therapy at the time of enrollment and are required to maintain stable antiviral therapy throughout the study, and any patients with concerns about compliance are not enrolled.
15) Female patients who are pregnant or lactating, or unwilling to take contraception during the trial;
16) History of other malignancy within 5 years prior to enrollment, except for those with negligible risk of metastases or death [such as malignancy with expected 5-year overall survival (OS) > 90%] and expected to be cured with treatment (e.g., appropriately treated cervical carcinoma in situ, basal or squamous cell skin cancer, localized prostate cancer treated with radical surgery, ductal carcinoma in situ treated with radical surgery);
17) Immunosuppressants, or systemic, or absorbable topical hormonal therapy for immunosuppressive purposes (dose > 10 mg/day prednisone or other equal-potency hormone) and continuing 2 weeks prior to enrollment;
18) Patients with active pulmonary tuberculosis (TB) who are receiving anti-tuberculosis treatment or who have received anti-tuberculosis treatment 1 years prior to screening;
19) Active infection or treatment with oral or intravenous antibiotics 2 weeks prior to the start of neoadjuvant therapy, with the exception of prophylactic use;

20) Any anti-infective vaccine (e.g., influenza vaccine, varicella vaccine, etc.) within 4 weeks prior to the start of neoadjuvant therapy;
21) Receipt of other investigational agents or participation in a clinical study for other treatment purposes within 28 days prior to the start of neoadjuvant therapy;
22) Any other disease, metabolic disorder, physical examination finding, or laboratory abnormality that would contraindicate the use of the trial drug, or compromise the reliability of the study results, or place the patient at high risk for treatment complications, or compromise patient compliance.

4.3 Criteria for discontinuation

Patients have the right to withdraw from the study at any stage of the study. The investigator should ask the patient about the reason for withdrawal from the study and, if possible, ask the patient to return to the site for the last visit and follow up any unresolved AEs as much as possible. If a patient fails to attend the scheduled visit, the investigator should make every effort to reach out to him/her, and make the patient to return to the site as soon as possible to complete the corresponding visit. The investigator also has the right to prematurely terminate the treatment of patients for the following reasons or for other reasons (1 ~ 5 for completing the study treatment; 6 ~ 11 for prematurely terminating the study treatment therapy):

1) JS001 adjuvant therapy has been administered for up to 11 cycles if the patient received adjuvant therapy;
2) Disease recurrence according to RECIST v1.1 criteria;
3) Death;
4) Intolerable toxicity;
5) AE discontinued for more than 56 days and the investigator judged that the patient 's continued treatment with JS001 had a positive risk-benefit ratio;
6) Overall deterioration of health status or discontinuation of the study in the investigator 's best interest;
7) Serious protocol violation;
8) Patient is pregnant;
9) Withdrawal of informed consent by the patient or his/her legal representative;
10) Lost to follow-up (Lost to follow-up is defined as failure of a patient to attend a visit specified in the protocol and failure to reach the patient or his/her family on at least 3 attempts by study personnel 2 or more routes within 3 months of that visit);
11) The sponsor terminated the study.

4.4 Handling of patients who discontinued treatment

The reason for the patient's discontinuation should be documented in the original medical record and the case report form (CRF). Patients who discontinue treatment will be followed up to collect information on subsequent antineoplastic therapy and survival if they do not withdraw the consent of further disclosing information, and this information will be documented in the patient's original medical record and CRF.

If a patient withdraws consent for further disclosure of information, no further assessment of the patient is required and no additional data will be collected.

4.5 Criteria for removal

Before statistical analysis, the principal investigator and statistical unit will judge whether individual cases are excluded. In any of the following cases, the principal investigator should make a comprehensive judgment on whether to exclude the patient from the study based on the degree of completion of the trial, reasons for withdrawal and other factors, and make relevant explanations.

- Individual patient enrollment violates the inclusion/exclusion criteria and should not be enrolled in this trial;
- During the trial, patients fail to follow the trial plan and have poor compliance, such as never using the study drug, or failing to collect samples for immunogenicity and safety evaluation as required by the trial protocol, without any data, etc.;
- During the trial, the investigator considers that the patient has other factors that could not continue to participate in the trial, such as concomitant use of other anti-tumor therapy during the trial, and actually terminates the patient 's continued participation in the trial.

4.6 Replacement of patients

Any enrolled patient who prematurely withdraws from the trial is not allowed to be replaced by an additional enrolled patient. The corresponding number of this patient does not allow reuse of other new patients. Additional enrolled patients will be assigned a new number to enter the study.

4.7 Criteria for termination

1) Major errors in the clinical trial protocol are found in the trial, making it difficult to evaluate the investigational drug;
2) Sponsor requests termination on the premise that patient's rights and safety are fully

protected;

3) The National Medical Products Administration (NMPA) or Ethics Committee (EC) ordered termination of the trial for any reason.

5. STUDY ASSESSMENTS

Specific study flow chart is shown in Appendix I Study Flow Chart. The study was divided into screening/baseline period, neoadjuvant visit, perioperative period, adjuvant visit, end of treatment/early withdrawal visit, and disease-free survival follow-up and survival visit. Following are specific assessments.

5.1 Screening/Baseline Period (-28 to -1 days)

- Signed written ICFs must be provided by all patients prior to the initiation of any study assessments and tests. ICFs will be maintained at the investigational site for all non-enrolled and enrolled patients.
- All screening assessments must be completed and results should be reviewed to confirm that the patient meets all inclusion criteria prior to enrollment. The investigator will use a screening log to record information on all screened patients and, where applicable, confirm patient eligibility or document reasons for screening failure.
- Demographic information, including date of birth, gender, race/ethnicity, height and weight;
- Past medical history and treatment history: collect all past medical history and treatment history prior to signing ICF and relevant for this study;
- Past tumor history and tumor treatment history includes: date of diagnosis of tumor, diagnostic contents and previous systemic or local treatment, including start/end date. Previous significant procedures (e.g., diagnostic or therapeutic invasive procedures such as gastroscopy and needle biopsy) should be recorded in CRF, including start and end dates, name and location of the procedure;
- Tumor tissue collection: All patients agree to provide tumor tissue samples (see Section 8 for details) during the screening visit (it is preferred to provide fresh tumor tissue samples from pre-enrollment biopsy) for tumor biomarker testing and pathological assessment by the pathological laboratory;
- Physical examination, including height (only performed in the screening period), body weight, head, eyes, ears, nose, throat, neck, heart, chest (including lungs), abdomen, limbs, skin, lymph nodes and nervous system;

- Vital signs: body temperature, respiration, blood pressure and heart rate;
- ECOG score: It is recommended that ECOG should be evaluated by the same investigator throughout the study;
- Whole blood cell test;
- Blood biochemistry;
- Urinalysis;
- Coagulation test: APTT, PT, TT, INR;
- Thyroid function test;
- CEA, CA19-9 test;
- Serum pregnancy test;
- Virology test;
- Cardiac ultrasonography: left ventricular ejection fraction (LVEF);
- 12 lead ECG;
- Imaging tumor assessment
- Concomitant medication/Concomitant treatment collection: Within 28 days prior to signing the informed consent form, all medications received by the patient must be recorded in CRF, including the generic name of the medication, daily dose, reason for use of the medication, start date, and end date;
- AE collection: AEs from the signing of informed consent to the first dose should be recorded in CRF.

5.2 Treatment Period Visit

5.2.1 Neoadjuvant therapy phase (predose [1])

- Physical examination, including body weight, head, eyes, ears, nose, throat, neck, heart, chest (including lungs), abdomen, limbs, skin, lymph nodes, nervous system and general condition of the patient;
- Vital signs: body temperature, respiration, blood pressure and heart rate;
- ECOG score: It is recommended that ECOG should be evaluated by the same investigator throughout the study;

- Whole blood cell test 1;
- Blood biochemistry 1;
- Urinalysis 1;
- Coagulation test 1; thyroid function test 1:1 before C1D1 administration and every 2 cycles thereafter;
- 12 lead ECG;
- Cardiac ultrasound: LVEF, C5D1 only;
- Imaging tumor assessment;
- Blood biomarker test: Collected once 60 minutes before dosing;
- JS001 administered; JS001 dosing precautions are detailed in Section 6.2.3; dosing within 24 hours of enrollment
- Collected concomitant medications/concomitant treatments;
- AE collection;
- Patient-Reported Outcomes (PRO): Collected using EORTC QLQ-C30 and CR29 scale questionnaires and self-completed by the patient during visits prior to other non-PRO assessments and study treatment administration.

1 The corresponding examinations in the neoadjuvant phase can be exempted if they are performed within 3 days (72 hours) before administration in the screening phase.

5.2.2 Within 3 days prior to surgery and surgery (tumor imaging assessment can be performed within 7 days)

Preoperative examinations include:

- Physical examination, including body weight, head, eyes, ears, nose, throat, neck, heart, chest (including lungs), abdomen, limbs, skin, lymph nodes, nervous system and general condition of the patient;
- Vital signs: body temperature, respiration, blood pressure and heart rate;
- ECOG score: It is recommended that ECOG should be evaluated by the same investigator throughout the study;
- Whole blood cell test 1;
- Blood biochemistry 1;

- Urinalysis 1;
- Coagulation 1 includes: APTT, PT, TT, INR;
- Thyroid function test 1;
- 12 lead ECG;
- Cardiac ultrasonography: LVEF;
- Tumor assessment (within 7 days prior to surgery): Recommended methods for preoperative imaging assessment include MRI and CT;
- Tumor tissue collection: Fresh tissue samples are collected during operation. Pathology assessment will be performed by BICR and local pathologists. Tumor tissue sections were used for MSI, PD-L1, TMB and other tests;
- Blood biomarker test, refer to Section 7
- Collected concomitant medications/concomitant treatments;
- AE collection;
- Patient-Reported Outcomes (PRO): Collected using EORTC QLQ-C30 and CR29 scale questionnaires and self-completed by the patient during visits prior to other non-PRO assessments and study treatment administration.

1 Laboratory tests (including whole blood cell test, blood biochemistry, urinalysis, coagulation function) must be collected within 3 days before surgery, and surgery can only be started after the laboratory test results meet the criteria for operation at the investigator 's discretion.

5.2.3 Adjuvant therapy phase

Patients continue to receive post-operative JS001 adjuvant therapy and proceed to the adjuvant phase.

- The investigator decided whether patients should receive adjuvant therapy. Adjuvant treatment with JS001 240 mg Q3W should not exceed 11 cycles. Precautions for study drug administration are detailed in Section 6.2.3 3;
- Physical examination, including body weight, head, eyes, ears, nose, throat, neck, heart, chest (including lungs), abdomen, limbs, skin, lymph nodes, nervous system and general condition of the patient; performed every cycle.
- Vital signs: body temperature, respiration, blood pressure, and heart rate; performed

every cycle.

- ECOG score: It is recommended that ECOG be evaluated by the same investigator throughout the study.
- CEA and CA19-9 tests: Within 36 months after adjuvant therapy following surgery, C6D1 began to be examined every 6 weeks until the end of treatment, and at each tumor assessment after the end of adjuvant treatment until recurrence; 36 months later every 12 weeks.
- Whole blood cell test 1.
- Blood biochemistry 1.
- Urinalysis 1.
- Coagulation test: to be performed every 4 cycles starting from adjuvant therapy C1D1 until the end of treatment. Additional tests may be performed as clinically indicated.
- Cardiac ultrasound: LVEF; to be performed every 4 cycles from C1D1 of adjuvant therapy until the end of treatment, and thereafter as clinically indicated.
- 12 lead ECG;
- Thyroid function tests: TSH, FT3, FT4, to be performed every 4 cycles until the end of treatment; additional tests may be performed as clinically indicated;
- Imaging tumor assessment 2: Imaging examinations, to be performed every 12 weeks from the start of adjuvant therapy C1D1 in 36 months after adjuvant therapy. These assessments include pelvic MRI (plain + enhanced) (rectal cancer) and abdominal + thoracic CT (plain + enhanced). MRI may be used if the patient is allergic to CT contrast agent. Enteroscopy will be performed when necessary. These assessments will be performed every 12 weeks after 36 months.
- Tumor tissue collection (if applicable): When the patient's tumor is assessed as recurrent, if there is a tumor lesion suitable for biopsy and the patient agrees to participate in the optional biomarker study, it is recommended that fresh tissue samples be collected and sent to the central laboratory for testing. Patients who choose to participate in the optional biomarker study must additionally sign an independent ICF;
- Blood biomarker test: blood sample will be collected 1 hours before JS001 administration every 12 weeks during adjuvant setting, and at every tumor assessment

after the end of treatment until relapse;

- Collected concomitant medications/concomitant treatments;
- AE collection;
- Patient-Reported Outcomes (PRO): to be collected using EORTC QLQ-C30 and CR29 scale questionnaires, which are self-completed by patients during visits prior to other non-PRO assessments and study drug administration.

1 Laboratory tests (including whole blood cell test, blood biochemistry and urinalysis) during the treatment period must be completed before each dose. Blood samples should not be collected earlier than 3 days before administration, and the administration can only be started after the laboratory test results meet the criteria for continued administration at the investigator 's discretion.

2 In the 12 months after C1D1, CEA and CA19-9 every 6 weeks, MRI or CT imaging every 12 weeks, and enteroscopy when necessary were performed to assess tumor recurrence regardless of JS001 delay. If abnormal elevations of CEA or CA19-9 are detected in laboratory tests suspicious for recurrence, unscheduled imaging or endoscopy may be performed for confirmation until disease recurrence, withdrawal of consent, or death, whichever occurs first.

5.2.4 End of Treatment/Early Withdrawal Visit

Patients have the right to withdraw from the study at any stage of the study. Patients need to be visited within 30 days after the end of treatment or early withdrawal (see Section 4.3), including:

- Physical examination, including body weight, head, eyes, ears, nose, throat, neck, heart, chest (including lungs), abdomen, limbs, skin, lymph nodes, nervous system and general condition of the patient;
- Vital signs: body temperature, respiration, blood pressure and heart rate;
- ECOG score: It is recommended that ECOG should be evaluated by the same investigator throughout the study;
- Whole blood cell test;
- Blood biochemistry;
- Urinalysis;

- Coagulation function test;
- Cardiac ultrasonography;
- 12 lead ECG ;
- Thyroid function test;
- Serum pregnancy test (females of childbearing potential only);
- Imaging tumor assessment;
- Tumor tissue collection (if applicable): When the patient's tumor is assessed as recurrent, if there is a tumor lesion suitable for biopsy and the patient agrees to participate in the optional biomarker study, it is recommended that fresh tissue samples be collected and sent to the central laboratory for testing. Patients who choose to participate in the optional biomarker study must additionally sign an independent ICF; see Section 8 for details;
- Blood biomarker testing;
- Collected concomitant medications/concomitant treatments;
- AE collected.

5.2.5 Disease-free survival follow-up

Patients who discontinue treatment due to non-disease recurrence (eg, toxicity) or due to reaching the JS001 maximum duration of treatment will continue to undergo scheduled tumor assessments until radiologically confirmed disease recurrence, withdrawal of consent, patient death, whichever occurs first.

5.2.6 Survival Follow-up

For patients with radiologically confirmed disease recurrence, survival follow-up will be performed every 3 months to collect information on subsequent anti-tumor treatment and survival until patient death, withdrawal of informed consent, or loss to follow-up, whichever occurs first.

6. STUDY DRUG AND ADMINISTRATION METHOD

6.1 Basic information of study drug

6.1.1 Toripalimab (Abbreviated as JS001)

Toripalimab has been approved for marketing in China, with the strength of 240 mg/6 mL/vial, manufactured by Shanghai Junshi Bio-pharmaceutical Technology Co., Ltd. Toripalimab was supplied to the site through individually packaged kits. There is one bottle per kit. The carton and vial were labeled specifically for the clinical study. Labeling content is consistent with GMP and local regulatory requirements.

6.1.2 Chemotherapy and bevacizumab

Chemotherapy drug, namely irinotecan, and bevacizumab have been approved for marketing in China, and these drugs are packaged and labeled in approved commercial packaging.

6.2 Management of Study Drug

6.2.1 Receipt and Storage

Toripalimab will be provided by Shanghai Junshi Biosciences Co., Ltd. according to the anticipated enrollment plan of the site. Toripalimab will be transported to the site via a third-party logistics company qualified for transport. Upon receipt of study drug, the designated recipient at the site will check the transport, confirm the number and status of drug bottles, complete inventory and drug accountability records, and finally fax the signed delivery note to Junshi Biosciences Co., Ltd. or an authorized third party to confirm receipt of the drug. The drugs are stored at 2 to 8 ° C protected from light.

Toripalimab can only be used in this study and managed only by a person authorized by the investigator. In order to fully control the dispensing and use of the trial medication, storage will be registered at each subject dosing visit.

6.2.2 Disposition of Study Drug

All unused toripalimab will be stored at the site under the specified storage conditions. The investigator will return all unused toripalimab, used residual liquid and empty bottles to Junshi Biosciences or destroy them at the site according to the clinical site procedures at the request of Junshi Biosciences. If the drug is lost or damaged, this should be documented in detail.

6.2.3 Dosing Regimen

Neoadjuvant phase:

Combination Phase of JS001 + Irinotecan + Bevacizumab for 2 Cycles

JS001 Administered dose: 3 mg/kg via intravenous infusion on d1 every 2 weeks,

Bevacizumab Dose administered: 5 mg/kg by intravenous infusion every 2 weeks

Irinotecan administered dose: 180 mg/m 2 via intravenous drip over 2 hours, d1, every 2 weeks.

JS001 + Irinotecan Combination Phase, One Cycle

JS001 Administered dose: 3 mg/kg via intravenous infusion on d1 every 2 weeks

Dose of Irinotecan: 180 mg/m 2 via intravenous drip 2 hours, d1, every 2 weeks

Adjutant phase:

JS001 is given by intravenous infusion at a dose of 240mg every 3 weeks for up to 9 cycles.

6.2.3.1 Preparation, Records and Precautions for JS001

This product is sterile injection, colorless and slightly opalescent. Carefully inspect each bottle of JS001 Injection before use to confirm that there is no damage and that the solution in the bottle does not appear solidification, turbidity or precipitation.

JS001 infusion will be prepared by site staff. Patients must receive JS001 intravenously in the presence of emergency medical facilities and staff trained with emergency monitoring and handling.

Under aseptic conditions, use a disposable syringe to draw the required drug and slowly inject into100 mL normal saline (0.9% sodium chloride solution) infusion bag, gently invert and mix 3 ~ 5 times and avoid shaking with force. After mixing, use sterile, low-pyrogenic, low-protein adsorbed in-line filter (0.2 or 0.22 μm). Final diluted drug concentrations ranged from 0.2 to 20.0 mg/mL. If the prepared diluted solution cannot be used immediately, it can be stored in a refrigerator at 2 to 8 ° C for a maximum of 16 hours or 4 hours at room temperature.

At the end of infusion, patients need to use 100 mL normal saline for flushing. The used drug vials and packages need to be handed over to the drug keeper for recovery. Remains of medication should not be reused.

Accurate documentation of the volume of study drug administered to the patient during the trial was required. Each bottle of JS001 is for single patient use and is not interchangeable. JS001 should not be reused if there is any remaining solution after administration.

JS001 Administration Considerations

1) JS001 Premedication prior to infusion

JS001 should be administered by peripheral or central venous access. Adequate epinephrine,

intravenous diphenhydramine hydrochloride or other anti-allergic agents, and resuscitation equipment should be available prior to infusion for potential severe anaphylaxis. Following infusion, the venous access should remain open for administration if needed. In the absence of complications, the venous access should not be removed until 1 hour observation after the end of the infusion. See Table 1 below for details.

2) Monitoring During JS001 Intravenous Infusion

Patients will receive JS001 administered as an intravenous infusion in a monitored setting equipped with specialized personnel and adequate equipment/medications to manage possible serious reactions in patients at any time. JS001 was administered as an intravenous infusion (IV) in 100 mL 0.9% saline infusion bags over 60 (± 15) minutes followed by an observation period of 60 minutes (required only for the first 2 cycles). Subsequent infusions over 30 (± 10) minutes are permitted if clinically relevant injection-related adverse reactions do not occur during the first 2 cycles. JS001 was administered first on Day 1 of each cycle. See Table 1 below for details.

Some infusion reactions may occur in the subsequent medication stage, and the subsequent medication process should be performed under the supervision of a physician even if no infusion reaction of any grade occurs after the first dose.

3) JS001 Management of Infusion Reactions

If a patient has experienced an infusion reaction, prophylactic anti-allergic medication should be administered before each subsequent study drug administration. In this case, infusion reactions may still occur when antibodies are instilled despite the use of drugs to prevent allergic reactions such as antihistamines and glucocorticoids before administration. Refer to Section 9.2.1 for specific treatment.

Table 1 Monitoring During JS001 Infusion

First JS001 Infusion	Subsequent JS001 infusions
• Prophylactic administration is not permitted;<br>• Record the patient's vital signs (heart rate, respiratory rate, sitting blood pressure, and temperature) 60 minutes after the start of the infusion;<br>• Study drug was instilled over at least 60 minutes;<br>• If clinically indicated, patient vital signs must be closely monitored during infusion [15, 30, 45, and 60 minutes (± 5 minutes)] and vital signs were also monitored within 30 minutes after infusion;<br>• Patients will be informed of possible delayed post-infusion symptoms and asked to reach their study physician if they experience these symptoms.	• If the patient experienced an infusion reaction during previous infusion, appropriate prophylactic administration should be taken for subsequent infusions under the guidance of the investigator;<br>• Record the patient's vital signs at 60 minutes after the start of the infusion;<br>• If the first infusion is tolerated and no infusion-related AE occurs, the study drug infusion may be completed over at least 30 minutes, and if infusion-related occurs in the previous infusion, the study drug infusion may be administered over at least 60 minutes;<br>• If infusion-related AE occurred during previous study drug infusion, vital signs should be also monitored at least 30 minutes after infusion.

6.2.3.2 Preparation methods, records and precautions of combination chemotherapy

Chemotherapy was started 1 hours after the end of JS001 infusion and after close monitoring of various vital signs therapy.

Preparation and precautions of chemotherapeutic drugs were performed according to the package inserts of the drugs used in each site, and the dose was referred to as follows:

Irinotecan: 180 mg/m^2, intravenous drip, d1, once every 2 weeks;

Bevacizumab: 5 mg/kg, intravenous drip, D1, every 2 weeks,

The calculated total dose of bevacizumab was diluted in 100 mL of 0.9% saline solution. If the total dose exceeds 1000 mg, dilute the calculated bevacizumab dose with a sufficient volume of 0.9% saline solution to maintain the final concentration at 2.3-16.5 mg/mL. The minimum volume administered is 100 mL and the infusion volume must be limited as low as possible. Once diluted in 0.9% saline solution, bevacizumab solution must be administered within 8 hours. The first intravenous infusion should last 90 minutes. If the first infusion is well tolerated, the duration of the second infusion can be shortened to 60 minutes. If the 60-minute infusion was also well tolerated, then the subsequent all infusions may be administered over 30 minutes.

6.2.4 Treatment Compliance

Patients must receive JS001 by intravenous infusion at the site. The dose and time of administration of study drug infused during each treatment cycle should be recorded in CRF or the corresponding original record form, and the reasons for delayed administration, dose adjustment or missed administration should also be recorded in CRF. Please refer to Section 11.3.

6.3 Concomitant Medications and Concomitant Therapy

If the patient must take other concomitant drugs during the study due to treatment needs, it must be approved by the investigator and used under the investigator' s guidance.

Symptomatic treatment given due to AE should be documented. Adequate supportive care, including transfusion of whole blood and blood products, antibiotic therapy, liver protection, antiviral therapy, anti-allergic therapy, and anti-diarrheal therapy, may be given to patients if necessary. The date, reason and dosage of treatment should be recorded in detail.

No other radiotherapy, chemotherapy, immunotherapy, hormone therapy (except for anti-allergic drugs) related to anti-tumor, Chinese patent medicines with anti-tumor activity clearly stated in the package insert or other clinical trial drugs are allowed.

Patients are allowed full supportive care during the study.

Patients can use topical, ophthalmic, intra-articular, intranasal, and inhaled corticosteroids (with minimal systemic absorption). Short-term use of corticosteroids for prophylaxis (e.g., contrast dye allergy) or for treatment of non-autoimmune conditions (e.g., delayed hypersensitivity reactions due to contact allergens), or for management of AEs caused by the study drug is permitted.

6.4 Prohibited Medications and Treatments

Patients are not allowed to receive the following medications or treatments during the clinical study:

- Other radiotherapy, chemotherapy, immunotherapy, hormone therapy (except for anti-allergic drugs) related to anti-tumor, and other investigational drugs;
- Traditional Chinese medicine (TCM), which may result in unanticipated drug-drug interactions following the use of TCM, which may lead to or confound the assessment of toxicity;
- Any live attenuated vaccine, which is prohibited within 4 weeks prior to enrollment and during treatment.

Subjects who require any other specific anticancer therapy at the investigator's discretion will

be prematurely discontinued from study drug prior to receiving a new anticancer therapy.

7. Biomarker detection

After patient enrollment, the corresponding specimen collection requirements are as follows:

Before starting neoadjuvant therapy, at least 5 tissue samples from patients are required to be collected for MSI testing, as well as relevant pathology reports of the above samples. 8 ml of peripheral blood samples are also collected for ctDNA testing.

8 ml of peripheral blood samples will be collected within 3 days before surgery and every 4 cycles after surgery for ctDNA testing until the patient develops radiologically confirmed disease recurrence, the patient withdraws informed consent, the patient dies, or the sponsor terminates the study, whichever occurs first. See Appendix 1 for specific collection time.

Optional Biomarker Testing:

PD-L1 and TMB testing will be performed if additional tissue sections are available at screening.

Patients are encouraged to voluntarily participate in optional exploratory studies (biomarker studies) for PD-L1 as well as TMB testing if they could provide tissue from their tumor lesion at surgery or at the time of tumor assessment as recurrence.

2 ml peripheral blood samples will be collected at the same time as samples for TMB testing from patients who participate in the optional biomarker research described above and provide tumor tissue.

Sample Destruction:

Remaining samples obtained from study-related operations will be destroyed 5 years after the end of the study or earlier according to local regulations. If a patient agrees to have tumor samples stored at the Sample Testing and Analysis Unit for further research, these samples will be destroyed 15 years after the final closure of the clinical database.

Processing, transportation, and storage of all specimens listed above will provide detailed operational details in the laboratory manual as well as details in the biomarker analysis plan.

8. Tumor assessment

Tumor assessment during the screening period must be performed within 4 weeks prior to enrollment. During the screening period, patients will undergo pelvic MRI (for rectal cancer), abdominal + thoracic CT, endoscopic ultrasonography (when necessary) to confirm the preoperative staging. If clinically indicated, appropriate methods can be used to examine any other known or suspected sites of disease, such as cranial MRI, bone scan or neck CT scan. For tumor imaging performed for routine diagnosis and treatment before signing ICF, if it is performed within 4 weeks prior to enrollment and at this site, it is not necessary to redo it. The same imaging method should be used for baseline and subsequent assessments and assessed by the same investigator whenever possible.

During the study period, patients are assessed for tumor according to pathological response rate and response evaluation criteria in solid tumors (RECIST) v1.1. Patients undergo imaging assessment at Week 6 in neoadjuvant phase, and imaging examination within 3 days before surgery could be considered if necessary to assess lesion response to neoadjuvant therapy. Recommended imaging assessment methods include pelvic MRI, abdominal + thoracic CT and endoscopic ultrasonography.

Pathologic tumor assessments were pathologic response assessments performed on surgically resected specimens at the end of neoadjuvant therapy, including complete pathologic response rate (pCR) and pathologic response rate assessed by BICR and the local pathology department.

Patients enter the adjuvant therapy phase after surgery and routinely undergo CEA and CA19-9 every 6 weeks during 12 months, MRI or CT imaging every 12 weeks and enteroscopy when necessary to assess tumor recurrence, and CEA and CA19-9 every 12 weeks after 12 months, MRI or CT imaging and enteroscopy (if necessary) will be performed every 24 weeks to assess tumor recurrence.

If abnormal elevations of CEA or CA19-9 are detected in laboratory tests suspicious for recurrence, unscheduled imaging or endoscopy may be performed for confirmation until disease recurrence, withdrawal of consent by the patient, death, or study termination by the sponsor, whichever occurs first.

Patients who discontinue treatment due to non-disease recurrence (e.g., toxicity) or due to reaching the JS001 maximum duration of treatment will continue to undergo tumor assessments as planned above until radiologically confirmed disease recurrence, patient death, withdrawal of consent, or study termination by the originator, whichever occurs first.

Patients with radiologically confirmed disease recurrence will be followed for survival every

3 months using the end of treatment visit as the starting point, and information on subsequent anti-tumor therapy and survival will be collected until patient death, withdrawal of consent, loss to follow-up, or study termination by the initiator, whichever occurs first.

9. Safety/Tolerability Assessment

During the clinical trial, the investigators are responsible for observing any AE in all patients, recording their clinical features, severity, occurrence time, end time, duration, treatment measures and outcome, and determining their relationship with the study drug.

9.1 Monitoring

Safety and tolerability will be assessed in this study by monitoring all AEs and SAEs as defined and graded according to CTCAE v5.0. Patients will be assessed for safety and tolerability (including laboratory results) according to the study flow chart in Appendix 1. Laboratory results must be reviewed at each visit before study drug administration.

General safety assessments are presented in Section 9.5. A list of study assessments and schedule is provided in Appendix I. Patients will be closely monitored for any immune-related adverse events and symptoms of infection during the study.

During the study, all AEs and SAEs are recorded until 60 days after the last dose of study drug or the start of a new anticancer therapy, whichever comes first. Investigators are required to report all AEs and SAEs related to study treatment, regardless of relationship to study drug. In addition, patients will continue to be followed until resolution or return to baseline status if there are unresolved AEs or abnormal laboratory test results that are considered related to study treatment. SAEs are reported in Section 9.8.2.

Patients who continue to experience AEs after the end of the study or at the time of discontinuation of study treatment will continue to be followed until:

- AE resolved or improved to baseline or better;
- The investigator confirms that the event is stable and no further improvement is expected;
- Patient die;
- Patient has lost contact or withdrawn ICF;
- Investigator confirms AE not related to study treatment;
- The patient starts new anticancer therapy.

9.2 JS001 Specific Adverse Event Management

JS001 will be administered during the study in the presence of emergency medical facilities and staff who are trained in emergency monitoring and handling. During the study, all AEs and SAEs are recorded until 60 days after the last dose of study drug or the start of a new anticancer therapy, whichever comes first. Investigators are required to report all AEs and SAEs , whether or not related to study drug.

9.2.1 Infusion reaction

Clinical symptoms of infusion reactions include fever, chills, nausea, pruritus, vasogenic edema, hypotension, headache, tracheospasm, urticaria, rash, vomiting, muscle pain, somnolence, or hypertension. Serious reactions may include acute respiratory distress syndrome (ARDS), myocardial infarction, ventricular fibrillation, and cardiogenic shock. Therefore, patients in this study must be closely observed for relevant clinical symptoms.

Serious reactions require ECG monitoring and rescue medication (including but not limited to epinephrine, corticosteroids, antihistamines, bronchodilators, and oxygen). JS001 Infusion must be stopped immediately in the event of an infusion reaction or suspected event of CTCAE grade 2 or higher. For the first dose of JS001, the infusion should be administered over at least 60 minutes and vital signs should also be monitored for at least 30 minutes after the end of the infusion. Patients will be informed of possible delayed post-infusion symptoms and asked to reach their study physician following the onset of these symptoms.

In case of severe allergic reactions, patients must be treated according to local best medical practice. If a patient experiences a recurrence of an infusion reaction CTCAE grade 2 or higher, the patient must immediately permanently discontinue the drug and withdraw from the study.

Table 2 Treatment Modification Guidelines for Infusion-Related Reactions

CTCAE v5.0 Grade	Adjustment measures
Grade 1 : minimal	Transient minor reactions, interruption of infusion and clinical intervention are not recommended.<br>Slow down dripping speed 50%.<br>Observe closely for any worsening symptoms.<br>Clinical intervention is performed as necessary.
Grade 2 : Moderate	Withhold JS001 and immediately administer systemic therapy (eg, antihistamines, NSAIDs, narcotics, intravenous fluids).<br>Restart and reduce the infusion rate by 50% when infusion-related reactions resolve to grade 0 to 1.<br>During this period, observe closely for any worsening symptoms.<br>Appropriate therapeutic interventions are taken according to medical routine.
Grade 3 : Serious	Stop the infusion immediately and remove the infusion line. The investigator confirms whether the drug is readministered according to the actual situation of the patient.<br>If the drug is restarted, the infusion should be continued for at least 2 hours at the time of subsequent treatment and relevant prophylactic medications (e.g., diphenhydramine and NSAID drugs) should be administered, and the clinical symptoms of relevant infusion-related reactions should be closely observed.<br>Appropriate therapeutic interventions are taken according to medical routine.
Grade 4 : Life-threatening and urgent clinical intervention needed	Patients experiencing grade 4 infusion-related reactions must immediately permanently discontinue the drug and withdraw from the study.<br>Appropriate therapeutic interventions are taken according to local medical practice.

Abbreviations: NSAID = nonsteroidal anti-inflammatory drug.

9.2.2 Serious allergic reaction

Antibody administration may cause allergic reactions. Therefore, immediate provision of appropriate medications and medical equipment to treat acute allergic reactions is crucial and researchers must be trained to recognize and treat allergic reactions. The study site must be equipped with emergency rescue teams and equipment and, if necessary, patients should be transferred to the intensive care unit. In case of severe allergic reactions, the patient must be treated immediately according to the relevant diagnosis and treatment routine. Patients must be given immediate epinephrine and dexamethasone and immediate electrocardiographic monitoring, and consideration may be given to drawing serum IgE samples. Patients must immediately and permanently discontinue study treatment. Patients must inform the investigator immediately if they

experience such symptoms.

Allergic reaction

The National Institute of Allergy and Infectious Diseases (NIAID) and Food Allergy and Allergy Network (FAAN) Anaphylaxis Network guidelines define anaphylaxis as a serious allergic reaction with rapid onset and potential death. These 3 types of anaphylaxes below cover 80% of cases (Category 1) to 95% of cases (all the three categories).

1) Acute onset (minutes to hours) of allergic reaction involving the skin, mucosal tissue, or both (e.g., generalized urticaria, itching or flushing, swollen lips and tongue), with at least one of the following:
   a) Dyspnea [such as dyspnea, stridor-bronchospasm, stridor, decreased peak expiratory flow rate (PEF), hypoxemia];
   b) Decreased blood pressure or associated symptoms of end-organ dysfunction (e.g., hypotensive shock, syncope, incontinence).
2) After allergen exposure (minutes to hours), the patient may experience two or more of the following:
   a) Mucocutaneous involvement (e.g., generalized urticaria, pruritus, swollen lips and tongue);
   b) Dyspnea (e.g., dyspnea, stridor-bronchospasm, stridor, decreased PEF, hypoxemia) ;
   c) Decreased blood pressure or associated symptoms (e.g., hypotensive shock, syncope, incontinence);
   d) Persistent gastrointestinal symptoms (e.g., abdominal pain, vomiting).
3) Decreased blood pressure (minutes to hours) following exposure to a known allergen:
   a) Infants and children: systolic blood pressure decreased (age-specific) or systolic blood pressure decreased by less than 30%;
   b) Adult: Systolic blood pressure less than 90 mm Hg or more than 30% decrease from the person 's baseline.

9.2.3 Immune-related adverse events

JS001 may be associated with the following important irAEs: immune-related liver dysfunction, interstitial lung disease lung disease, pancreatitis, endocrine disorders [hypothyroidism (refer to Appendix 5 for replacement therapy for hypothyroidism), hyperthyroidism, immune-related hyperglycemia, and immune-related adrenal insufficiency].

In addition, irAEs also include the following types of potentially clinically significant events: exfoliative dermatitis, uveitis, arthritis, myocarditis, hemolytic anemia, partial seizures that may

occur in patients with hemispheric inflammation, adrenal insufficiency, myasthenia gravis, optic neuritis, and rhabdomyolysis.

Anticipated Safety Risk Management of JS001 is described in detail in the JS001 Investigator Brochure.

Suspected irAEs require close observation of organ system function and warrant adequate evaluation to identify the cause and rule out other causes. Overall, early attention, early detection, early intervention, suspension or permanent discontinuation of JS001 and/or symptomatic treatment according to the severity of the event, and early administration of adequate glucocorticoids should be performed. When prednisone 1 to 2 mg/kg or a glucocorticoid with the same efficacy is administered, a stepwise dose reduction can be started after the event resolved to grade 0 to 1. Risk management is described in detail in the JS001 Investigator Brochure. At this time, if irAE remains at 0 - 1 grade, JS001 should be re-administered. irAEs of grade 3 or higher (except endocrine disorders) required immediate permanent discontinuation and withdrawal from the study.

9.3 JS001 Dose Modification

No dose reductions of JS001 are required in this study. If a patient has an AE that required dose interruption, the patient could suspend study treatment, and JS001 could be delayed for up to 7 days due to AE, This dose is considered missing if the patient could not take the drug after 7 days delay. Patients will perform the next visit according to the original plan. Consider permanent withdrawal from study treatment if the patient is discontinued for more than 56 days and the investigator determines that continuation of JS001 treatment has a greater risk than benefit,

Treatment with JS001 may be associated with irAEs such as immune-related hepatitis, pneumonia, colitis, pancreatitis, endocrine system disorders (hypothyroidism, hyperthyroidism, adrenocortical insufficiency, hyperglycemia, or diabetes mellitus). Overall, JS001 should be withheld or permanently discontinued and symptomatic treatment, such as corticosteroids, should be administered depending on the severity of the event.

If a patient requires a gradual dose reduction of steroid treatment after an AE, JS001 may be discontinued for a longer period of time until the steroid dose is reduced to a prednisone dose of ≤ 10 mg/day (or equivalent). If the study drug is discontinued for more than 56 days, the investigator could decide whether to restart the study drug based on an overall risk-benefit assessment.

The investigator must document any decision to permanently discontinue the drug.

Further details regarding JS001 dose modifications are provided in the JS001 Investigator Brochure.

9.4 Chemotherapy and bevacizumab safety management and dose modifications

9.4.1 General Principles

General principles for dose modification in combination chemotherapy regimens are as follows:

- If the investigator considers the toxicity to be due to only one drug of the study treatment, the administration of that drug should be delayed or adjusted according to the following guidelines, and if there are no contraindications, the administration of other components should be continued.
- Any patient who requires a dose reduction will continue to receive the reduced dose in subsequent treatment cycles. Any patient who has had 2 dose reductions must discontinue study treatment if a third dose reduction is required due to toxicity. Treatment may be delayed until Day 21 after starting on Day 1 of this treatment cycle to allow adequate time for recovery from study drug-related toxicities.
- For concomitant conditions already present at baseline, dose modifications are made according to corresponding changes in toxicity grade as deemed appropriate by the investigator. For example, if a participant has grade 1 asthenia at baseline that increased to grade 2 during treatment, a grade 1 change occur and is considered a grade 1 toxicity for the purpose of dose modification.
- In case of concurrent severe toxicity of different severity grades, dose modification should be performed according to the highest observed grade.
- No dose adjustment of other chemotherapeutic agents is required if the investigator considers the toxicity to be related to only one chemotherapeutic agent.
- Patients who temporarily discontinue chemotherapy due to adverse reactions may continue treatment with JS001 as originally planned if they meet the requirements for use of JS001.
- Subjects who discontinue chemotherapy due to toxicity should not be completely withdrawn from all study treatment and should continue to complete JS001 of treatment.
- In addition to the chemotherapy dose modifications recommended in this section, dose modifications may also be performed according to local product information and standard clinical practice.
- Specific chemotherapy and bevacizumab toxicity management and dose

modifications are presented in Sections 10.4.2 and 10.4.3.

9.4.2 Irinotecan Safety Management and Dose Modifications

In addition to the chemotherapy dose modifications recommended in this section, toxicity management may also be performed according to local product information and standard clinical practice.

9.4.2.1 For Hematologic Toxicity Dose Modification

If at the start of a treatment course, the subject has an absolute neutrophil count (ANC) ⩾ 1.5 x 10 9/L and platelet count ⩾ 100 x 10 9/L, a new treatment course may be started. Otherwise, treatment needs to be delayed until blood parameters recover. Dose adjustments should be based on the lowest hematologic count in the previous treatment cycle at the start of the subsequent treatment cycle. Treatment may be delayed if relevant tests do not meet requirements to allow adequate recovery time and treatment may be delayed for up to 3 weeks. Detailed guidance on dose modifications according to hematotoxicity following patient recovery is provided in Table 3.

Table 3 Dose Modification Regimen for Irinotecan According to Hematologic Toxicities During Planned Treatment

**Toxic reaction NCI CTC Grade**	**During treatment cycles**	**At start of next course**
No toxic reaction	Maintain dose level	Maintain dose level
Neutropenia		
1	Maintain dose level	Maintain dose level
2	Decrease by 1 dose level	Maintain dose level
3	Withhold until recovery to 2 then decrease 1 dose level	Decrease by 1 dose level
4	Withhold until recovery to 2 then decrease 2 dose levels	Decrease by 2 dose levels
Febrile neutropenia	Withhold until recovered, then decrease 2 dose levels	
Other hematologic toxicities	Agents based on leukopenia and thrombocytopenia during a treatment cycle and at the start of the next course	
	Dose modifications will also be based on NCI toxicity assessment criteria and in line with the dose modifications recommended above for neutropenia	
	The whole scheme is consistent.	

9.4.2.2 Dose Modifications for Non-Hematologic Toxicities: Irinotecan

Dose modifications for irinotecan at the time of non-hematologic toxicity are shown in Table 4

Table 4 Dose Modifications for Irinotecan at the Time of Non-hematologic Toxicities

**Toxic reaction NCI CTC Grade**	**During treatment cycles**	**At start of next course**
No toxic reaction	Maintain dose level	Maintain dose level
Diarrhea		
1	Delay until return to baseline then administer the same dose	Maintain dose level
2	Stop until return to baseline then decrease 1 Dose Level	Maintain dose level
3	Stop until return to baseline then decrease 1 Dose Level	Decrease by 1 dose level
4	Stop until return to baseline then decrease 2 Dose Level	Reduce 2 dose levels
Other non-hematologic toxicities		
1	Maintain dose level	Maintain dose level
2	Withhold until recovery to 1 then decrease 1 dose level	Maintain dose level
3	Withhold until recovery to 2 then decrease 1 dose level	Decrease by 1 dose level
4	Withhold until recovery to 2 then decrease 2 dose levels	Decrease by 2 dose levels

9.4.3 Bevacizumab Safety Management and Dose Modifications

Toxicity management and dose modification regimens are performed according to local product information and standard clinical practice. No dose reduction of bevacizumab is recommended.

Bevacizumab is discontinued in the following cases:

- Gastrointestinal perforation (gastrointestinal perforation, gastrointestinal fistula formation, abdominal abscess), visceral fistula formation
- Wound dehiscence requiring intervention and wound healing complications
- Severe bleeding (e.g., requiring intervention)
- Severe arterial thrombotic events
- Life-threatening (grade 4) venous thromboembolic events, including pulmonary embolism
- Hypertensive crisis or hypertensive encephalopathy
- Posterior reversible encephalopathy syndrome (PRES)
- Nephrotic syndrome

Bevacizumab is to be interrupted if:

- At least 4 weeks prior to elective surgery
- Severe hypertension poorly controlled on medication

- Moderate to severe proteinuria requires further evaluation
- Severe infusion reaction

9.5 Safety Parameters and Definitions

9.5.1 Safety/Tolerability evaluation indicators

- AEs (including SAEs and AESIs) assessed according to CTCAE v5.0 ;
- Vital signs, ECGs, and physical examination changes from baseline;
- Clinical laboratory tests for changes from baseline in [hematology, blood chemistry, coagulation, urinalysis, stool routine (including occult blood), and thyroid function];

9.5.2 Definition of Adverse Events

According to International Conference on Harmonization (ICH) Good Clinical Practice (GCP) 21 , AE refers to any untoward medical occurrence in a clinical investigation in a patient treated with a drug and which does not necessarily have a causal relationship with the treatment. Therefore, AE could be any of the following:

- An AE can be any unfavorable and unintended sign (including an abnormal laboratory finding), symptom, or disease (new or exacerbated) temporally associated with the use of a therapeutic agent;
- Any new condition or worsening of a pre-existing condition (an exacerbation of the manifestation, frequency, or severity of a pre-existing condition), except as described in Section 9.9.11;
- Recurrence of intermittent disease conditions (e.g., headache) not manifested at baseline;
- Any deterioration in a laboratory test result or other clinical test (e.g., ECG) that is associated with clinical symptoms or results in a change in study treatment or concomitant medication, or leads to discontinuation of study drug;
- AEs related to protocol-specified interventions, including AEs that developed prior to assignment to treatment (e.g., invasive screening procedures such as biopsy);
- Signs, symptoms, or clinical sequelae suspected as a result of an overdose of study drug or a concomitant medication (the overdose itself is not to be reported as an AE/SAE).

Events that do not meet the AE definition include:

- Medical or surgical procedures (e.g., endoscopy, appendectomy) should not be

recorded as an AE. However, the condition leading to these procedures should be recorded as an AE, such as appendicitis;

- Conditions without adverse medical events (hospitalization due to social insurance and/or convenience);
- Planned hospitalization required by the protocol (e.g., for use of study drug);
- Expected day-to-day fluctuations in pre-existing diseases or conditions present or detected at the start of the study, but not exacerbated;
- Expected progression, signs or symptoms of disease/disorder under study unless the patient 's status is more severe than expected.

9.5.3 Abnormal Laboratory Findings

Not every laboratory abnormality qualifies as an AE, and the investigator should consider the following guidelines when deciding whether a change in laboratory value is an AE:

- Abnormal laboratory findings leading to a change in study drug administration (eg, dose interruption or permanent treatment discontinuation);
- Requires concomitant/or surgical intervention to mitigate laboratory abnormalities;
- Results in a medical intervention (e.g., potassium supplement for hypokalemia) or a change in concomitant medication;
- Abnormal laboratory findings are associated with clinical symptoms;
- Abnormal laboratory findings were associated with SAEs;
- Abnormal laboratory findings should be AEs when judged clinically significant by the investigator.

9.5.4 Serious Adverse Events

An SAE refers to an untoward medical occurrence that meets one or more of the following criteria:

- Results in death (i.e., AE definitely caused or contributed to death);
- Is life-threatening (i.e., AE puts the patient at immediate risk of death in the opinion of the investigator);

Note: The term life-threatening refers to an event in which the patient is at risk of death at the time of the event; it does not refer to an event which hypothetically might have caused death if it is more severe.

- Requires or prolonged hospitalization;

Note: In general, hospitalization refers to observation (usually at least overnight) in a hospital or emergency ward and/or treatment that is not appropriate in an outpatient department. Complications that developed during hospitalization were AEs. A complication is considered serious if it prolongs hospitalization or meets any other serious criteria. AE should also be considered serious when there is uncertainty as to whether "hospitalization" or "hospitalization" is required. Exceptions not considered AEs are listed in Section 9.5.2 .

- Results in disability/loss of function;

Note: The term disability refers to a substantial disruption of an individual's ability to conduct normal life functions. This definition does not include events of relatively minor clinical significance, such as uncomplicated headache, nausea, vomiting, diarrhea, influenza, and accidental trauma (e.g., sprained ankle), which may impact daily functioning but do not result in significant loss of functioning.

- Congenital anomaly/birth defects in the infant following maternal exposure to the study drug;
- Important medical events (e.g., AEs that may jeopardize the patient or may require medical or surgical intervention to prevent one of the outcomes listed above) that, in the opinion of the investigator, may be medically significant. For example, an event may not immediately be life-threatening or result in death or hospitalization but may threaten the patient or may require medical or surgical intervention to prevent one of the other outcomes listed in the above definitions based on sound medical and scientific judgment. These should also be considered serious, e.g., allergic bronchospasm treated intensively in the emergency room or at home, convulsions that did not result in hospitalization.

Events that clearly follow the expected pattern of progression of the underlying disease **should not** be recorded as AEs, such as jaundice caused by tumor progression compressing the common bile duct, pain caused by tumor bone metastases, intracranial hypertension caused by tumor brain metastases, etc., unless the investigator considers progression to be atypical or accelerated or caused by the study drug.

The term "serious adverse event" is not synonymous with "severity of adverse event". Severity refers to the intensity of the AE (CTCAE v5.0 determination; see Section 9.7); the event itself may be of relatively minor medical significance (e.g., severe headache without any other findings).

Severity and seriousness should be assessed separately for each AE recorded on the CRF .

9.5.5 JS001 Adverse Events of Special Interest (AESI)

Non-serious AESI should be reported to Shanghai Junshi Bio-pharmaceutical Technology Co., Ltd. as required 24 hours after the investigator becomes aware of the event. AESI for JS001, include

- Suspected immune-related myocarditis: increased cardiac enzymes with electrocardiogram changes or clinical symptoms
- Abnormal liver function meeting Hy 's Law criteria:

ALT or AST elevation (> 3 X ULN) combined with total bilirubin elevation (> 2 X ULN) or clinical jaundice, excluding obstructive jaundice or other causes of bilirubin elevation

9.6 Causality assessment of adverse events

Investigators should use their knowledge of the patient's condition, the understanding of the event, and an evaluation of any potential candidate causes to determine whether the AE is related to the study drug. Judgment should take into account the following points:

- Temporal relationship between onset of the event and start of study drug treatment;
- The course of the event, particularly considering the impacts of dose reduction, discontinuation, or reintroduction of study drug on the event (if applicable);
- Known association with study drug or similar therapy;
- Known association of the event with the disease under study;
- Patients with risk factors or concomitant medications that increase the probability of an event;
- Presence of non-treatment-related factors known to be associated with the occurrence of the event.

The relationship between AE and the study drug will be evaluated by the investigator based on his/her clinical judgment in conjunction with the above considerations, and two categories will be applied in the correlation judgment, i.e., related or not related.

9.7 Severity evaluation of adverse events

Severity of AEs will be evaluated according to CTCAE v5.0. If an AE occurs that is not within the range given by this criterion, severity will be assessed using the following table 7:

Table 7 Adverse Event Severity Grading Scale for Events Not Specified in CTCAE v5.0

Level	Severity
1	Mild; asymptomatic or minimally symptomatic; clinical or diagnostic observations only; or no treatment required
2	Moderate; minimal, local, or noninvasive intervention indicated; limiting age-appropriate instrumental related activities of daily living [a]
3	Severe or medically significant but not immediately life-threatening; hospitalization or prolongation of existing hospitalization indicated; disabling; or limiting self-care activities of daily living [b, c]
4	Life-threatening consequences or urgent intervention indicated [d]
5	Adverse event related death [d]

CTCAE Common Terminology Criteria for Adverse Events.

Note: Based on the latest version of this CTCAE v 5.0 , available at

http://ctep.cancer.gov/protocolDevelopment/electronic_applications/ctc.htm

a Instrumental activities of daily living refer to activities such as cooking, purchasing groceries or clothes, calling and financial management;

b Examples of self-care activities of daily living include bathing, dressing, eating, using the toilet, taking medications, that is, activities ambulatory patients are able to engage in;

c If an event is assessed as a "significant medical event," the event must be reported as an SAE as described in Section 9.8.2 ;

d Grade 4 and 5 events must be reported as SAEs (see Section 9.8.2 for reporting guidelines).

9.8 Recording and Reporting of Adverse Events and Serious Adverse Events

All AEs that have emerged since the previous visit must be recorded at each required follow-up visit between studies. The investigator must determine the severity of each AE and the relationship of each AE to study drug, etc. (see Sections 9.5 and 9.6).

9.8.1 Recording and reporting of adverse events

According to GCP in China, the following measures should be taken for all AEs occurring during the course of the clinical study, regardless of the treatment group or relationship to the study drug:

1) The investigator should immediately take appropriate protective measures to ensure the safety of patients, and experts should make diagnosis and state the reasons;

2) In case of study termination, the investigator should also check the patient regularly and write the date of study termination in CRF (Date of discontinuation of investigational product), reason for termination and detailed process;

3) The investigator should follow up all AEs until occurrence of any of the following:
   - AE resolved or improves to baseline or better;
   - The investigator confirms that the event was stable and no further improvement is expected;
   - Patient dies;
   - Patient has lost contact or withdrawn ICF;
   - Investigator confirms AE not related to study treatment;
   - The patient starts new anticancer therapy.

Any AE should be documented in detail on the CRF and reported in the clinical study report.

During the study, if a large number of unexpected adverse reactions related to the study drug occur, they should be immediately reported to EC, NMPA and relevant provincial, autonomous region and municipal drug regulatory authorities.

9.8.2 Reporting of Serious Adverse Events

9.8.2.1 Event started before start of study medication

Following informed consent, but prior to initiation of study medication, only SAEs resulting from protocol-mandated interventions should be reported. The investigator should complete a paper Serious Adverse Event Reporting Form and front page and report to NMPA, Health Administration and EC in writing immediately (i.e., within 24 hours of knowledge of the event).

9.8.2.2 Event occurred after start of study medication

According to GCP in China, for all SAEs that occur during the course of a clinical study (after initiation of the study medication), the following actions should be taken in addition to the treatments described in Section 9.2:

1) The investigator should report to NMPA, health administrative authority and EC in writing with signature and date within 24 hours after learning of SAE, and copy to Shanghai Junshi Bio-pharmaceutical Technology Co., Ltd. (Safety_js001@junshipharma.com; INDIA-SAEMAILBOX@parexel.com; 236365JUNSHI-SAE@parexel.com);
2) The investigator should provide additional information (autopsy report, medical

records of termination phase and other necessary information) on SAEs (including reported deaths or adverse drug reactions) as required by the medical institution and ethics;

3) The investigator should take necessary measures to ensure the safety and rights of the patients.

All AEs and SAEs should be followed up until resolution or improvement to baseline level, death of the patient, loss of contact, or the investigator finally confirms that AEs and SAEs are unrelated to study treatment.

During the study, all SAEs are recorded until 60 days after the last dose of study drug or the start of a new anticancer therapy, whichever comes first. Following this timepoint, investigators are required to report all SAEs related to study treatment, whether or not they develops after study medication.

9.8.3 Reporting and tracking of pregnancies

9.8.3.1 Pregnancy in female patients

Instruct female patients of childbearing potential to inform the investigator of pregnancy immediately during the study or within 60 days of the last dose of study drug if they are pregnant. The investigator should complete the pregnancy report immediately (i.e., within 24 hours of learning of the pregnancy). The investigator should discontinue the study drug and discuss the risks of pregnancy and the potential effects on the fetus with the patient. Patients will be monitored until the end of pregnancy. Any of the following events associated with pregnancy should be reported as SAEs on the AE page of eCRF and the SAE form should be completed:

- Spontaneous abortion;
- Termination of pregnancy for medical reasons;
- Abnormal outcome;
- Fetal congenital anomaly/birth defect or anomaly

9.8.3.2 Pregnancy of female partner of male patient

The investigator must be informed immediately of the pregnancy of male patient's partner during the study or 60 days after the last dose of study drug. The investigator should complete a pregnancy report form immediately (i.e., within 24 hours of learning of the pregnancy), should provide information on the risks of the pregnancy and the potential effects on the fetus, and must follow the pregnancy as closely as possible.

9.8.3.3 Abortion

All spontaneous abortions should be classified as SAEs (as spontaneous abortions are medically significant events) and documented on the AE page of the CRF.

9.8.3.4 Congenital anomaly/birth defect

Any congenital anomaly/birth defect in a child born to a female patient or a female partner of a male patient who has been exposed to study drug should be classified as SAE and documented on the AE page of CRF.

9.9 Procedures for Recording Adverse Events

Investigators recording AEs on the AE page of CRF should use the exact medical terminology/concept and avoid using colloquialisms and abbreviations.Only one AE term can be populated in the event field on the AE page of CRF.

9.9.1 Infusion reaction

AEs that occur during or within 24 hours of study drug administration and are judged to be related to study drug infusion should be recorded as diagnoses (e.g., infusion reactions) on the AE page of the CRF. Whenever possible, ambiguous terminology such as "systemic reactions" should be avoided. If a patient experiences both local and systemic reactions after the same dose of study drug, each reaction should be recorded separately on the AE page of the CRF.

9.9.2 Diagnosis and Signs and Symptoms

In addition to the signs and symptoms, the diagnosis (if known) should also be recorded on the AE page of CRF, e.g. liver failure or hepatitis, not jaundice, asterixis, and transaminases increased. However, if at the time of reporting a single medical diagnosis of a syndrome cannot be made for a set of signs and/or symptoms, each event should be recorded individually on the AE page of CRF. If a diagnosis is subsequently established, all previous events based on the symptoms and signs of that diagnosis should be withdrawn and replaced with a AE report based on a single diagnosis, and the start date of the event is the start date of the first symptom of the final diagnosis.

9.9.3 Adverse Events Secondary to Other Events

In general, AEs secondary to other events (e.g., cascade events or clinical sequelae) should identify their primary cause, with the exception of severe or serious secondary events. If a secondary AE is medically significant and the two occur independently of each other in time, the secondary event should be recorded as a separate event on the AE page of the CRF. For example:

- In healthy adults, if vomiting results in mild dehydration and no additional treatment is required, only vomiting should be reported on the CRF;
- If vomiting results in severe dehydration, both events should be reported

separately on the CRF;

- If severe gastrointestinal bleeding leads to renal failure, these two events should be reported separately on the CRF;
- If dizziness leads to a fall and subsequent fracture, three separate events are required to be reported on the CRF;
- If neutropenia results in an infection, both events should be reported separately on the CRF.

If it is unclear whether there is an association between events, all AEs should be recorded separately on the AE page of the CRF.

9.9.4 Persistent or recurrent adverse events

Persistent AEs refer to AEs that are ongoing and do not resolve between patient assessment timepoints. Such events need only be documented 1 time on the AE page of CRF. The initial severity (severity or grade) of the event is recorded at the time the event is first reported. If an AE worsens, the maximum severity should be recorded on the AE page of the CRF. If an AE meets the criteria for SAE, the investigator must report it immediately (i.e., within 24 hours of learning of the exacerbation; see Section 9.8.2 for reporting instructions). Updated the AE page of the CRF by changing the severity of the event from "non-serious" to "serious", providing the date the event is serious, and completing all data associated with SAE.

Recurrent AEs refer to AEs that resolved at this patient assessment time point compared to the last assessment time point, but subsequently recurred. Each recurrence of AE should be documented separately on the AE page of CRF.

9.9.5 Abnormal Laboratory Values

Not every laboratory abnormality qualifies as an AE. Abnormal laboratory findings must be reported as AEs when they meet any of the following criteria:

- Accompanied by clinical symptoms;
- Results in a change in study treatment (e.g., dose modification, treatment interruption, or treatment discontinuation);
- Requires medical intervention (e.g., potassium supplement for hypokalemia) or changes in the results of concomitant medications;
- Findings considered clinically significant by the investigator.

Note: For oncology trials, certain abnormal values need not be reported as AEs.

It is the responsibility of the investigator to review all laboratory results. Correctly determine

whether an isolated laboratory abnormality should be considered an AE.

If a clinically significant laboratory abnormality is a sign of a disease or syndrome (e.g., ALP and bilirubin > 5 ULN associated with cholestasis), only the diagnosis (i.e., cholestasis) needs to be recorded on the AE page of CRF.

If a clinically significant laboratory abnormality is not a sign of a disease or syndrome, the abnormality itself should be entered on the AE page of the CRF, with an accompanying description indicating whether the test result is above or below the normal range (e.g.,"high potassium" instead of "abnormal potassium"). If a laboratory abnormality is identified by a standard definition and can be characterized by an exact clinical term, the clinical term should be recorded as an AE. For example, an increase in serum potassium to 7.0 mEq/L should be recorded as "hyperkalemia."

If the same clinically significant laboratory abnormality is observed between a visit and another visit, it should only be counted once on the AE page of the CRF (see Section 9.9.4 for details on recording persistent AEs).

9.9.6 Abnormal Vital Signs Values

Not every vital sign abnormality qualifies as an AE and must be reported as AE when the vital sign abnormality meets any of the following criteria:

- Accompanied by clinical symptoms;
- Results in a change in study treatment (e.g., dose modification, treatment interruption, or treatment discontinuation);
- Requires medical intervention or changes in concomitant medication;
- Findings considered clinically significant by the investigator.

All vital sign results should be reviewed by the investigator. Correctly determine whether an isolated vital sign abnormality should be considered an AE.

If a clinically significant vital sign abnormality is a sign of a disease or syndrome (e.g., hypertension), only the diagnosis (i.e., hypertension) needs to be recorded on the AE page of the CRF.

If the same clinically significant abnormal vital sign test result can be observed at each visit, it should not be recorded repeatedly in the AE page of CRF unless there is an etiologic change (see Section 9.9.4 for details of recording persistent AEs).

9.9.7 Abnormal liver function tests

Elevated ALT or AST (> 3×ULN), or elevated total bilirubin (> 2×ULN) or clinical jaundice without cholestasis or other etiology of hyperbilirubinemia should be considered suggestive of

severe liver injury (as defined by Hy 's Law). Therefore, the investigator must report as an SAE when any of the following occurs:

- ALT or AST higher than 3×ULN with total bilirubin higher than 2×ULN (where direct bilirubin ≥ 35%) during treatment;
- Treatment-emergent ALT or AST higher than 3×ULN with clinical jaundice.

The most appropriate diagnosis or laboratory abnormality (if the diagnosis cannot be confirmed) will be recorded as SAE on the AE page of the CRF.

9.9.8 Death

Deaths that occur during the protocol-specified AE reporting period (see Sections 9.5.2 and 9.5.4) should be recorded on the death form attributed to disease progression in the CRF if the investigator determines that the cause of death is cancer progression. All other study deaths (whether or not related to study drug) and deaths due to disease progression should also be reported as SAEs.

Death should belong to the outcome of the event rather than a different event. Events or conditions leading to a fatal outcome should be entered as a single medical concept on the AE page of the CRF. In general, only 1 such events should be reported. The term "**Sudden death**" is used only for sudden and unexpected deaths, which are presumed to be of cardiac origin. If the cause of death is unknown or cannot be ascertained at the time of reporting, it should be recorded as "**Unexplained Death**" on the AE page of the CRF. If the cause of death is subsequently identified (eg, post-autopsy), "unexplained death" should be changed to the identified cause of death.

Deaths occurring during survival follow-up attributed to cancer progression should be recorded on the CRF for deaths attributed to disease progression.

9.9.9 Pre-existing disease conditions

A pre-existing disease state is one that is present at the Screening Visit for this study. This disease state should be documented on the General Medical History and Baseline Physical Conditions CRF.

Pre-existing medical conditions need to be recorded as AEs **only if** they worsen in frequency, severity, or nature during the study. When recording such an event on the AE page of the CRF, care should be taken to convey the concept that pre-existing medical conditions have changed (e.g., "more frequent headaches") using appropriate descriptions.

9.9.10 Disease progression

Events clearly consistent with the expected pattern of progression of the underlying disease

should not be recorded as AEs. These data will only be collected as efficacy analysis data and will only be reported as grade 5 events if death is due to disease progression, with the event name "disease progression". This should be reported in writing to NMPA, health administrative authorities, and EC as required for SAE reporting. In most cases, the expected pattern of progression will be assessed based on radiological assessment criteria such as RECIST v1.1. In rare cases, clinical progression will be determined based on worsening of symptoms. However, objective criteria should be used to demonstrate progression whenever possible. If it is uncertain whether an event is attributed to disease progression, the event should be reported as an AE. Deaths clearly related to disease progression did not need to be copied to the manufacturer.

9.9.11 Hospitalization or prolonged hospitalization

Any AE that results in hospitalization (ie, patient hospitalization) or prolongs hospitalization should be considered a serious AE and should be documented and reported (as defined in Section 9.5.4 SAE) with the following exceptions.

Hospitalization for the following conditions will **not** be considered an AE:

- Hospitalization for temporary medical care;
- Planned hospitalization as designated by the protocol (e.g., for study drug administration, or for efficacy assessments in this study);
- Hospitalization for a pre-existing condition, provided all of the following criteria are met:
  - ➢ Planned hospitalization before the study, or hospitalization for elective surgery during the study due to progression of the disease that could be expected;
  - ➢ The patient does not have AE.
- Hospitalization for progression of related cancer only.

Hospitalization for the following conditions **will not** be considered SAE, but should be reported as AE:

- The patient is hospitalized for outpatient care outside of normal outpatient medical work hours.

10. Data Management

10.1 Data Entry

In this study, patient data will be entered into the designated eCRF and transmitted to the data system confirmed by the sponsor for integration with data from other sources.

Management of clinical data will follow applicable CDISC criteria and data cleaning procedures to ensure completeness of data, such as removing erroneous and inconsistent data. AE and concomitant medication names will be coded using the Medical Dictionary for Regulatory Activities. eCRFs will be retained by the site.

Site staff are responsible for completing eCRFs. For all patients who signed ICF, the investigator or authorized staff should carefully record any item in eCRF in detail and reply to queries. CRA should check all data in eCRF with the data in the patient's original data to ensure correctness.

The investigator should paste the original laboratory test sheet or photocopy on the patient's study medical record. For abnormal laboratory or test data, the investigator must verify and explain whether it is clinically significant. The investigator should fill in the test in strict accordance with the eCRF filling instructions.

10.2 Database lock

Data will be locked when the following conditions are met:

1) All data have been entered into the database;
2) All questions have been resolved;
3) The analysis population has been defined and judged;
4) Locked data files are no longer altered.

11. Statistical Analysis

The statistical analysis plan should be developed after finalization of the protocol and finalized before database lock. The statistical analysis plan will specify and describe in detail all statistical analysis planned according to the main characteristics of the protocol. All statistical analyses are calculated by the Clinical Data Analysis Department using SAS statistical analysis software.

Unless otherwise specified, descriptive statistics will be performed by default for all variables obtained at each observation time point by cohort (colon cancer patients and rectal cancer patients will be analyzed separately) in this study: continuous variables (e.g., age) will be statistically described using the number of observations, mean, median, standard deviation, minimum, and maximum; categorical variables will be statistically described using the frequency and percentage of each category.

11.1 Sample Size

Sample size calculations are selected based on a Bayesian hierarchical model, an Berry et. al. (2013) method extension, through a large-scale simulation study. This method can effectively control type 1 error while improving statistical power compared with other methods. Specific calculation basis and method are detailed in Appendix 6.

11.1.1 Colon cancer

In this study, 28 patients with colon cancer who meet the study inclusion criteria are planned to be enrolled and receive toripalimab (JS001) neoadjuvant therapy with the combination of bevacizumab and chemotherapy will provide 76% power to demonstrate superior rates of pCR as assessed by BICR in colon cancer patients receiving this neoadjuvant therapy compared to historical controls at a one-sided statistical significance level of 0.05. The above sample size calculations are based on the following assumptions:

- 15% BICR pCR rate in colon cancer patients receiving neoadjuvant therapy with toripalimab (JS001) in combination with bevacizumab and chemotherapy
- 3% BICR pCR rate for historical controls

28 patients with colon cancer will be enrolled in two phases: 11 are planned to be enrolled in phase 1, and 17 subjects are planned to be enrolled in the extended phase (phase 2). 11 patients enrolled in phase 1 will be used for real-time efficacy monitoring, as described in Section 11.4.

11.1.2 Rectal cancer

16 patients (including 8 patients in phase 1 and 8 patients in extension phase) with rectal cancer meeting the study entry criteria are planned to be enrolled in this study to receive neoadjuvant therapy with toripalimab (JS001) combined with bevacizumab and chemotherapy, which will provide 84% power to demonstrate that the BICR pCR rate in rectal cancer patients receiving this neoadjuvant therapy is superior to historical controls at a one-sided statistical significance level of 0.05. The above sample size calculations are based on the following assumptions:

- 35% BICR pCR rate in rectal cancer patients receiving neoadjuvant therapy with toripalimab (JS001) combined with bevacizumab and chemotherapy
- 10% BICR pCR rate for historical controls

The above 16 patients with rectal cancer will be enrolled in two stages, 8 planned in the first stage and 8 planned in the expansion stage. The 8 patients enrolled in the first stage will be used for real-time efficacy monitoring, and the specific monitoring methods are described in Section 11.4.

11.2 Data Analysis Set

The sets are analyzed separately for colon cancer patients and rectal cancer patients according to the following data analysis set definitions.

Full Analysis Set (FAS): All patients who sign informed consent and meet the inclusion and exclusion criteria.

Per-Protocol Set (PPS) includes all patient populations who sign informed consent, do not have any major protocol violations, and have valid baseline and primary endpoint measures.

Safety Set (SS): refers to the patient population enrolled in the trial and administered with the study drug at least once.

11.3 Analytical Procedure

Two analyses are planned in this study, corresponding to neoadjuvant therapy and adjuvant therapy, respectively, and the analysis of adjuvant therapy should include data collected throughout the study (including neoadjuvant therapy).

11.3.1 Enrollment, Demographics, and Baseline Disease Characteristics

Subjects in each analysis set and excluded cases are listed, and data on dropouts, demographics, and baseline disease characteristics are summarized based on the FAS set.

11.3.2 Efficacy Analysis

Unless otherwise specified, rectal cancer patients and colon cancer patients will be summarized and analyzed separately in this study. Descriptive statistics: continuous variables (e.g., age) will be statistically described using the number of observations, mean, median, standard deviation, minimum and maximum; categorical variables will be statistically described using the frequency and percentage of each category.

Primary Efficacy Measures Analysis

BICR pCR rate is the primary efficacy endpoint in this study. Corresponding 95% confidence intervals were calculated using the Clopper-Pearson method for pCR rates based on the FAS set. Sensitivity analyses will be performed on the BICR pCR rate based on the PPS set. At the same time, the results of the Bayesian hierarchical model will play an auxiliary supporting role.

Secondary Efficacy Measures Analysis

For the secondary efficacy endpoints, FAS set is the primary analysis set and PPS is the sensitivity analysis set.

For the secondary efficacy endpoints R0 resection rate, pCR rate assessed by the local investigator, pCR rates assessed by both BICR and local investigator, ORR, the same statistical analysis method as the primary efficacy endpoint is used.

95% confidence intervals will be calculated for each TGR grade for the secondary efficacy endpoint of tumor regression grade (TGR) using the same statistical analysis as for the primary efficacy endpoint.

Secondary efficacy endpoints of time to surgery, investigator-assessed EFS, DFS, and OS were estimated using Kaplan-Meier (KM) methods for time-to-event curves, corresponding to median times and 95% confidence intervals, which are estimated by the Brookmeyer-Crowley method using a log-log function transformation to achieve a normal approximation. For DFS12 and DFS24, the rate value at a specific time is first estimated with the KM method, and then the corresponding 95% confidence interval was estimated using Greenwood 's formula.

Descriptive statistics are used to analyze the secondary efficacy endpoint quality of life scores using the EORTC QLQ-C30 and CR29 scales.

11.3.3 Biomarker analysis

Specific analysis methods are described in an independent biomarker analysis plan.

11.3.4 Safety analysis

Safety analyses are based on the SS set. Frequency tables are used to count the occurrence of AEs, as well as the number and incidence of abnormal laboratory values. Each AE is listed, along with the severity of laboratory abnormalities and causal relationship to study drug. Statistical

description is used to summarize the laboratory test indicators and vital sign indicators at each follow-up visit in each group. Continuous variables are described by the number of cases, mean, standard deviation, minimum and maximum values, and categorical variables are described by the frequency and percentage. Body weight, body temperature, respiration, blood pressure, heart rate, and laboratory parameters are statistically described, and their differences before and after the test are compared.

- Calculate AE incidence;
- Frequency and frequency of AE will be listed by subsystem, and percentages will be calculated;
- Detailed listing of each AE case;
- Number and rate of laboratory parameters, ECG and physical examination findings that "change from normal to abnormal" or "increase of abnormality" after the test;
- Laboratory parameters, ECG, physical examination abnormalities, and clinical interpretations are listed;

11.4 Efficacy real-time monitoring

In order to reduce the risk of exposing patients to ineffective treatment regimens, the efficacy of toripalimab (JS001) combined bevacizumab and chemotherapy as neoadjuvant therapy for patients with colon cancer and rectal cancer will be monitored in real time as described below.

According to the current observed efficacy data (mainly in the case of pCR), if a Bayesian model is applied, early termination of a cohort may be considered if it is predicted that there is little chance (e.g., less than 30%) that the pCR rate in a group after the end of the first stage is higher than the corresponding historical control rate. For the final invalid termination judgment, the investigator may make a comprehensive judgment decision in combination with other data. In this trial, when the number of pCR evaluable patients is greater than or equal to 5, real-time monitoring of efficacy is performed for each cohort. Invalid termination may be judged by referring to the bounds listed in the following table:

Table 8 Critical value of invalid termination for monitoring of efficacy in colon cancer and rectal cancer

Colon Cancer Cohort (Historical Control Rate 3%)			
Number of patients enrolled	0-5	6-10	
Early Termination, if complete response	/	≤ 0	
Rectal Cancer Cohort (Historical Control Rate 10%)			
Number of patients enrolled	1-5	6	7
Early Termination if pCR	/	≤ 0	≤ 1

11.5 Extended Decision

When 8 patients with rectal cancer are evaluable for efficacy in the first stage in the rectal cancer cohort or 11 patients with colon cancer are evaluable for efficacy in the colon cancer cohort, the probability of success (POS) in the continued enrollment trial is predicted according to the Bayesian hierarchical model to assist in comprehensively judging whether the two cohorts of colorectal cancer and colon cancer could continue enrollment.

Based on the currently observed pCR data in both cohorts and the Bayesian hierarchical model, if the POS of that the pCR rate is higher in a cohort than the corresponding historical control is less than 20%, consideration should be given to terminating the cohort for continued expansion. Meanwhile, if the predicted calculated POS is greater than 80% (Wang et. al, 2013), consideration may be given to continued expansion of the cohort. Final extension decisions will be based on calculations and will be made in full consultation with the investigator and based on maximizing patient benefit.

For the colon cancer cohort, POS is calculated as follows:

$$\mathrm{POS} = \mathrm{E}_{y_f|y_{obs}}(\text{Successful trial}) = \sum_{y_{f=0}}^{N_2=17} \mathrm{I}(\text{test success})\mathrm{P}(y_f|y_{obs})$$

$y_f|y_{obs} \sim \text{beta} - \text{binomial}(N - N_1, 1 + y_{obs}, 1 + N - y_{obs})$Equation (1)

y_{obs} refers to the number of pCR cases observed in the first stage of colon cancer cohort, y_f refers to the number of pCR cases observed in the predicted expansion stage; N_1 and N_2 refers to the number of patients enrolled in the first stage and the second stage, respectively; $\mathrm{I}(\text{test success})$ refers to the schematic function, if the conditions are met, the value is 1, otherwise the value is 0 , and the success of the trial here is significant for the final test statistics. 0-11 of the first 11 patients will have pCR, and all possibilities are listed in the table below:

y_{obs}	0	1	2	3	≥ 4
POS	0.10	0.44	0.82	0.98	> 0.99

In addition, the$y_f|y_{obs}$ posterior predictive distribution under the Bayesian hierarchical model can be used instead of Equation (1) for POS calculation.

For the rectal cancer cohort, $N_2 = 8$. List all possibilities as follows:

y_{obs}	0	1	2	3	4	$\geqslant 5$
POS	0.01	0.11	0.44	0.82	0.98	> 0.99

12. Study Management

This study will strictly comply with the laws and regulations related to clinical studies in China, including GCP and Declaration of Helsinki (2013) and other regulations, as well as the conduct of this study protocol. Specific study conduct procedures will be subject to the standard operating procedures (SOPs) of each participating party.

12.1 ETHICAL CONSIDERATIONS

The study protocol, ICF, CRF and other materials must be submitted to EC for review and approval before the start of the study. EC will review and approve these materials in strict accordance with the requirements of relevant laws and regulations, and issue the approval letter after approval. The study will not start until the approval from EC is received.

During the study, any modifications to the protocol must also be reviewed and approved by EC before implementation.

12.2 Informed Consent

The investigator or designated representative will be responsible for explaining study background, pharmacological features of study drug, study protocol and benefits and risks of participation in the study to each patient, and obtain written informed consent signed by the patient or legal representative and study physician before the patient enters the study (before screening examination).

The final ICF text should contain the following contents: study objectives, study procedures, patient 's obligations, foreseeable benefits of participation to patients, and foreseeable risks and inconveniences; treatments and appropriate insurance compensation available to patients in the event of study-related injuries; access to study data and confidentiality of patient' s information. The ICF should be approved in writing by the relevant regulatory authority in accordance with regulations and written in a language readable to the patient.

The ICF must be signed and dated by the patient or his/her legal representative, the investigator performing the informed consent process, or his/her representative. Original ICFs should be kept by investigator and patient respectively. If significant new data involving the investigational drug are identified, the informed consent must be obtained again after the ICF has been modified in writing and submitted to the relevant regulatory authority for approval.

12.3 Subject confidentiality

It is the responsibility of the investigator to maintain anonymity of the subjects. Subjects will be identified only by capital letters, numbers, and/or codes and not by their names on the CRF or

other documents.

The investigator must keep the documents indicating the subject 's identity strictly confidential, and must keep the documents recording the subject' s code, name and home address.

12.4 Compensation for patient health damage

If a patient suffers any injury causally related to participation in this study, the sponsor will bear the cost of treatment and provide corresponding economic compensation according to relevant national laws and regulations. The sponsor will not compensate for damages caused by medical malpractice or failure to comply with the study protocol.

12.5 Recording and Retention of Study Data

To ensure the evaluation and supervision of NMPA, the investigator should agree to retain all study data, including the confirmed records of all patients (which can effectively check all records, such as CRF and hospital original records), all original signed patient ICFs, all CRFs, and detailed records of drug dispensing. Retention period will be 15 years after the end of the study or until destruction is notified by the sponsor.

All materials of this clinical study are proprietary to the sponsor, and the investigator should not provide them to third parties in any form without written consent, except as required by NMPA.

12.6 Return or destruction of study/treatment supplies

All investigational/therapeutic products will be destroyed at the study site after the end of the study.

Destruction of study drugs/therapeutic products at the study site must be performed by appropriate personnel. Formal signed Certificate of Destruction should be completed if destruction of study/therapeutic products is performed by designated personnel.

12.7 Quality Control and Quality Assurance

To ensure the quality of the study, relevant study personnel participating in the study should be trained in GCP.

Each site must manage study drug according to SOP, including receipt, storage, dispensing, and recovery.

12.8 Monitoring and Audit Monitoring:

The CRA authorized by the sponsor has the right to consult CRF, ICF and all the original data at any time.

The Clinical Monitor will be responsible for the development of plans and procedures to be

followed for monitoring this study. An on-site visit will be performed before the start of the study. Regular visits are required during the conduct of the study. Contact may be made by telephone, fax or mail as a supplement to an on-site visit, as needed.

Prior to the start of the study, the investigator will be notified of the anticipated frequency of monitoring visits. In addition, during the course of the study, the investigator will be notified in advance prior to each monitoring visit. The purpose of the visits is to ensure that the clinical study is conducted in strict compliance with the study protocol, and the case report forms are complete, accurate, and verifiable from the source documents.

The CRA will verify that all CRFs are completed and correct, and are consistent with the original data, and all errors or omissions are corrected or indicated, and signed and dated by the investigator. At each visit, in order to review and confirm the case report forms, drug supply and inventory records, drug dispensing and recovery records, and any additional scheduled records, close cooperation is required between the investigator and the clinical monitor.

The initiator or authorized person of the initiator may audit the quality of the study, and the auditor has the right to inspect all study-related medical records, the investigator folder, and correspondence, as well as the ICF.

Audited:

Auditors may audit this study at appropriate times according to relevant SOPs to ensure that the study is conducted in accordance with national regulations, SOPs and the protocol. Audit scope includes office files and site files. After the audit, the auditor will provide a written report to describe the identified problems and make suggestions. The relevant personnel (the investigator and the monitor) should take corresponding corrective measures and make a written record.

12.9 Modification of Study Protocol

Any significant changes to this protocol will require written amendment agreed upon by the investigator and submitted to EC for approval and NMPA filing prior to implementation.

12.10 Study termination

The sponsor reserves the right to stop the study at any time for medical reasons or for any other reason. If the study is prematurely terminated or interrupted, the sponsor shall promptly inform the investigator that the study has been terminated or interrupted and explain the reasons for termination or suspension. According to the requirements of relevant regulations, the sponsor should also immediately inform EC that the study has been terminated or interrupted and explain the reasons.

12.11 Study Summary Report

At the end of the study, the Contract Research Organization (CRO) will assist the sponsor in objectively summarizing the study results and statistically analyzing the study data with appropriate statistical methods, objectively evaluating the safety of the drug according to the results, and making a written summary report of this clinical study after reviewed and approved by the sponsor.

12.12 Confidentiality and Publication of Study Results

Information and data associated with this study are confidential and may not be cited or published without the consent of the sponsor.

The sponsor has the right to publish information or data related to this study or submit them to NMPA. If the investigator 's name needs to appear in the publication or advertisement, the investigator' s consent should be obtained.

13. References

1. Chinese Guidelines for Diagnosis and Treatment of Colorectal Cancer (2017 Edition), Chinese Journal of Surgery 2018 4 , Vol. 56 , No. 4
2. Rolf S, Heinz B, Werner H, et al. Preoperative versus Postoperative Chemoradiotherapy for Rectal Cancer. N Engl J Med 2004; 351:1731-40;
3. De Caluwé L1, Van Nieuwenhove Y, Ceelen WP. Preoperative chemoradiation versus radiation alone for stage II and III resectable rectal cancer. Cochrane Database Syst Rev. 2013 Feb 28; (2)
4. O'Connell MJ, Colangelo HL, Beart BW, et al. Capecitabine and Oxaliplatin in the Preoperative Multimodality Treatment of Rectal Cancer: Surgical Points From National Surgical Adjuvant Breast and Bowel Project End Trial R-04. JCO 2014; 32:1927- 1934 ;
5. Deng YH, Pan C, Lan P, et al. Modi ed FOLFOX6 With or Without Radiation Versus Fluorouracil and Leucovorin With Radiation in Neoadjuvant Treatment of Locally Advanced Rectal Cancer: Initial Results of the Chinese FOWARC Multicenter, Open- Label, Randomized Three-Arm Phase III Trial. JCO 2016; 34:3300-3307 ;
6. Landry JC, Feng Y, Prabhu RS, et al. Phase II Trial of Preoperative Radiation With Concurrent Capecitabine, Oxaliplatin, and Bevacizumab Followed by Surgery and 5 Fluorouracil, Leucovorin, Oxaliplatin (FOLFOX), and Bevacizumab in PatientsWithally Locally Advanced Clinical Cancer: 5 Year Postoperative ECOG- ACRINCancerOncologist Groutal 3204. Clinical Researcher 2015; 20:615-616 ;
7. Aschele C1, Cionini L, Lonardi S, et al. Primary tumor response to preoperative Chemoradiation with or without oxaliplatin in locally advanced rectal cancer: pathologic results of the STAR-01 randomized phase III trial. JCO 2011; 29:2773-80
8. Azria D1, Doyen J2, 3, Jarlier M4, et al. Late toxicities and clinical outcome at 5 years of the ACCORD 12/0405-PRODIGE 02 trial comparing neoadjuvant chemoradiotherapy regimens for intermediate-risk rectal cancer. Ann Oncol. 2017; 28:2436-42
9. Ganesh K1, Stadler ZK2, Cercek A2 et al. Immunotherapy in colorectal cancer: rationale, challenges and potential. Nat Rev Gastroenterol Hepatol. 2019 Mar 18
10. Le, D. T. et al. PD-1 blockade in tumors with mismatch- repair deficiency. N. Engl. J. Med. 372, 2509 – 2520 (2015).
11. Overman, M. J. et al. Nivolumab in patients with metastatic DNA mismatch repair-deficient or instability- high colorectal cancer (CheckMate 142): an open- label, multicentre, phase 2 study. Lancet Oncol. 18, 1182 – 1191 (2017).
12. Overman, M. J. et al. Durable clinical benefit with nivolumab plus ipilimumab in DNA mismatch repair-deficient/instability- high metastatic colorectal cancer. J. Clin. Oncol. 36, 773 – 779 (2018).
13. Grootscholten, C. et al. Neoadjuvant ipilimumab plus nivolumab in early stage colon cancer. Ann. Oncol. 29, LBA37PR (2018).
14. Gandhi L., Rodríguez-Abreu D., Gadgeel S., Esteban E., Felip E., De Angelis F., Domine M., Clingan P., Hochmair M.J., Powell S.F., KEYNOTE-189 Investigators Pembrolizumab plus

chemotherapy in metastatic non-small-cell lung cancer. N. Engl. J. Med. 2018; 378:2078 – 2092.

15. Apetoh L, Ghiringhelli F, Tesniere A, et al. Toll-like receptor 4-dependent contribution of the immune system to anticancer chemotherapy and radiotherapy. Nat Med 2007; 13:1050-59.
16. Pishvaian MJ, Lee MS, Ryoo B-Y, et al. LBA26Updated safety and clinical activity results from a phase Ib study of atezolizumab + bevacizumab in hepatocellular carcinoma (HCC). Ann Oncol 2018; 29 (suppl8).
17. P.M. Forde, J.E. Chaft, K.N. Smith , et al. Neoadjuvant PD-1 Blockade in Resectable
18. Lung Cancer [J]. NEJM 2018, 378:1976-86.
19. Powles, Thomas; Rodriguez-Vida, Alejo; Duran, Ignacio, et al. A phase II study investigating the safety and efficacy of neoadjuvant atezolizumab in muscle invasive bladder cancer (ABACUS), ASCO abs 2018 4506.
20. Wise-Draper, Trisha Michel; Old, Matthew O, et al. Phase II multi-site investigation of neoadjuvant pembrolizumab and concurrent adjuvant radiation and pembrolizumab with or without cisplatin in resected head and neck squamous cell carcinoma. ASCO 2018 abs 6017
21. Sampson H A, Muñoz-Furlong A, Campbell R L, et al. Second symposium on the definition and management of anaphylaxis: summary report — Second National Institute of Allergy and Infectious Disease/Food Allergy and Anaphylaxis Network symposium [J]. Journal of Allergy and Clinical Immunology, 2006, 117 (2): 391-397.
22. International Council for Harmonisation of Technical Requirements for Pharmaceuticals for Human Use. ICH Harmonized. Integrated Addendum to ICH E6 (R2): Guideline for Good Clinical Practice E6 (R2). 9 November, 2016.
23. Good Clinical Practice , China Food and Drug Administration , 2003 08 06 .
24. World Medical Association. World Medical Association Declaration of Helsinki. Ethical principles for medical research involving human subjects [J]. Bulletin of the World Health Organization, 2001, 79 (4): 373.

14. APPENDICES

Appendix 1 Study Flow Chart

	Screening Period	Neoadjuvant therapy					Surgical procedure	Adjuvant [2]		End of treatment /Early withdrawal Visits	Disease-free survival Visit	Survival Follow-up
	Time	C1D1 (± 3 days)	C2D1 (± 3 days)	C3D1 (± 3 days)	C4D1 (± 3 days)	C5D1 (± 3 days)	Corresponding examinations 3 days before surgery	C6-7D1 (± 3 days)	C8D1 to PD or intolerable toxicity (± 3 days)	Last dose Within 30 days	± 7 days	± 7 days
Informed Consent [1]	X											
Demographic information [3]	X											
Medical history (including tumor history and treatment history) [4]	X											
Tumor tissue collection [5]	X						X					
Patient reported outcomes		X	X	X	X	X	X	X	X			
Physical examination [6]	X	X	X	X	X	X	X	X	X	X		
Body weight	X	X	X	X	X	X		X	X			
Height	X											
Vital signs	X	X	X	X	X	X	X	X	X	X		
ECOG score	X	X	X	X	X	X	X	X	X	X	X	
Blood routine [7]	X	X	X	X	X	X	X	X	X	X		
Urine routine [8]	X	X	X	X	X	X	X	X	X	X		
Blood chemistry [9]	X	X	X	X	X	X	X	X	X	X		
Coagulation [10]	X	X	X	X	X	X	X	X	X	X		
CEA, CA19-9 [11]	X								X		X	
Thyroid function [12]	X	X		X		X	X	X	X	X		
Virology Screen [13]	X											
Serum pregnancy test for females of childbearing potential only) [14]	X									X		

12 Lead ECG [15]	X	X	X	X	X	X	X	X	X	X		

	Screening Period	Neoadjuvant therapy					Surgical procedure	Adjuvant [2]		End of treatment /Early withdrawal visit	Disease-free survival Visit	Survival Follow-up
	Time	C1D1 (± 3 days)	C2D1 (± 3 days)	C3D1 (± 3 days)	C4D1 (± 3 days)	C5D1 (± 3 days)	Corresponding examinations 3 days before surgery	C6-7D1 (± 3 days)	C8D1 to PD or intolerable toxicity (± 3 days)	Last dose Within 30 days	± 7 days	± 7 days
Cardiac ultrasound [16]	X					X	X	X	X	X		
Peripheral blood biomarker testing [17]		X					X	X	X	X		
Radiographic tumor assessment [18]	X				X		X	X	X	X	X	
Tumor pathology assessment [19]							X					
JS001 [20]		X	X	X	X	X		X	X			
Bevacizumab		X	X	X								
Oxaliplatin		X	X	X	X			X				
Capecitabine		X	X	X	X			X				
Concomitant medications/Concomitant therapies [21]	X	X	X	X	X	X		X	X	X		
Adverse events [22]	X	X	X	X	X	X	X	X	X	X		
Survival follow-up and subsequent anti-tumor treatment [23]												X

Remarks:

1. Written informed consent (ICF) must be obtained from the patient prior to the initiation of all study-specific procedures in this study;
2. The adjuvant therapy phase consists of 3 weeks cycles of study drug administration on 1 (D1) of each cycle until disease recurrence, intolerability, or for a maximum of 9 cycles.
3. Demographic information: including date of birth, gender, race/ethnicity;
4. Previous tumor history and tumor treatment history include date of tumor diagnosis, diagnostic contents, previous systemic or local treatment, including start/end date; previous significant operation (such as enteroscopy, biopsy and other diagnostic or therapeutic invasive operation) shall be recorded in case report form, including start and end date, name and site of operation;
5. All patients must provide MSI testing of tumor tissue samples prior to enrollment (it is preferred to provide fresh tumor tissue samples from the pre-enrollment biopsy). Tumor tissue is obtained intraoperatively for pathologic assessment. Patients are encouraged to provide tumor tissue samples at the time of tumor assessment for recurrence, and if there are tumor lesions amenable for biopsy and the patient agree to participate in the optional biomarker study, tumor tissue samples are sent to the central laboratory for PD-L1 and TMB testing. Note: 2 ml peripheral blood samples for TMB should be collected

simultaneously from tumor tissue samples for exploratory study;

6. Physical examination: including height (at screening only), body weight, head, eyes, ears, nose, throat, neck, heart, chest (including lungs), abdomen, limbs, skin, lymph nodes, nervous system and general condition of the patient;
7. Whole blood test includes: red blood cell count, hemoglobin, hematocrit, white blood cell counts and differential [neutrophil, lymphocyte, eosinophil, monocyte, basophil and other cell] and platelet count. If the test is completed within 72h before the first dose during the screening period, no re-test is required before the first dose;
8. Urinalysis includes: urine glucose, protein, cast, ketone body, blood cells; 24 -hour urine protein quantitation is required if urine protein test + + and above or if the physician judges the results to be abnormal and clinically significant. If these tests are completed within 72h before the first dose during the screening period, no re-test is required before the first dose;
9. Blood biochemistry includes: total protein, albumin, globulin, blood glucose, total cholesterol, low density lipoprotein, high density lipoprotein, triglyceride, urea nitrogen or urea nitrogen, creatinine, alkaline phosphatase (ALP), lactate dehydrogenase, total bilirubin, direct bilirubin, indirect bilirubin, AST, ALT, calcium, phosphorus, magnesium, potassium, sodium, chloride, serum amylase, uric acid, serum creatine kinase (CK), and serum creatine kinase isoenzyme (CK-MB). If the test is completed within 72 hours before the first dose during the screening period, it is not necessary to perform the test again before the first dose;
10. Coagulation function includes: thrombin time (APTT), prothrombin time (PT), thrombin time (TT), and international normalized ratio (INR). If the test is completed within 72 hours before the first dose during the screening period, it is not necessary to repeat before the first dose;
11. CEA, CA19-9 tests: CEA and CA19-9 are routinely performed every 6 weeks within 36 months after surgery, and unscheduled imaging or endoscopy could be performed to confirm suspected recurrence if abnormal elevations of CEA or CA19-9 are detected in laboratory tests until disease recurrence, withdrawal of informed consent by the patient, death of the patient, or termination of the study by the sponsor, whichever comes first. CEA and CA19-9 are routinely performed every 12 weeks after 36 months.
12. Thyroid function tests included: thyroid stimulating hormone (TSH), serum free triiodothyronine (FT3), serum free thyroxine (FT4). Screening period, pre-dose every 2 cycles of neoadjuvant therapy, 3 days before surgery and 4 weeks after surgery, pre-dose every 4 cycles of adjuvant therapy, and end-of-treatment visit (adjuvant therapy for the first time). If clinically significant changes in thyroid function occur, consultation with endocrinology department and relevant pituitary function tests are recommended;
13. Virologic testing included hepatitis B [including hepatitis B surface antibody (HBsAb), hepatitis B surface antigen (HBsAg), and hepatitis B core antibody (HBcAb); hepatitis B virus deoxyribonucleic acid (HBV DNA) copy number] and hepatitis C virus (HCV) antibody [HCV antibody positive were required for HBsAg or HBcAb positivity, Hepatitis C virus ribonucleic acid (HCV RNA) copy number] and human immunodeficiency virus (HIV) antibodies must be detected;
14. Serum pregnancy test: Blood pregnancy test is required for female recipients of childbearing potential during screening period and end of treatment visit;
15. 12 Lead ECG: QT, QTc, and P-R intervals should be noted. It can be added at any time if there is chest pain, palpitations, or other cardiac symptoms. If the test is completed within 72h before the first dose during the screening period, no re-test is required before the first dose;
16. Cardiac ultrasound: including left ventricular ejection fraction (LVEF). Cardiac ultrasound should be conducted in screening period, prior to neoadjuvant C3D1 administration, 3 days prior to surgery, and every 4 cycles (12 weeks) in the first 12 months since C1D1 in adjuvant phase, and thereafter as clinically indicated if there is chest pain, palpitations, or other cardiac symptoms.
17. Peripheral blood biomarker testing: collected once before neoadjuvant C1D1 administration, once within 3 days before surgery, once within 3 days before C1D1 in adjuvant phase, every 4 cycles until the patient completed the end-of-treatment visit and every tumor assessment after the end of treatment until relapse;
18. Imaging tumor assessment: tumor assessment must be performed within 4 weeks prior to enrollment during the screening period;
   Patients with rectal cancer: Pelvic MRI (plain scan + enhanced scan) and abdominal CT (plain scan + enhanced scan) are performed for evaluation. Chest CT (plain scan + enhanced scan) should be performed during the screening period to rule out distant metastasis until the patient progressed, and chest CT (plain scan + enhanced scan) should be performed again when progression is confirmed. If the patients is allergic to CT contrast agent, MRI can be used.

Enteroscopy may be performed if necessary;

Patients with colon cancer: Pelvic + abdominal CT (plain + enhanced scan) is performed for evaluation. Chest CT (plain scan + enhanced scan) should be performed during the screening period to rule out distant metastasis until the patient progressed, and chest CT (plain scan + enhanced scan) should be performed again when progression is confirmed. If the patients is allergic to CT contrast agent, MRI can be used.

If clinically indicated, appropriate methods can be used to examine any other known or suspected sites of disease, such as cranial MRI, bone scan, or neck CT scan. Tumor imaging performed for routine diagnosis and treatment before ICF is signed by the patient does not need to be redone if performed within 4 weeks before enrollment and at this site. Subsequent assessments should be performed using the same imaging modality and by the same investigator whenever possible. During the study, patients will have tumor assessments performed by the investigator according to Response Evaluation Criteria in Solid Tumors (RECIST) v1.1 during the study. Patients underwent an imaging assessment at 6 weeks (± 7 days) in the neoadjuvant phase. In the adjuvant phase after surgery, MRI or CT imaging is performed every12 weeks (± 7 days) for 36 months, and enteroscopy is performed to assess tumor recurrence if necessary. If abnormal elevations of CEA or CA19-9 are found in laboratory tests to suspect recurrence, unscheduled imaging or endoscopy could be performed for confirmation until disease recurrence, withdrawal of informed consent, death of the patient, or termination of the study by the initiator, whichever comes first. MRI or CT imaging is performed every 24 weeks (± 14 days) after 36 months, and enteroscopy is performed to assess tumor recurrence if necessary;

19. Tumor pathological evaluation: surgical resection specimens are evaluated for pathological response;
20. JS001 administered: JS001 240 mg is administered every two weeks in the neoadjuvant phase, and every 3 weeks for each cycle in the adjuvant phase. A maximum delay of 7 days is permitted if the patient is unable to take the medication at the scheduled time for special reasons such as adverse reactions. If patient is still unable to take medication after 7 days delay, skip this dose and the patient should still perform the next visit according to the original plan (the next visit time calculated based on C1D1). If patient discontinue for more than 56 days and if the risk of continuing treatment outweighs the benefit at investigator 's discretion, consider permanent withdrawal from study treatment;
21. All medications taken within 28 days prior to the patient signing the ICF must be recorded on the case report form (CRF), including the generic name of the medication, daily dose, reason for use of the medication, start date, and end date;
22. Adverse events (AEs) should be collected from the time of signing the ICF until 60 days after the last dose of study drug or the start of a new anticancer therapy, whichever occurs first;
23. Survival follow-up: Patients with radiologically confirmed disease recurrence will be followed for survival every 3 months (± 7 days) using the end of treatment visit as the starting point, and information on subsequent anti-tumor treatment and survival will be collected until death, withdrawal of informed consent, loss to follow-up, or study termination by the sponsor, whichever occurs first.

Appendix 2 Eastern Cooperative Oncology Group (ECOG) Score Sheet

Grading	Criteria
0	Completely normal, able to perform all normal activities without restriction<br>(Karnofsky 90 to 100)
1	Unable to carry out strenuous physical activity but ambulatory and able to carry out light physical activity or office work<br>(Karnofsky 70 to 80)
2	Ambulatory and capable of all self-care but unable to carry out any work activities and confined to bed 50% during daytime<br>(Karnofsky 50 to 60)
3	Capable of only limited self-care, confined to bed or chair more than 50% of waking hours<br>(Karnofsky 30 - 40)
4	Completely disabled; cannot carry on any selfcare; totally confined to bed or chair<br>(Karnofsky 10 to 20)

Appendix 3 Response Evaluation Criteria in Solid Tumors

Response Evaluation Criteria in Solid Tumors Version 1.1 (RECIST v1.1): Since there is no official Chinese version of RECIST v1.1, this version is a translated version in Chinese. For more details, please refer to the English version http://ctep.cancer.gov/protocolDevelopment/docs/recist_guideline.pdf.

Summary Background

Assessment of the change in tumor burden is an important feature of the clinical evaluation of cancer therapeutics: both tumor shrinkage (objective response) and disease progression are useful endpoints in clinical trials. Since RECIST was published in 2000, many investigators, cooperative groups, industry and government authorities have adopted these criteria in the assessment of treatment outcomes. However, a number of questions and issues have arisen which have led to the development of a revised RECIST guideline (version 1.1). Evidence for changes, summarized in separate papers in this special issue, has come from assessment of a large data warehouse (>6500 patients), simulation studies and literature reviews.

Important revisions of RECIST 1.1

Major changes include:

Number of lesions to be assessed: based on evidence from numerous trial databases merged into a data warehouse for analysis purposes,the number of lesions required to assess tumor burden for response determination has been reduced from a maximum of 10 to a maximum of five total (and from five to two per organ, maximum). Assessment of pathological lymph nodes is now incorporated: nodes with a short axis of ≥15 mm are considered measurable and assessable as target lesions.The short axis measurement should be included in the sum of lesions in calculation of tumor response. Nodes that shrink to <10 mm short axis are considered normal.

Confirmation of response is required for trials with response primary endpoint but is no longer required in randomised studies since the control arm serves as appropriate means of interpretation of data. Disease progression is clarified in several aspects: in addition to the previous definition of progression in target disease of 20% increase in sum, a 5 mm absolute increase is now required as well to guard against over calling PD when the total sum is very small. Furthermore, there is guidance offered on what constitutes ‘unequivocal progression’ of non-measurable/non-target disease, a source of confusion in the original RECIST guideline. Finally, a section on detection of new lesions, including the interpretation of FDG-PET scan assessment is included. Imaging guidance: the revised RECIST includes a new imaging appendix with updated recommendations

on the optimal anatomical assessment of lesions.

Future work

A key question considered by the RECIST Working Group in developing RECIST 1.1 was whether it was appropriate to move from anatomic unidimensional assessment of tumor burden to either volumetric anatomical assessment or to functional assessment with PET or MRI. It was concluded that, at present, there is not sufficient standardization or evidence to abandon anatomical assessment of tumor burden. The only exception to this is in the use of FDG-PET imaging as an adjunct to determination of progression. As is detailed in the final paper in this special issue, the use of these promising newer approaches requires appropriate clinical validation studies.

1. Background

1.1. History of RECIST Criteria

Assessment of the change in tumor burden is an important feature of the clinical evaluation of cancer therapeutics. Both tumor shrinkage (objective response) and time to the development of disease progression are important endpoints in cancer clinical trials. The use of tumor regression as the endpoint for phase II trials screening new agents for evidence of anti-tumor effect is supported by years of evidence suggesting that, for many solid tumors, agents which produce tumor shrinkage in a proportion of patients have a reasonable (albeit imperfect) chance of subsequently demonstrating an improvement in overall survival or other time to event measures in randomised phase III studies. At the current time objective response carries with it a body of evidence greater than for any other biomarker supporting its utility as a measure of promising treatment effect in phase II screening trials. Furthermore, at both the phase II and phase III stage of drug development, clinical trials in advanced disease settings are increasingly utilising time to progression (or progression-free survival) as an endpoint upon which efficacy conclusions are drawn, which is also based on anatomical measurement of tumor size.

However, both of these tumor endpoints, objective response and time to disease progression, are useful only if based on widely accepted and readily applied standard criteria based on anatomical tumor burden. In 1981 the World Health Organization (WHO) first published tumor response criteria, mainly for use in trials where tumor response was the primary endpoint. The WHO criteria introduced the concept of an overall assessment of tumor burden by summing the products of bidimensional lesion measurements and determined response to therapy by evaluation

of change from baseline while on treatment.

However, in the decades that followed their publication, cooperative groups and pharmaceutical companies that used the WHO criteria often ‘modified’ them to accommodate new technologies or to address areas that were unclear in the original document. This led to confusion in interpretation of trial results6 and in fact,the application of varying response criteria was shown to lead to very different conclusions about the efficacy of the same regimen. In response to these problems, an International Working Party was formed in the mid 1990s to standardize and simplify response criteria. New criteria, known as RECIST (Response Evaluation Criteria in Solid Tumors), were published in 2000. Key features of the original RECIST include definitions of minimum size of measurable lesions, instructions on how many lesions to follow (up to 10; a maximum five per organ site), and the use of unidimensional, rather than bidimensional, measures for overall evaluation of tumor burden. These criteria have subsequently been widely adopted by academic institutions, cooperative groups, and industry for trials where the primary endpoints are objective response or progression. In addition, regulatory authorities accept RECIST as an appropriate guideline for these assessments.

2. Purpose of this guidance:

This guideline describes a standard approach to solid tumor measurement and definitions for objective assessment of change in tumor size for use in adult and paediatric cancer clinical trials. It is expected these criteria will be useful in all trials where objective response is the primary study endpoint,as well as in trials where assessment of stable disease, tumor progression or time to progression analyses are undertaken, since all of these outcome measures are based on an assessment of anatomical tumor burden and its change on study. There are no assumptions in this paper about the proportion of patients meeting the criteria for any of these endpoints which will signal that an agent or treatment regimen is active: those definitions are dependent on type of cancer in which a trial is being undertaken and the specific agent(s) under study. Protocols must include appropriate statistical sections which define the efficacy parameters upon which the trial sample size and decision criteria are based. In addition to providing definitions and criteria for assessment of tumor response, this guideline also makes recommendations regarding standard reporting of the results of trials that utilize tumor response as an endpoint. While these guidelines may be applied in malignant brain tumor studies, there are also separate criteria published for response assessment in that setting.

This guideline is not intended for use for studies of malignant lymphoma since international

guidelines for response assessment in lymphoma are published separately. Finally, many oncologists in their daily clinical practice follow their patients' malignant disease by means of repeated imaging studies and make decisions about continued therapy on the basis of both objective and symptomatic criteria. It is not intended that these RECIST guidelines play a role in that decision making, except if determined appropriate by the treating oncologist.

3. Baseline tumor measurements

3.1 Definitions

At baseline, tumor lesions/lymph nodes will be categorized measurable versus non-measurable as follows

3.1.1 Measurable

Tumor lesions: Must be accurately measured in at least one dimension (longest diameter in the plane of measurement is to be recorded) with a minimum size of:

• 10 mm with CT scan (CT scan slice thickness no greater than 5 mm).

• 10mm caliper measurement by clinical exam (lesions which cannot be accurately measured with calipers should be recorded as non-measurable).

• 20 mm by chest X ray.

Malignant lymph nodes: To be considered pathologically enlarged and measurable, a lymph node must be P15mm in short axis when assessed by CT scan (CT scan slice thickness recommended to be no greater than 5 mm). At baseline and in follow-up, only the short axis will be measured and followed. See also notes below on 'Baseline documentation of target and non-target lesions' for information on lymph node measurement.

3.1.2 Non-measurable

All other lesions, including small lesions (longest diameter <10mm or pathological lymph nodes with ≥10 to <15mm short axis) as well as truly non-measurable lesions. Lesions considered truly non-measurable include: leptomeningeal disease, ascites, pleural or pericardial effusion, inflammatory breast disease, lymphangitic involvement of skin or lung, abdominal masses/abdominal organomegaly identified by physical exam that is not measurable by reproducible imaging techniques.

3.1.3 Special considerations regarding lesion measurability Bone lesions, cystic lesions, and lesions previously treated with local therapy require particular comment:

Bone lesions:

- Bone scans, PET scan or plain films are not considered adequate imaging techniques to measure bone lesions. However, these techniques can be used to confirm the presence or disappearance of bone lesions.
- Lytic bone lesions or mixed lytic-blastic lesions, with identifiable soft tissue components, that can be evaluated by cross sectional imaging techniques such as CT or MRI can be considered as measurable lesions if the soft tissue component meets the definition of measurability described above.
- Blastic bone lesions are non-measurable.

Cystic lesions:

- Lesions that meet the criteria for radiographically defined simple cysts should not be considered as malignant lesions (neither measurable nor non-measurable) since they are, by definition, simple cysts.
- ‘Cystic lesions’ thought to represent cystic metastases can be considered as measurable lesions, if they meet the definition of measurability described above. However, if noncystic lesions are present in the same patient, these are preferred for selection as target lesions.

Lesions with prior local treatment:

- Tumor lesions situated in a previously irradiated area, or in an area subjected to other loco-regional therapy, are usually not considered measurable unless there has been demonstrated progression in the lesion. Study protocols should detail the conditions under which such lesions would be considered measurable.

3.2 Specifications by methods of measurements

3.2.1 Measurement of lesions

All measurements should be recorded in metric notation, using calipers if clinically assessed. All baseline evaluations should be performed as close as possible to the treatment start and never more than 4 weeks before the beginning of the treatment.

3.2.2 Method of assessment

The same method of assessment and the same technique should be used to characterise each identified and reported lesion at baseline and during follow-up. Imaging based evaluation should always be done rather than clinical examination unless the lesion(s) being followed cannot be imaged but are assessable by clinical exam.

Clinical lesions: Clinical lesions will only be considered measurable when they are superficial

and P10mm diameter as assessed using calipers (e.g. skin nodules). For the case of skin lesions, documentation by colour photography including a ruler to estimate the size of the lesion is suggested. As noted above, when lesions can be evaluated by both clinical exam and imaging, imaging evaluation should be undertaken since it is more objective and may also be reviewed at the end of the study.

Chest X Ray: Chest CT is preferred over chest X-ray, particularly when progression is an important endpoint, since CT is more sensitive than X-ray, particularly in identifying new lesions. However, lesions on chest X-ray may be considered measurable if they are clearly defined and surrounded by aerated lung.

CT, MRI: CT is the best currently available and reproducible method to measure lesions selected for response assessment. This guideline has defined measurability of lesions on CT scan based on the assumption that CT slice thickness is 5mm or less. When CT scans have slice thickness greater than 5 mm, the minimum size for a measurable lesion should be twice the slice thickness. MRI is also acceptable in certain situations (e.g. for body scans).

Ultrasound: Ultrasound is not useful in assessment of lesion size and should not be used as a method of measurement. Ultrasound examinations cannot be reproduced in their entirety for independent review at a later date and, because they are operator dependent, it cannot be guaranteed that the same technique and measurements will be taken from one assessment to the next. If new lesions are identified by ultrasound in the course of the study, confirmation by CT or MRI is advised. If there is concern about radiation exposure at CT, MRI may be used instead of CT in selected instances.

Endoscopy, laparoscopy: The utilisation of these techniques for objective tumor evaluation is not advised. However, they can be useful to confirm complete pathological response when biopsies are obtained or to determine relapse in trials where recurrence following complete response or surgical resection is an endpoint.

Tumor markers: Tumor markers alone cannot be used to assess objective tumor response. If markers are initially above the upper normal limit, however, they must normalise for a patient to be considered in complete response. Because tumor markers are disease specific, instructions for their measurement should be incorporated into protocols on a disease specific basis. Specific guidelines for both CA-125 response (in recurrent ovarian cancer) and PSA response (in recurrent prostate cancer), have been published. In addition, the Gynecologic Cancer Intergroup has developed CA125 progression criteria which are to be integrated with objective tumor assessment

for use in first-line trials in ovarian cancer.

Cytology, histology: These techniques can be used to differentiate between PR and CR in rare cases if required by protocol (for example, residual lesions in tumor types such as germ cell tumors, where known residual benign tumors can remain).When effusions are known to be a potential adverse effect of treatment (e.g. with certain taxane compounds or angiogenesis inhibitors), the cytological confirmation of the neoplastic origin of any effusion that appears or worsens during treatment can be considered if the measurable tumor has met criteria for response or stable disease in order to differentiate between response (or stable disease) and progressive disease.

4. Tumor response evaluation

4.1 Assessment of overall tumor burden and measurable disease

To assess objective response or future progression, it is necessary to estimate the overall tumor burden at baseline and use this as a comparator for subsequent measurements. Only patients with measurable disease at baseline should be included in protocols where objective tumor response is the primary endpoint. Measurable disease is defined by the presence of at least one measurable lesion (as detailed above in Section 3). In studies where the primary endpoint is tumor progression (either time to progression or proportion with progression at a fixed date), the protocol must specify if entry is restricted to those with measurable disease or whether patients having non-measurable disease only are also eligible.

4.2 Baseline documentation of 'target' and 'non-target' lesions

When more than one measurable lesion is present at baseline all lesions up to a maximum of five lesions total (and a maximum of two lesions per organ) representative of all involved organs should be identified as target lesions and will be recorded and measured at baseline (this means in instances where patients have only one or two organ sites involved a maximum of two and four lesions respectively will be recorded).

Target lesions should be selected on the basis of their size (lesions with the longest diameter), be representative of all involved organs, but in addition should be those that lend themselves to reproducible repeated measurements. It may be the case that, on occasion, the largest lesion does not lend itself to reproducible measurement in which circumstance the next largest lesion which can be measured reproducibly should be selected.

Lymph nodes merit special mention since they are normal anatomical structures which may be visible by imaging even if not involved by tumor. As noted in Section 3, pathological nodes which are defined as measurable and may be identified as target lesions must meet the criterion of

a short axis of P15mm by CT scan. Only the short axis of these nodes will contribute to the baseline sum. The short axis of the node is the diameter normally used by radiologists to judge if a node is involved by solid tumor. Nodal size is normally reported as two dimensions in the plane in which the image is obtained (for CT scan this is almost always the axial plane; for MRI the plane of acquisition may be axial, sagittal or coronal). The smaller of these measures is the short axis. For example, an abdominal node which is reported as being 20mm· 30mm has a short axis of 20mm and qualifies as a malignant, measurable node. In this example, 20mm should be recorded as the node measurement. All other pathological nodes (those with short axis ⩾10mm but <15 mm) should be considered non-target lesions. Nodes that have a short axis <10mm are considered non-pathological and should not be recorded or followed.

A sum of the diameters (longest for non-nodal lesions, short axis for nodal lesions) for all target lesions will be calculated and reported as the baseline sum diameters. If lymph nodes are to be included in the sum, then as noted above, only the short axis is added into the sum.

The baseline sum diameters will be used as reference to further characterise any objective tumor regression in the measurable dimension of the disease.

All other lesions (or sites of disease) including pathological lymph nodes should be identified as non-target lesions and should also be recorded at baseline. Measurements are not required and these lesions should be followed as ‘present’, ‘absent’, or in rare cases ‘unequivocal progression’ (more details to follow). In addition, it is possible to record multiple nontarget lesions involving the same organ as a single item on the case record form (e.g. ‘multiple enlarged pelvic lymph nodes’ or ‘multiple liver metastases’).

4.3 Response criteria

This section provides the definitions of the criteria used to determine objective tumor response for target lesions..

4.3.1 Response evaluation of target lesions

Complete Response (CR): Disappearance of all target lesions. Any pathological lymph nodes (whether target or non-target) must have reduction in short axis to<10 mm.

Partial Response (PR): At least a 30% decrease in the sum of diameters of target lesions, taking as reference the baseline sum diameters. Progressive Disease (PD): At least a 20% increase in the sum of diameters of target lesions, taking as reference the smallest sum on study (this includes the baseline sum if that is the smallest on study). In addition to the relative increase of

20%, the sum must also demonstrate an absolute increase of at least 5 mm. (Note: the appearance of one or more new lesions is also considered progression).

Stable Disease (SD): Neither sufficient shrinkage to qualify for PR nor sufficient increase to qualify for PD, taking as reference the smallest sum diameters while on study.

4.3.2 Special notes on the assessment of target lesions

Lymph nodes:

Lymph nodes identified as target lesions should always have the actual short axis measurement recorded (measured in the same anatomical plane as the baseline examination),even if the nodes regress to below 10mm on study. This means that when lymph nodes are included as target lesions, the ‘sum’ of lesions may not be zero even if complete response criteria are met, since a normal lymph node is defined as having a short axis of <10mm. Case report forms or other data collection methods may therefore be designed to have target nodal lesions recorded in a separate section where, in order to qualify for CR, each node must achieve a short axis <10mm. For PR, SD and PD, the actual short axis measurement of the nodes is to be included in the sum of target lesions.

Target lesions that are too small to measure:

While on study, all lesions (nodal and non-nodal) recorded at baseline should have their actual measurements recorded at each subsequent evaluation, even when very small (e.g. 2mm). However, sometimes lesions or lymph nodes which are recorded as target lesions at baseline become so faint on CT scan that the radiologist may not feel comfortable assigning an exact measure and may report them as being ‘too small to measure’. When this occurs it is important that a value be recorded on the case report form. If it is the opinion of the radiologist that the lesion has likely disappeared, the measurement should be recorded as 0mm. If the lesion is believed to be present and is faintly seen but too small to measure, a default value of 5mm should be assigned (Note: It is less likely that this rule will be used for lymph nodes since they usually have a definable size when normal and are frequently surrounded by fat such as in the retroperitoneum; however, if a lymph node is believed to be present and is faintly seen but too small to measure, a default value of 5mmshould be assigned in this circumstance as well). This default value is derived from the 5mm CT slice thickness (but should not be changed with varying CT slice thickness). The measurement of these lesions is potentially non-reproducible, therefore providing this default value will prevent false responses or progressions based upon measurement error. To reiterate, however,

if the radiologist is able to provide an actual measure, that should be recorded, even if it is below 5mm.

Lesions that split or coalesce on treatment:

When non-nodal lesions ‘fragment’, the longest diameters of the fragmented portions should be added together to calculate the target lesion sum. Similarly, as lesions coalesce, a plane between them may be maintained that would aid in obtaining maximal diameter measurements of each individual lesion. If the lesions have truly coalesced such that they are no longer separable, the vector of the longest diameter in this instance should be the maximal longest diameter for the ‘coalesced lesion’.

4.3.3 Evaluation of non-target lesions

This section provides the definitions of the criteria used to determine the tumor response for the group of non-target lesions.

While some non-target lesions may actually be measurable, they need not be measured and instead should be assessed only qualitatively at the time points specified in the protocol.

Complete Response (CR): Disappearance of all non-target lesions and normalisation of tumor marker level. All lymph nodes must be non-pathological in size (<10mm short axis).

Non-CR/Non-PD: Persistence of one or more non-target lesion(s) and/or maintenance of tumor marker level above the normal limits.

Progressive Disease (PD): Unequivocal progression (see comments below) of existing non-target lesions. (Note: the appearance of one or more new lesions is also considered progression).

4.3.4 Special notes on assessment of progression of nontarget lesions

When the patient also has measurable disease. In this setting, to achieve ‘unequivocal progression’ on the basis of the non-target disease, there must be an overall level of substantial worsening in non-target disease such that, even in presence of SD or PR in target disease, the overall tumor burden has increased sufficiently to merit discontinuation of therapy. A modest ‘increase’ in the size of one or more non-target lesions is usually not sufficient to quality for unequivocal progression status. The designation of overall progression solely on the basis of change in non-target disease in the face of SD or PR of target disease will therefore be extremely rare.

When the patient has only non-measurable disease. This circumstance arises in some phase

III trials when it is not a criterion of study entry to have measurable disease. The same general concepts apply here as noted above, however, in this instance there is no measurable disease assessment to factor into the interpretation of an increase in non-measurable disease burden.

Because worsening in non-target disease cannot be easily quantified (by definition: if all lesions are truly non-measurable) a useful test that can be applied when assessing patients for unequivocal progression is to consider if the increase in overall disease burden based on the change in non-measurable disease is comparable in magnitude to the increase that would be required to declare PD for measurable disease: i.e.an increase in tumor burden representing an additional 73% increase in ‘volume’ (which is equivalent to a 20% increase diameter in a measurable lesion). Examples include an increase in a pleural effusion from ‘trace’ to ‘large’, an increase in lymphangitic disease from localised to widespread, or may be described in protocols as ‘sufficient to require a change in therapy’. If ‘unequivocal progression’ is seen, the patient should be considered to have had overall PD at that point. While it would be ideal to have objective criteria to apply to non-measurable disease, the very nature of that disease makes it impossible to do so, therefore the increase must be substantial.

4.3.5 New Lesions

The appearance of new malignant lesions denotes disease progression; therefore, some comments on detection of new lesions are important. There are no specific criteria for the identification of new radiographic lesions; however, the finding of a new lesion should be unequivocal: i.e. not attributable to differences in scanning technique, change in imaging modality or findings thought to represent something other than tumor (for example, some ‘new’ bone lesions may be simply healing or flare of pre-existing lesions). This is particularly important when the patient’s baseline lesions show partial or complete response. For example, necrosis of a liver lesion may be reported on a CT scan report as a ‘new’ cystic lesion, which it is not. A lesion identified on a follow-up study in an anatomical location that was not scanned at baseline is considered a new lesion and will indicate disease progression. An example of this is the patient who has visceral disease at baseline and while on study has a CT or MRI brain ordered which reveals metastases. The patient’s brain metastases are considered to be evidence of PD even if he/she did not have brain imaging at baseline.

If a new lesion is equivocal, for example because of its small size, continued therapy and follow-up evaluation will clarify if it represents truly new disease. If repeat scans confirm there is definitely a new lesion, then progression should be declared using the date of the initial scan.

While FDG-PET response assessments need additional study, it is sometimes reasonable to incorporate the use of FDG-PET scanning to complement CT scanning in assessment of progression (particularly possible ‘new’ disease). New lesions on the basis of FDG-PET imaging can be identified according to the following algorithm:

A negative FDG-PET at baseline, with a positive FDG-PET at follow-up is a sign of PD based on a new lesion.

b. No FDG-PET at baseline and a positive FDG-PET at follow-up:

If the positive FDG-PET at follow-up corresponds to a new site of disease confirmed by CT, this is PD.

If the positive FDG-PET at follow-up is not confirmed as a new site of disease on CT, additional follow-up CT scans are needed to determine if there is truly progression occurring at that site (if so, the date of PD will be the date of the initial abnormal FDG-PET scan).

If the positive FDG-PET at follow-up corresponds to a pre-existing site of disease on CT that is not progressing on the basis of the anatomic images, this is not PD.

4.4 Best overall response evaluation

The best overall response is the best response recorded from the start of the study treatment until the end of treatment taking into account any requirement for confirmation. On occasion a response may not be documented until after the end of therapy so protocols should be clear if post-treatment assessments are to be considered in determination of best overall response. Protocols must specify how any new therapy introduced before progression will affect best response designation. The patient’s best overall response assignment will depend on the findings of both target and non-target disease and will also take into consideration the appearance of new lesions. Furthermore, depending on the nature of the study and the protocol requirements, it may also require confirmatory measurement (see Section 4.6). Specifically, in non-randomised trials where response is the primary endpoint, confirmation of PR or CR is needed to deem either one the ‘best overall response’. This is described further below.

4.4.1 Time Point Response

It is assumed that at each protocol specified time point, a response assessment occurs. Table 1 on the next page provides a summary of the overall response status calculation at each time point for patients who have measurable disease at baseline.

When patients have non-measurable (therefore non-target) disease only, Table 2 is to be used.

4.4.2 Missing assessments and inevaluable designation

When no imaging/measurement is done at all at a particular time point, the patient is not evaluable (NE) at that time point.

If only a subset of lesion measurements is made at an assessment, usually the case is also considered NE at that time point, unless a convincing argument can be made that the contribution of the individual missing lesion(s) would not change the assigned time point response. This would be most likely to happen in the case of PD. For example, if a patient had a baseline sum of 50mm with three measured lesions and at follow-up only two lesions were assessed, but those gave a sum of 80 mm, the patient will have achieved PD status, regardless of the contribution of the missing lesion.

4.4.3 Best overall response: all time points

The best overall response is determined once all the data for the patient is known.

Best response determination in trials where confirmation of complete or partial response IS NOT required: Best response in these trials is defined as the best response across all time points (for example, a patient who has SD at first assessment, PR at second assessment, and PD on last assessment has a best overall response of PR). When SD is believed to be best response, it must also meet the protocol specified minimum time from baseline. If the minimum time is not met when SD is otherwise the best time point response, the patient's best response depends on the subsequent assessments. For example, a patient who has SD at first assessment, PD at second and does not meet minimum duration for SD, will have a best response of PD. The same patient lost to follow-up after the first SD assessment would be considered inevaluable.

Best response determination in trials where confirmation of complete or partial response IS required: Complete or partial responses may be claimed only if the criteria for each are met at a subsequent time point as specified in the protocol (generally 4 weeks later). In this circumstance, the best overall response can be interpreted as in Table 3.

4.4.4 Special notes on response Assessments

When nodal disease is included in the sum of target lesions and the nodes decrease to 'normal' size (<10 mm), they may still have a measurement reported on scans. This measurement should be recorded even though the nodes are normal in order not to overstate progression should it be based on increase in size of the nodes. As noted earlier, this means that patients with CR may not have a total sum of 'zero' on the case report form (CRF).

In trials where confirmation of response is required, repeated 'NE' time point assessments

may complicate best response determination. The analysis plan for the trial must address how missing data/assessments will be addressed in determination of response and progression. For example, in most trials it is reasonable to consider a patient with time point responses of PR-NE-PR as a confirmed response.

Patients with a global deterioration of health status requiring discontinuation of treatment without objective evidence of disease progression at that time should be reported as 'symptomatic deterioration'. Every effort should be made to document objective progression even after discontinuation of treatment. Symptomatic deterioration is not a descriptor of an objective response: it is a reason for stopping study therapy. The objective response status of such patients is to be determined by evaluation of target and non-target disease as shown in Tables 1–3.

Conditions that define 'early progression, early death and inevaluability' are study specific and should be clearly described in each protocol (depending on treatment duration, treatment periodicity).

In some circumstances it may be difficult to distinguish residual disease from normal tissue. When the evaluation of complete response depends upon this determination, it is recommended that the residual lesion be investigated (fine needle aspirate/biopsy) before assigning a status of complete response. FDG-PET may be used to upgrade a response to a CR in a manner similar to a biopsy in cases where a residual radiographic abnormality is thought to represent fibrosis or scarring. The use of FDG-PET in this circumstance should be prospectively described in the protocol and supported by disease specific medical literature for the indication. However, it must be acknowledged that both approaches may lead to false positive CR due to limitations of FDG-PET and biopsy resolution/sensitivity.

Attached Table 1 Time point response: patients with target lesions (+ or - non-target) disease.

**Target lesions**	**Non-target lesions**	**New Lesion**	**Overall response**
CR	CR	Non	CR
CR	Non- CR/Non-progressive disease	Non	PR
CR	Not evaluable	Non	PR
PR	Non-progressive or not fully evaluable	Non	PR
SD	Non-progressive or not fully evaluable	Non	SD
Not fully evaluated	Non-Progression	Non	NE

Disease progression	Any condition	Yes or No	Disease progression
Any condition	Disease progression	Yes or No	Disease progression
Any condition	Any condition	Yes	Disease progression
CR = complete response	PR = partial response	SD = Stable disease	NE = Not evaluable

Attached Table 2 Time Point Response - Patients with non-target disease only

Non-target lesions	New Lesion	Overall response
CR	Non	CR
Non- CR or Non-progressive Disease	Non	Non- CR or Non-progressive Disease
Not fully evaluated	Non	Not evaluable
Equivocal disease progression	Yes or No	Disease progression
Any condition	Yes	Disease progression

Note: For non-target lesions, "non CR/non-progressive disease" refers to efficacy superior to SD . Because SD is increasingly used as an endpoint to evaluate efficacy, efficacy for non CR/non progressive disease is being developed to target no measurable disease.

For equivocal findings of progression (e.g. very small and uncertain new lesions; cystic changes or necrosis in existing lesions), treatment may continue until the next scheduled assessment. If at the next scheduled assessment, progression is confirmed, the date of progression should be the earlier date when progression was suspected.

Attached Table 3 Best overall response requiring confirmation of CR and PR response

Overall response at first time point	Overall response at later time points	Best overall response
CR	CR	CR
CR	PR	SD, Disease Progression or PR [a]
CR	SD	SD if SD lasts long enough, otherwise progressive disease
CR	Disease progression	SD if SD lasts long enough, otherwise progressive disease
CR	NE	SD if SD remains adequate, otherwise NE
PR	CR	PR
PR	PR	PR
PR	SD	SD
PR	Disease progression	SD if SD lasts long enough, otherwise progressive disease
PR	NE	SD if SD remains adequate, otherwise NE
NE	NE	NE

Note: CR = complete response, PR = partial response, SD = stable disease; NE = not evaluable.

Superscript "a": If CR truly emerged at the first time point and any disease emerged at subsequent time points, then even if PR criteria were met for this patient relative to baseline, the response evaluation would remain PD at later time points (as disease would reappear after CR). Best response depends on whether SD occurs within the shortest treatment interval. However, sometimes the first assessment is CR, but subsequent timepoint scans suggest small lesions appear to remain present and in fact the patient should have PR rather than CR at the first timepoint. In this case, the first CR determination should be modified to PR, while the best response is PR.

4.5 Frequency of tumor re-evaluation

Frequency of tumor re-evaluation while on treatment should be protocol specific and adapted to the type and schedule of treatment. However, in the context of phase II studies where the beneficial effect of therapy is not known, follow-up every 6–8 weeks (timed to coincide with the end of a cycle) is reasonable. Smaller or greater time intervals than these could be justified in specific regimens or circumstances. The protocol should specify which organ sites are to be evaluated at baseline (usually those most likely to be involved with metastatic disease for the tumor type under study) and how often evaluations are repeated. Normally, all target and non-target sites are evaluated at each assessment. In selected circumstances certain non-target organs may be evaluated less frequently. For example, bone scans may need to be repeated only when complete response is identified in target disease or when progression in bone is suspected.

After the end of the treatment, the need for repetitive tumor evaluations depends on whether the trial has as a goal the response rate or the time to an event (progression/death). If ‘time to an event’ (e.g. time to progression, disease-free survival, progression-free survival) is the main endpoint of the study, then routine scheduled re-evaluation of protocol specified sites of disease is warranted. In randomised comparative trials in particular, the scheduled assessments should be performed as identified on a calendar schedule (for example: every 6–8 weeks on treatment or every 3–4months after treatment) and should not be affected by delays in therapy, drug holidays or any other events that might lead to imbalance in a treatment arm in the timing of disease assessment.

4.6 Confirmatory measurement/duration of response

4.6.1 Confirmation

In non-randomised trials where response is the primary endpoint, confirmation of PR and CR is required to ensure responses identified are not the result of measurement error. This will also permit appropriate interpretation of results in the context of historical data where response has traditionally required confirmation in such trials. However, in all other circumstances, i.e. in randomised trials (phase II or III) or studies where stable disease or progression are the primary endpoints, confirmation of response is not required since it will not add value to the interpretation

of trial results. However, elimination of the requirement for response confirmation may increase the importance of central review to protect against bias, in particular in studies which are not blinded. In the case of SD, measurements must have met the SD criteria at least once after study entry at a minimum interval (in general not less than 6–8 weeks) that is defined in the study protocol.

4.6.2 Duration of overall response

The duration of overall response is measured from the time measurement criteria are first met for CR/PR (whichever is first recorded) until the first date that recurrent or progressive disease is objectively documented (taking as reference for progressive disease the smallest measurements recorded on study). The duration of overall complete response is measured from the time measurement criteria are first met for CR until the first date that recurrent disease is objectively documented.

4.6.3 Duration of stable disease

Stable disease is measured from the start of the treatment (in randomised trials, from date of randomisation) until the criteria for progression are met, taking as reference the smallest sum on study (if the baseline sum is the smallest, this is the reference for calculation of PD).

The clinical relevance of the duration of stable disease varies in different studies and diseases. If the proportion of patients achieving stable disease for a minimum period of time is an endpoint of importance in a particular trial, the protocol should specify the minimal time interval required between two measurements for determination of stable disease.

Note: The duration of response and stable disease as well as the progression-free survival are influenced by the frequency of follow-up after baseline evaluation. It is not in the scope of this guideline to define a standard follow-up frequency. The frequency should take into account many parameters including disease types and stages, treatment periodicity and standard practice. However, these limitations of the precision of the measured endpoint should be taken into account if comparisons between trials are to be made.

4.7 Progression-free survival/proportion progression-free

4.7.1 Phase II trials

This guideline is focused primarily on the use of objective response endpoints for phase II trials. In some circumstances, ‘response rate’ may not be the optimal method to assess the potential anticancer activity of new agents/regimens. In such cases ‘progression-free survival’ (PFS) or the ‘proportion progression-free’ at landmark time points, might be considered appropriate alternatives

to provide an initial signal of biologic effect of new agents. It is clear, however, that in an uncontrolled trial, these measures are subject to criticism since an apparently promising observation may be related to biological factors such as patient selection and not the impact of the intervention. Thus, phase II screening trials utilising these endpoints are best designed with a randomised control. Exceptions may exist where the behaviour patterns of certain cancers are so consistent (and usually consistently poor), that a non-randomised trial is justifiable. However, in these cases it will be essential to document with care the basis for estimating the expected PFS or proportion progression-free in the absence of a treatment effect.

The following contents include endpoints for Phase III evaluation, independent evaluation, result reporting, etc. See the English version for details.

Appendix 4 Precautions for Anaphylaxis

Equipment Required

- Tourniquet;
- Oxygen;
- Epinephrine for subcutaneous, intravenous and/or endotracheal use according to standard practice;
- Antihistamines;
- Corticosteroids;
- IV fluids, tubing, catheters, and tapes.

Operation

In case of suspected allergic reactions during study drug infusion, the following procedures should be performed:

1. Stop study drug infusion;
2. A tourniquet is applied proximal to the injection site to slow systemic absorption of study drug. Do not block arterial blood flow in the affected limb;
3. Maintain airway patency;
4. Administered antihistamines, epinephrine, or other medications as directed by the physician on duty according to the patient 's condition, continue to observe the patient and record observations.

Appendix 5 Recommendations for replacement therapy for hypothyroidism

Levothyroxine sodium tablets (commonly used trade name Euthyrox) are recommended for patients who develop hypothyroidism associated with study treatment.

The recommended dose in this appendix is the general principle, and the individual daily dose for a patient should be determined based on the results of laboratory tests as well as clinical tests. Because many patients have elevated levels of total thyroxine (T4) and FT4, basal concentrations of thyroid-stimulating hormone (TSH) in serum are a reliable basis for determining treatment. Generally, thyroid hormone therapy should be started with a low dose and gradually increased every 2 to 4 weeks until a full dose is reached. In general, patients who develop hypothyroidism require lifelong medication.

Special care should be taken during the initiation of thyroid hormone therapy in elderly patients, in patients with coronary heart disease, and in patients with severe or prolonged hypothyroidism, selecting a lower initial dose (e.g. 12.5 μg/day) and increasing dose slowly at longer intervals (e.g., 12.5 μg/day every other week).

Patients' TSH levels could not be completely corrected if their final maintenance dose is less than optimal.

Experience has shown that low doses are effective in patients with low body weight as well as in patients with macronodular goiter.

Levothyroxine Sodium Tablets should be administered as a single daily dose of the appropriate liquid (e.g., half a glass of water) on an empty stomach half an hour before breakfast.

Exhibit 4 Recommended Dosage Table

	Dose	50 μg tablet	100 μg Tablets	Dosing method
Initial Dose (After initial dose every 2 to 4 weeks increase 25 to 50 μg until maintenance dose amount)	25 ～ 50 μg	1/2 to 1 tablet	1/4 to 1/2 tablet	Once a day
Maintenance dose	100 ～ 200 μg	2 to 4 tablets	1 to 2 tablets	Once a day

For secondary hypothyroidism, the cause must be determined before replacement therapy with Levothyroxine, and glucocorticoid supplementation should be performed when necessary. Once levothyroxine therapy is established, in case of a change of medicinal products, it is recommended to adjust the dose according to the patient 's clinical response and the results of laboratory tests.

Please refer to the relevant package insert for the rest.

Appendix 6 Bayesian Hierarchical Model (Extension of Berry Method)

In this study, we applied a Bayesian hierarchical model (Bayesian hierarchical model, BHM) with multiple testing control to allow information to be adaptively borrowed from each other in different patient cohorts, thereby improving overall statistical power and automatically controlling I type errors. It is an extension of the BHM model of Berry et. al, (2013).

Annex 6.1 Model and Methods

In a multi-cohort expansion trial with multiple doses and multiple indications, the purpose of the trial is to evaluate the efficacy of a new drug at each dose and indication, and to select the appropriate dose and indication cohort for the next phase of the study.

It is assumed that I doses and J indications will be expanded, i.e. patients with J indications will be enrolled concurrently and assigned to I doses. q_{ij}, n_{ij} and y_{ij} represent the probability of efficacy response, the number of patients enrolled and the number of patients with efficacy response on dose i indication j cohorts, respectively. The goal of the trial is to test if this target drug is effective, for each dose-indication cohort:

$$H_0: q_{ij} \leq q_{j0} \;\; v.s. \quad H_1: q_{ij} > q_{j1},$$

In this regard, we assume the following Bayesian hierarchical model that controls type I error for multiple testing:

$$y_{ij}|n_{ij}, q_{ij} \sim Bin(n_{ij}, q_{ij})$$

$$\mathrm{q_{ij}} = \mathrm{logit}(\theta_{ij})$$

$$\theta_{ij}|\,\lambda_{ij} \sim f_1(\theta_{ij})^{I(\lambda_{ij}=1)} f_2(\theta_{ij})^{I(\lambda_{ij}=2)}$$

$$\lambda_{ij} = \begin{cases} 0; & \text{if } Z_{ij} \leq 0 \\ 1; & \text{if } Z_{ij} > 0 \end{cases}$$

$$Z_{ij} \sim N(\xi_i + \eta_j, 1)$$

$$\xi_i \sim N(\xi_0,\ \sigma_\xi^2), \;\; \eta_j \sim N(\eta_0,\ \sigma_\eta^2)$$

$$\xi_0 \sim N(\mu_1,\ \sigma_1^2), \;\; \eta_0 \sim N(\mu_2,\ \sigma_2^2)$$

Among them, $f_1(\theta_{ij}) \sim \text{Cauchy}(\text{scale} = 2.5) \mathrm{I}(\theta_{ij} < \theta_{0j})$, $f_1(\theta_{ij}) \sim \text{Cauchy}(\text{scale} = 2.5) \mathrm{I}(\theta_{ij} > \theta_{0j})$,$\theta_{0j} = logit^{-1}(q_{j0})$. In the above model, we introduce priors for ξ_0 and η_0 , thus effectively solving the problem of multiple testing type I error control because they influence the desired prior probability of the null hypothesis, according to Scott and Berger (2016) 's theory. The more negative μ_1 and μ_2 are, the greater the degree of control over multiple testing. Moreover, priors of ξ_i and η_j are introduced, respectively, so that information could be borrowed between different dosages and indications, and to different

extents. In this study, $\sigma_{\xi}^2 = \sigma_{\eta}^2 = \sigma_1^2 = \sigma_2^2 = 1$ is taken.

At the end of the trial, the model is tested for hypothesis by a posterior probability size of λ_{ij} : if $\Pr(\lambda_{ij} = 1|\text{data}) > \phi_2$, the treatment is considered effective in the dose-indication cohort. The size ofϕ_2 here directly gives the type I error case, as inferred from Bayes. That is, the posterior probability of type I error, i.e. the cohort (i, j) is rejected, is$1 - \phi_2$.

Attachment 6.2 Simulation Studies

Simulation Settings

The simulation study will be performed according to this test setup. In this trial, JS001 will be administered at a fixed dose in combination with other drugs to patients with colorectal cancer, i.e., there are two cohorts: the rectal cancer cohort and the colon cancer cohort. pCR was assumed to be 35% in the rectal cancer cohort and 10% in the historical control; pCR was 15% in the colon cancer cohort and 3% in the historical control. Type I error rate was set at 5% and Type II error at 20% (i.e. 80% for statistical power). Interim analyses were performed when 8 patients were enrolled by the rectal cancer cohort or 11 patients were enrolled by the colon cancer cohort, and the trial was terminated early if Pr (= 1 | data) < 20%.

The simulation scenario setup and simulation research direction of this study are shown in Table 5 below: Scenario 1 is an invalid scenario (global null), which assumes that both cohorts of colorectal cancer are invalid cohorts; Scenario 2 is an alternative scenario (global alternative), under which both cohorts of colorectal cancer are valid cohorts; Scenarios 3 and 4 are mixed scenarios (mixed), in which, One of the two colorectal cancer cohorts was an invalid cohort and the other was a valid cohort. This study will perform a simulation study from three directions: (1) to compare the statistical power and average sample size between the new method and Simon two-stage design to show the advantages of the new method; and to perform a sensitivity test at different sample sizes for the new method to select an appropriate sample size. According to the type 1 error rate of 0.05 and type 2 error rate of 20%, the sample size required for Simon 's two-stage approach based on the pCR rate setting was as follows: for colon cancer group, 47 cases were enrolled and 1 1 case was enrolled in the first stage; for rectal cancer group, 2 2 cases were enrolled and 8 cases were enrolled in the first stage. Therefore, for a fair comparison, interim analyses for all methods were set at 1 1 for colon cancer or 8 for rectal cancer. (2) After determining the sample size, compare the new method with Berry et. al, (2013) 's BHM method.

Schedule 5 Simulated Scenario Settings and Simulated Study Directions (Rectal Cancer, Colon Cancer)

<table>
<tr><th rowspan="2">Scene</th><th rowspan="2">PCR</th><th colspan="2">Sample Size Settings</th></tr>
<tr><th>Simulated (1)</th><th>Simulated (2)</th></tr>
<tr><td>Scenario 1</td><td>(0.1, 0.03)</td><td rowspan="3">(14, 25), (16, 28), (20, 30), (22, 47)</td><td rowspan="3">(16, 28)</td></tr>
<tr><td>Scenario 2</td><td>(0.35, 0.15)</td></tr>
<tr><td>Scenari</td><td>(0.35, 0.03)</td></tr>
</table>

o 3			
Scenari o 4	(0.1, 0.15)		

Simulation Results

The results of the comparison of the new method with Simon and Berry et. al. (2013) are shown in Figure 2. As can be seen from the figure, the new method (Berry 's extended method) provides better control of Type I errors (new method vs Simon) when the sample size is the same (rectal cancer 22 colon cancer 47) compared to Simon two-stage method Simon : 0.04 vs 0.08 in global null scenario) and have the same or higher statistical power (e.g. in global alternative scenario, the new method achieves 91% > 80% for both groups). For the selection of sample size, in order to meet the statistical power of controlling Type I error at 5% in each group, achieving approximately 80%, and combining the performance in a mixed scenario, we chose to enroll 16 patients in the rectal cancer cohort and the 28 patients in the colon cancer cohort. At this time, the overall Type I error was 4%, the overall power of the trial was 94%, and the statistical power for the rectal and colon cancer cohorts was 84% and 76%, respectively. Compared with Berry 's method, although the new method has slightly lower statistical power under scenario 2, the new method is more effective in controlling the problem of Type I error inflation under mixed scenarios 3 and 4, that is, relative to Berry ' s method, and the new method is less likely to consider an invalid cohort as effective.

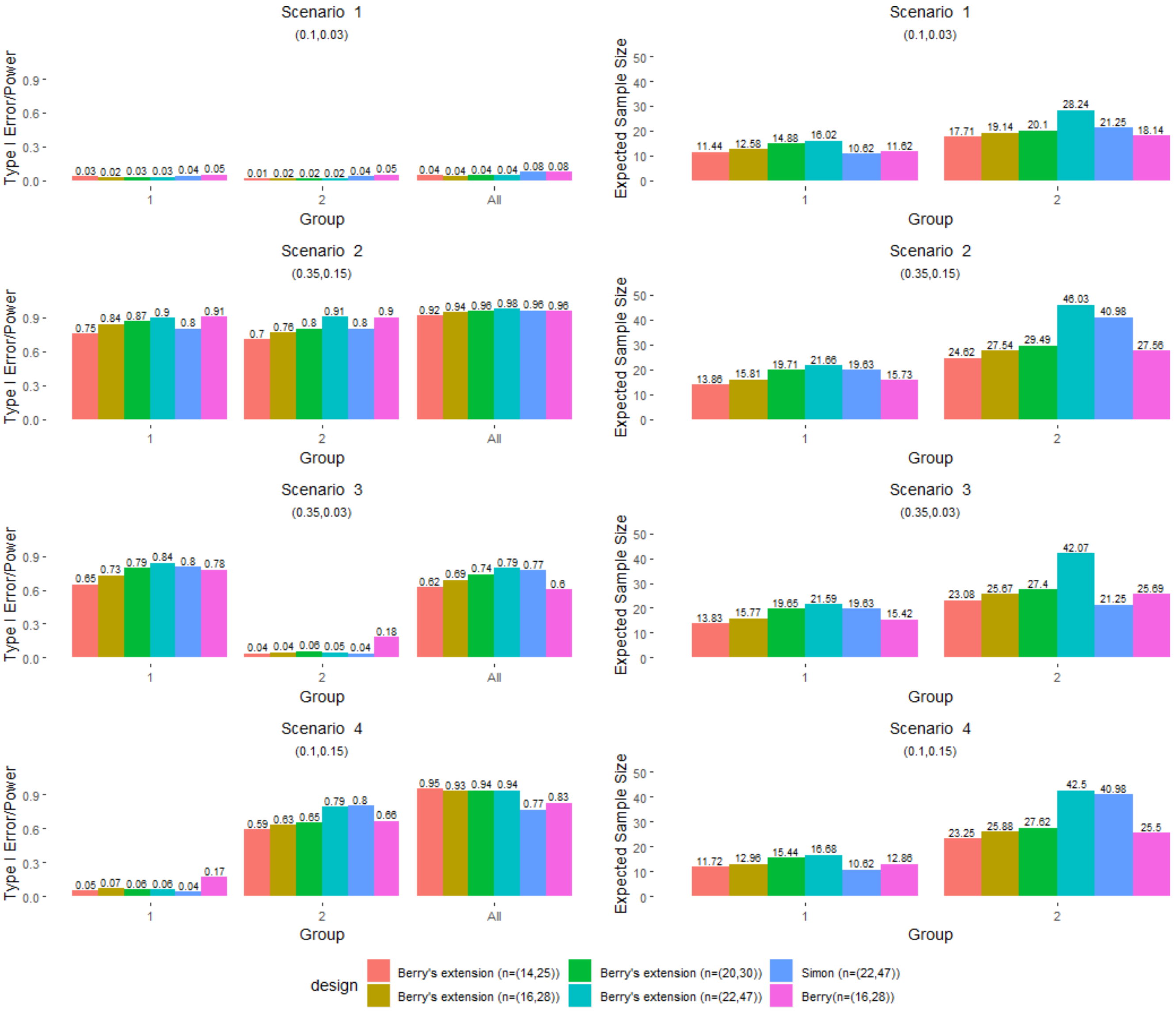

Figure 2 Comparison of new methods (Berry 's Extension), Simon two-stage method, and Berry et. al. (2013) . Group = 1 in the group is the rectal cancer group and group = 2 in the colon cancer group. The left-hand side lists the class of errors and statistical power for each method: group = All represents the overall I class of errors and power, defined as the probability of correctly selecting any valid group, in the alternative scenario; or the probability of incorrectly selecting any invalid group, in the invalid scenario; or the probability of selecting no wrong valid group for the valid group in the mixed scenario. The right side lists the average sample size for both groups.

Appendix 7 EORTC QLQ-C30 Scale

EORTC QLQ-C30 (Version 3)

We would like to learn some information about you and your health. Please answer all questions independently and circle the one that best applies to you. The answer is not divided into "correct" and "wrong". The information you provide will remain strictly confidential.

Please fill in your initials:
Date of Birth (DD/MMM/YYYY):
Today Date (YYYYY, MM, DD):

	**Not at all**	**A little**	**Considerable**	**Very large**
1. Have you had difficulty doing strenuous movements such as lifting heavy shopping bags or suitcases?	1	2	3	4
2. Have you had difficulty walking long distances?	1	2	3	4
3. Did you have trouble taking short walks outdoors?	1	2	3	4
4. Do you have to stay in bed or sit in a chair during the day?	**1**	2	3	4
5. Do you need assistance with eating, dressing, washing or using the toilet?	1	2	3	4
**In the past week** :	**Not at all**	**A little**	**Considerable**	**Very large**
6. Are you physically restricted in your work or daily activities?	1	2	*3*	4
7. Are your hobbies and leisure activities physically restricted?	1	2	3	4
8. Have you had shortness of breath?	1	2	3	4
9. Have you had pain?	**1**	2	3	4
10. Have you ever needed a break?	1	2	3	4
11. Are you having trouble sleeping again?	1	2	3	4
12. Did you feel weak?	1	2	3	4
13. Did you lack appetite?	1	2	3	4
14. Did you feel sick?	1	2	3	4
15. Do you want to vomit?	1	2	3	4
16. Are you constipated?	1	2	3	4

Continued on next page

Appendix 7

EORTC QLQ-C30 (Continued)

**In the past week** :	**Not at all**	**A little**	**Considerable**	**Very large**
17. Have you ever had diarrhea?	1	**2**	3	4
18. Have you ever felt tired?	1	**2**	3	4
19. Does pain interfere with your daily activities?	1	2	3	4
20. Are you unable to concentrate on things, such as reading a newspaper or watching TV?	1	2	**3**	4
21. Have you ever been nervous?	1	**2**	3	4
22. Are you worried?	1	**2**	3	4
23. Have you ever felt irritable?	1	2	3	4
24. Have you ever felt depressed?	1	2	3	4
25. Have you ever had difficulty remembering things?	1	**2**	3	4
26. Does your physical condition or treatment interfere with your family life?	1	2	3	4
27. Does your physical condition or treatment interfere with your social activities?	1	**2**	3	4
28. Has your physical condition or treatment caused you financial difficulties?	1	**2**	3	4

Please circle the most appropriate response between 1 and 7 for the following questions

29. How would you rate your overall health over the past week?

1	2	3	4	5	6	*7*
Very Poor						Very good

30. How would you rate your overall quality of life over the past week?

1	2	3	4	5	6	7
Very Poor						Very good

© Copyright reserved to 1995 EORTC Quality of Life Group. All rights reserved. All rights reserved. Version 3.0

Appendix 8 Questionnaire EORTC QLQ-CR29

EORTC QLQ – CR29

Patients sometimes complain that they have the following symptoms or problems. Please indicate the extent to which you have experienced these symptoms or problems during the past week. Please circle the number that best suits you to answer the question.

**In the past week:**		**None**	**A little**	**Quite**	**very**
31.	Did you urinate frequently during the day?	1	2	3	4
32.	Have you had frequent urination at night?	1	2	3	4
33.	Have you had involuntary urination (leakage)?	1	2	3	4
34.	Have you had pain urinating?	1	2	3	4
35.	Have you had abdominal pain?	1	2	3	4
36.	Have you had pain in your buttocks/anal area/rectum?	1	2	3	4
37.	Have you had abdominal fullness?	1	2	3	4
38.	Have you had blood in your stool?	1	2	3	4
39.	Did you have mucus in your stool?	1	2	3	4
40.	Did you get dry?	1	2	3	4
41.	Have you had hair loss caused by treatment?	1	2	3	4
42.	Have you had a problem with your taste?	1	2	3	4

**In the past week:**		**None**	**Somewhat**	**very**	
43.	Are you concerned about your future health?	1	2	3	4
44.	Have you worried about your weight?	1	2	3	4
45.	Have you felt that your physical attractiveness decreased as a result of your disease or treatment?	1	2	3	4
46.	Do you feel that the disease or treatment reduces your femininity/masculinity?	1	2	3	4
47.	Have you felt dissatisfied with your body?	1	2	3	4

48. Do you have an ostomy bag (colostomy/ileostomy)?
(Please circle the correct answer) Yes None

Please continue next page

In the past week:

	None	A little	Comparable	Very
**Answer these questions only if you have a pocket or continue with the following questions:**				
49. Have you had involuntary pocket venting/flatulence?	1	2	3	4
50. Have you had any stool leaking from your ostomy bag?	1	2	3	4
51. Have you had any pain in the skin around your stoma?	1	2	3	4
52. Have you changed your bags frequently during the day?	1	2	3	4
53. Have you changed your bags frequently during the night?	1	2	3	4
54. Have you felt embarrassed about having a stoma?	1	2	3	4
55. Have you had trouble caring for your stoma?	1	2	3	4

**Answer these questions only if you do not have a bag:**				
49. Have you had involuntary flatus/gas?	1	2	3	4
50. Did you leak stool from your anus?	1	2	3	4
51. Have you had perianal skin pain?	1	2	3	4
52. Have you had frequent bowel movements during the day?	1	2	3	4
53. Have you had frequent bowel movements at night?	1	2	3	4
54. Have you felt embarrassed about passing stools?	1	2	3	4

Over the past 4 weeks:

	None	A little	Comparable	Very
**For males only:**				
56. How interested are you in having sex?	1	2	3	4
57. Have you had trouble getting or maintaining an erection?	1	2	3	4

**For females only:**				
58. How interested are you in having sex?	1	2	3	4
59. Have you had pain or discomfort during sexual intercourse?	1	2	3	4

© QLQ-CR29 Copyright 2006 EORTC Quality of Life Group. All rights reserved. Version 2.1
